# Combined Protein and Alkaloid Research of *Chelidonium majus* Latex Reveals CmMLP1 Accompanied by Alkaloids with Cytotoxic Potential to Human Cervical Carcinoma Cells

**DOI:** 10.3390/ijms222111838

**Published:** 2021-10-31

**Authors:** Robert Nawrot, Alicja Warowicka, Piotr Józef Rudzki, Oskar Musidlak, Katarzyna Magdalena Dolata, Jacek Musijowski, Elżbieta Urszula Stolarczyk, Anna Goździcka-Józefiak

**Affiliations:** 1 Molecular Virology Research Unit, Institute of Experimental Biology, Faculty of Biology, Adam Mickiewicz University, Poznań, Uniwersytetu Poznańskiego 6, 61-614 Poznań, Poland; oskar.musidlak@amu.edu.pl (O.M.); katarzyna.dolata@fli.de (K.M.D.); agjozef@amu.edu.pl (A.G.-J.); 2 Department of Animal Physiology and Developmental Biology, Institute of Experimental Biology, Faculty of Biology, Adam Mickiewicz University, Poznań, Uniwersytetu Poznańskiego 6, 61-614 Poznań, Poland; alicja@amu.edu.pl; 3 NanoBioMedical Centre, Adam Mickiewicz University, Poznań, Wszechnicy Piastowskiej 3, 61-614 Poznań, Poland; 4 Łukasiewicz Research Network—Pharmaceutical Research Institute, Rydygiera Street 8, 01-793 Warsaw, Poland; pj.rudzki@wp.pl (P.J.R.); j.musijowski@gmail.com (J.M.); elzbieta.stolarczyk@ichp.pl (E.U.S.)

**Keywords:** major latex protein, *Chelidonium majus*, greater celandine, defense-related proteins, alkaloids, molecular docking, cancer cells

## Abstract

*Chelidonium majus* L. is a latex-bearing plant used in traditional folk medicine to treat human papillomavirus (HPV)-caused warts, papillae, and condylomas. Its latex and extracts are rich in many low-molecular compounds and proteins, but there is little or no information on their potential interaction. We describe the isolation and identification of a novel major latex protein (CmMLP1) composed of 147 amino acids and present a model of its structure containing a conserved hydrophobic cavity with high affinity to berberine, 8-hydroxycheleritrine, and dihydroberberine. CmMLP1 and the accompanying three alkaloids were present in the eluted chromatographic fractions of latex. They decreased in vitro viability of human cervical cancer cells (HPV-negative and HPV-positive). We combined, for the first time, research on macromolecular and low-molecular-weight compounds of latex-bearing plants in contrast to other studies that investigated proteins and alkaloids separately. The observed interaction between latex protein and alkaloids may influence our knowledge on plant defense. The proposed toolbox may help in further understanding of plant disease resistance and in pharmacological research.

## 1. Introduction

*Chelidonium majus* L. is a perennial herbal plant belonging to the family Papaveraceae that grows across Europe, western Asia, and North America [1,2,3]. It is a rich source of different biologically active substances (alkaloids, flavonoids, phenolic acids) and has been used in folk medicine for centuries. Over 27 alkaloids (e.g., chelerythrine, sanguinarine, chelidonine, protopine, allocryptopine, berberine, coptisine) were identified in its extracts and latex [4,5]. Plant laticifer cells are filled with latex (milky sap) that contains condensed defense substances [6,7]. The plant exudes latex immediately at the site of damage caused by an insect attack [7,8]. In folk herbal medicine, extracts and latex of *C. majus* are used to treat warts and condylomas caused by human papillomavirus (HPV) [9]. The medicinal interest in *C. majus* is based mainly on small molecules such as alkaloids, flavonoids, and phenolic acids, which may also act synergistically [4,5,10]. These compounds exhibit antitumor, antiviral, and antibacterial activities [5,11]. The proteins of *C. majus* also show mitogenic, cytotoxic, antibacterial, and antiviral activities [7]. Nucleases present in milky sap were found to exert apoptotic effects on a human cervical cancer HeLa cell line [12,13]. The antiviral activity of milky sap was suggested to be associated with major latex proteins (MLPs) [7,14].

MLPs, pathogenesis-related 10 (PR-10) proteins, cytokinin-specific binding proteins (CSBPs), and norcoclaurine synthases belong to the Bet v 1 superfamily of proteins. Their characteristic hydrophobic cavity binds to secondary metabolites and hormones [15,16]. MLPs were discovered in *Papaver somniferum* as abundant, laticifer-specific peptides with unknown function [17]. MLPs constitute up to 50% of the soluble *P. somniferum* latex subproteome, which correlates with the relative abundance suggested by SDS-PAGE [18]. MLPs are also present in non-latex-bearing plants such as *Arabidopsis thaliana* and *Prunus persica* (peach) [15]. They influence fruit ripening in kiwi [16] and fruit and flower development in peach [19]. They protect cotton against *Verticillium dahliae* [20] and melon against cucumber mosaic virus [21]. The presence of MLP-like protein in the latex of *Chelidonium majus* L. was first reported using proteomic analysis against *C. majus* sequence database prepared after transcriptome sequencing and annotation [22]. The study identified previously uncharacterized nucleic acid binding protein and showed that it is highly overrepresented in the latex [22,23]. Statistical analysis confirmed that MLP is present in different stages of plant development until the fruit ripening period [14].

Our interest in *C. majus* originated from the traditional use of the fresh plant extracts and latex against warts and condylomas caused by the oncogenic human papillomavirus (HPV) infection. HPV infections can lead to cervical cancer in women, which can result from persistent infection with a group of “high-risk” HPVs [24]. However, previous studies on *C. majus* have shown that the proteins from this plant are also biologically active [3,13]. The antiviral activity of the *C. majus* milky sap is possibly linked with the presence of MLP [7,25]. Synergistic action of plant secondary metabolites with other components of the plant extracts is also postulated [10].

Hence, the goal of the study was to isolate the MLP from *C. majus* latex, identify accompanying low-molecular compounds and to analyze their joint cytotoxic activities against cervical cancer cell lines.

## 2. Results

**Figure 1:**
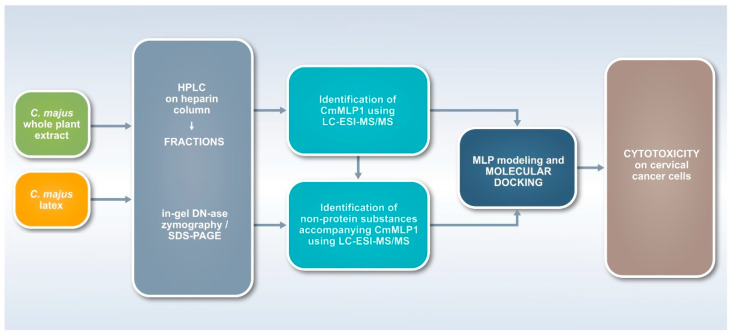
Workflow for the multidisciplinary study (toolbox) for two kinds of plants source materials—*C. majus* extracts and latex. The research scheme enabled fractionation with the use of affinity chromatography, monitoring of all fractions with the use of proteomic, zymography, as well as LC-ESI-MS/MS techniques for the identification of proteins and non-protein substances. Molecular interactions were confirmed with the use of bioinformatic tools of molecular modeling and docking, and their biological activities were finally analyzed using cell lines.

To the best of our knowledge, no joint research has been performed before in separate fields of plant compounds research of different structures and sizes for latex-bearing plants. Such an approach requires multidisciplinary research with the use of biological, biotechnological, chemical, as well as analytical and theoretical techniques (Figure 1). Therefore, we employed a research scheme, which started with the use of two kinds of source materials—*C. majus* whole plant extract and latex samples. The second step enabled fractionation of the material with the use of affinity chromatography on heparin. All fractions were monitored with the use of proteomic and zymography techniques, as well as with the use of LC-ESI-MS/MS techniques for the identification of proteins and non-protein substances. This step allowed to indicate specific protein and alkaloids co-existing in the fractions, which was confirmed with the use of bioinformatic tools of molecular modeling and docking. Finally, the fractions were monitored in terms of their cytotoxic activities on cervical cancer cells (Figure 1).

### 2.1. Isolation of MLP from C. majus Whole Plant Extracts and Latex

**Figure 2:**
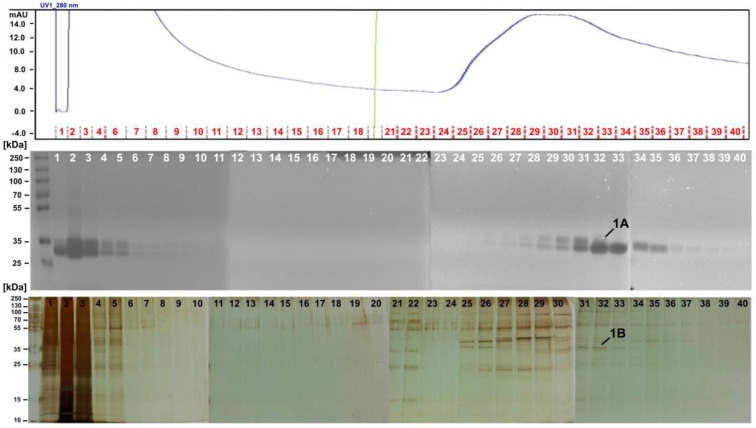
Fractionation of *C. majus* whole plant extract on a heparin column. Top: Absorbance at 280 nm—blue line—indicates protein content in fractions. Red lines and numbers represent fraction numbers. Middle: In-gel DNase assay zymography of protein fractions with high nucleolytic activity. Band 1A of ca. MW 33–35 kDa was cut, and MLP presence was confirmed by MS/MS. Bottom: Protein profiles of each fraction in SDS-PAGE stained with silver. Band 1B of ca. MW 36 kDa was cut from the gel, and MLP presence was confirmed by MS/MS.

To isolate and purify the MLP from whole plant extracts and latex samples, heparin column was selected due to its high affinity to DNA-binding proteins, coagulation factors, lipoproteins, and protein synthesis factors. In-gel DN-ase zymography was used to assess the presence of MLP in separated fractions. We observed a strong nucleolytic activity for the selected fractions eluted (Figure 2). We collected protein bands showing nucleolytic activity and analyzed them by LC-ESI-MS/MS. We then applied Mascot analysis to MS results using the annotated *C. majus* CDS database [22]. We observed that MLP was the main constituent of the nucleolytic bands (MLP-like protein 28, Table 1, band 1A; Table S1). We visualized the protein content of the fractions by using SDS-PAGE and silver staining (Figure 2). The most representative bands were then collected and analyzed by LC-ESI-MS/MS. MLP was again found to be the main constituent of the fractions—both for the whole plant extracts (Table 1, band 1B; Table S2) and latex samples (Figure S1; Table S3).

### 2.2. LC-MS/MS Identification of Non-Protein Substances Accompanying MLP

We washed out the unbound small molecules and proteins with no affinity from the column (Figure 2, fractions 1–18). However, because we also aimed to identify nonprotein substances that might possibly be present in the nucleolytic fractions, we extended our toolbox by adopting sample preparation from plasma/serum pharmacokinetics (Figures S2–S33) and combining it with LC-MS/MS screening of small molecules. In the fractions derived from the whole plant extract, we found the following alkaloids: berberine, chelidonine, coptisine, corysamine, dihydroberberine, dihydrosanguinarine, dihydroxychelerythrine, dihydroxysanguinarine, norchelerythrine, norsanguinarine, and protopine (Figures S3–S21) [4,5]. For samples rich in low-molecular constituents, we semi-quantitatively compared 21 signals (Tables S4 and S5; Figures S34–S47). Finally, we identified 8 alkaloids co-localizing with proteins in the protein-rich fractions. The presence of these alkaloids suggest their molecular association with proteins. The highest relative signal intensities for coptisine, berberine, dihydroberberine, chelidonine, and protopine were found in fractions 22–23 (Figure 3, in green), while those for 8-hydroxycheleritrine, stylopine, and sanguinarine were found in fractions 25–26 (Figure 3, in green).

We used the same approach for *C. majus* milky sap, which enabled to identify several alkaloids (Table S5). Although the proposed identifications were based only on mass spectrometry data, they were consistent with the compounds detected previously in *C. majus* [4,5].

### 2.3. CmMLP1 Domain Architecture

The identified MLP-like protein 28 (Figure 2b) belongs to the MLP-related protein family 28 as described for *A. thaliana* [26]. We conducted bioinformatics analysis by searching for MLP-like sequences in the *C. majus* CDS database derived from transcriptomic data (http://webblast.ipk-gatersleben.de/chelidonium/, accessed on 28 October 2021). The analysis revealed the presence of 19 transcript sequences of length 105–359 nucleotides (Figure 4), coding for MLP-like proteins of different sizes with a common “core” of approximately 100 amino acids (Figure 5 and Figure S48). We selected the sequence composed of 147 amino acids as the one coding for the MLP, with the calculated MW of 16.77 kDa and theoretical pI of 5.88 (MLP-like m.12630); this result was consistent with the size of MLPs derived from other plants [17]. The protein encoded by this sequence was named CmMLP1. By using bioinformatics tools, we predicted the conserved hydrophobic cavity for binding of small molecules in the structure of CmMLP1 and confirmed that the protein belongs to the “START/RHO_alpha_C/PITP/Bet_v1/CoxG/CalC (SRPBCC) ligand-binding domain superfamily” [12,19,27].

### 2.4. MLP Structure Prediction and Modeling

We used a homology modeling approach for constructing 3D protein structure. On the basis of the fold-recognition (FR) alignment proposed by GeneSilico MetaServer, we selected template structures of 4IGV chain A—crystal structure of kirola (Act d 11, PubMed: 23969108). The structure of the protein covers the sequence in the region of 1–101 amino acids. The PROQ method to predict model quality evaluated the model as “fairly good” (predicted LGscore = 1.151, predicted MaxSub = 0.172, predicted GDT_TS: 36.39). Figure 6 and Figure S49 illustrate the predicted quality of the mature protein structure. 

### 2.5. Molecular Docking of Alkaloids to CmMLP1

Eight alkaloid structures retrieved from ZINC database were docked in the hydrophobic pocket of the CmMLP1. Docking of ligand molecules to CmMLP1 was performed using the Autodock Vina program, which analyzes 20 possible conformations of the ligand molecule at the active site of the protein. Binding energy in the active site was calculated for each conformation. We observed high binding affinity for dihydroberberine and 8-hydroxycheleritrine as well as relatively high affinity for berberine (Table 2, nos. 1–3, Tables S6–S13; Figure S50).

### 2.6. Cytotoxic Activity of CmMLP1 Accompanied with Alkaloids

To assess the biological activity of fractions containing CmMLP1 accompanied with alkaloids, we analyzed their cytotoxic effects on HeLa and C33A cell lines. We used two such cell lines—HeLa and C33A. Both of them originate from cervical samples of women, however they differ with the HPV genetic material content. HeLa cells are considered as HPV positive (HPV+), and C33A as HPV negative (HPV-). The analysis was performed using the WST-1 colorimetric assay and normal fibroblasts (MSU-1.1) were used as a control.

We analyzed the whole spectra of the samples separated on heparin column both from whole plant extracts and from latex. The results indicated that the viability of HeLa and C33A cancer cells after 48 h of incubation with all fractions from latex as well whole plant extract was reduced on different levels. These cell lines showed similar viability after treatment with fractions derived from whole plant extract (Figures S51 and S52). However, an immediate decrease of at least 50% in viability was observed for HeLa and C33A cells when treated with latex fractions 24–26 as compared to that for MSU-1.1 (Figure 7). We previously confirmed the presence of CmMLP1 and alkaloids in the same fractions (Table 1). Moreover, the cell viability was the lowest for HeLa cells, with less than 10% living cells, what is comparable to negative control treatments (DMSO). The results suggest that the fractions containing CmMLP1 accompanied by alkaloids present the highest cytotoxic activity against cervical cancer HPV positive cells—less than 10% living cells left for each fraction (Figure 7a,c; Table 3). The viability of the cervical cancer cells was two-fold lower for HPV+ (HeLa) cells than for HPV- (C33A) cells (*p* < 0.0001) (Figure 7a).

## 3. Discussion 

**Figure 3:**
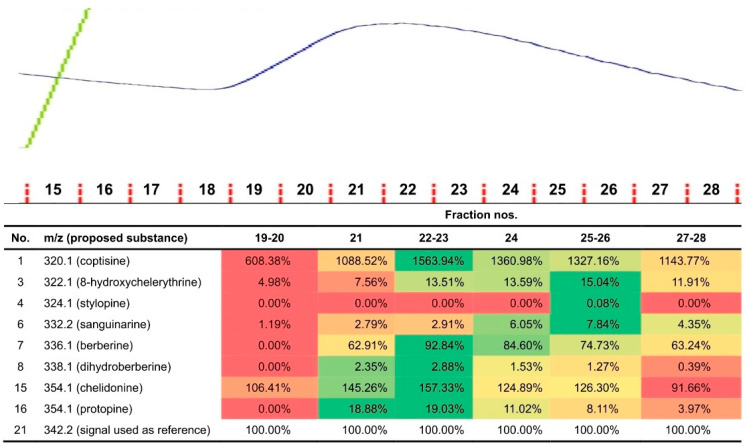
Correlation of the protein content (MLP) with the alkaloid content of the fraction. Top: Chromatogram of *C. majus* latex protein fractions adjusted to the numbers of fractions in the table. Blue line indicates protein content in fraction (monitored at 280 nm). The protein peak between fractions nos. 19–26 presents higher protein content in the fractions, which could be correlated with the higher quantity of identified alkaloids in the same fractions. Bottom: Relative signal intensities for latex fractions. Color coding ranges from red (lowest relative intensity of a particular compound in a group of samples) up to green (highest relative intensity).

**Figure 4:**
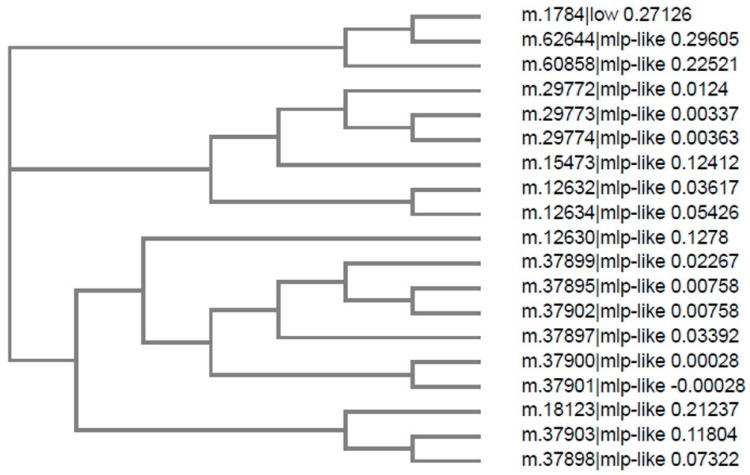
Phylogenetic tree showing relationships between 19 MLP-like sequences from *C. majus*. *C. majus* database accession numbers are shown (http://webblast.ipk-gatersleben.de/chelidonium/, accessed on 28 October 2021).

**Figure 5:**
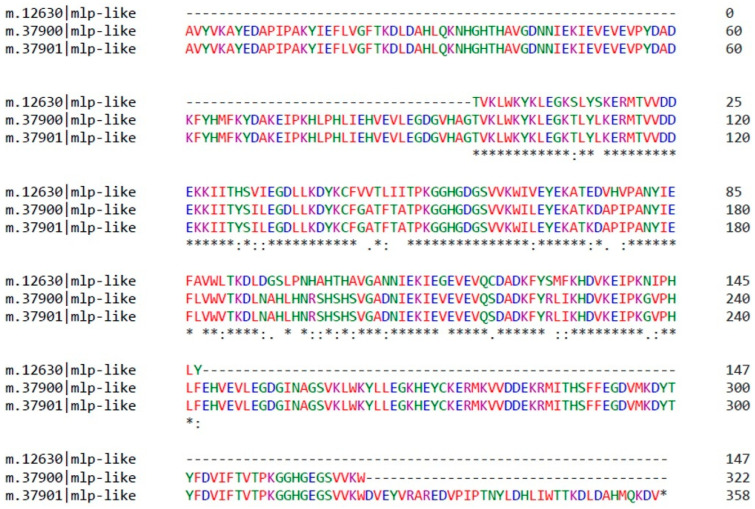
Primary sequence alignment of three *C. majus* MLPs. The protein sequence is shown, with the number of amino acids for each sequence. Asterisks indicate conserved amino acids with the “core” common to all sequences. The shortest MLP-like m.12630 sequence contains 147 residues and has molecular weight of 16.77 kDa. *C. majus* database accession numbers are shown.

**Figure 6:**
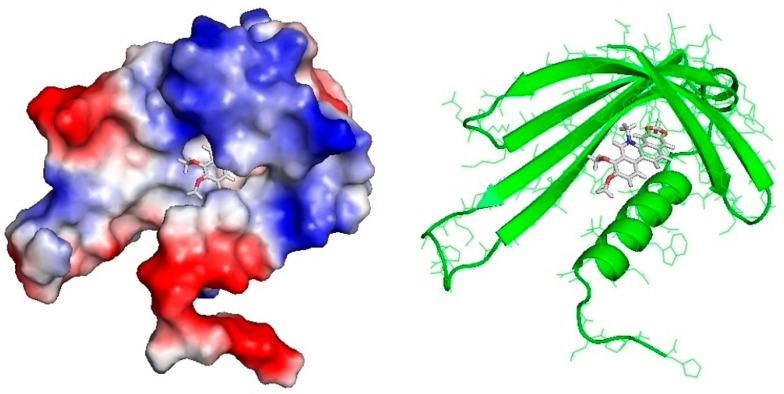
Left: 3D model of *C. majus* MLP with surface representation, colored according to the distribution of the electrostatic surface potential calculated with ABPS (PyMol). Positively charged regions in blue and negatively charged regions in red. Hydrophobic cavity filled by dihydroberberine (docking affinity 5.98). Right: MLP in a ribbon representation with docked 8-hydroxycheleritrine (docking affinity 5.94).

Our present study has enabled us to advance plant science because of multidisciplinary research on compounds of different structures: large and small molecules. MLP was accompanied with coptisine, stylopine, sanguinarine, chelidonine, protopine, berberine, 8-hydroxycheleritrine, and dihydroberberine (Figure 3). Molecular docking showed high affinity of the latter three molecules to the hydrophobic cavity of CmMLP1 (Figure 3d). To the best of our knowledge, these associations are reported for the first time. Previous studies focused solely on either macromolecules or alkaloids. Both these groups of compounds coexist in a plant and interact with each other. Thus, the associations observed here may influence our knowledge of plant defense and function of the latex proteins. They may also further our understanding of their pharmacology. 

**Figure 7:**
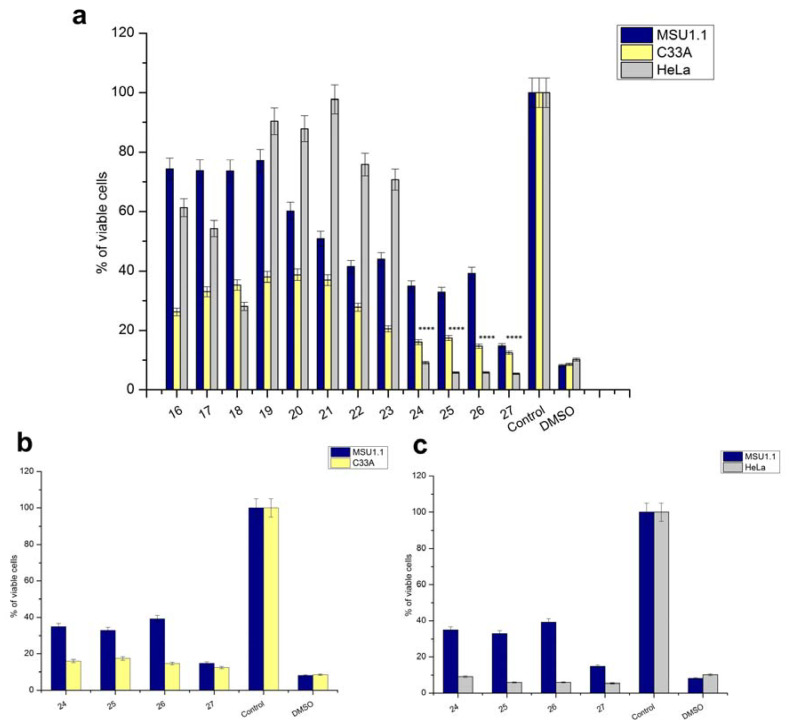
CmMLP1 and the accompanying three alkaloids decreased in vitro viability of human cervical cancer cells (HPV-negative and HPV-positive). (**a**–**c**) Viability (%) of C33A and HeLa cancer cells compared to that of normal MSU1.1 fibroblasts after treatment with latex fractions for 48 h. Control (negative)—untreated cells; DMSO—positive control. (**a**) C33A, HeLa, and MSU-1.1 cell lines treated with fractions 16–27. (**b**) Both C33A HPV- cervical cancer cell line and MSU-1.1 treated with fractions 24–26 containing CmMLP1 accompanied with alkaloids and fraction 27. (**c**) Both HeLa HPV+ cervical cancer cell line and MSU-1.1 treated with fractions 24–26 containing CmMLP1 accompanied with alkaloids and fraction 27. ****, *p* < 0.0001.

The high cytotoxicity of the combination of CmMLP1 and alkaloids for both cancer cell lines (Figure 7b,c) indicates their potential use in cancer therapy. The cell viability of HPV+ cells was twice lower than that of HPV− cells. The biological activity of *C. majus* alkaloids is well described. Cytotoxicity tests demonstrated selective and profound apoptotic effects of a five-alkaloid combination in the mouse melanoma B16F10 cell line [27]. Chelidonine efficiently induced apoptosis in HeLa cells through possible alteration of p38-p53 and AKT/PI3 kinase signaling pathways [28]. Chelidonine was also examined as a potent inducer of cell death in human leukemic and lung carcinoma cells [29], as well as a promising model compound for overcoming multidrug resistance in Caco-2 and CEM/ADR5000 cancer cells together with the alkaloid extract comprising of protoberberine and benzo[c]phenantridine alkaloids [30]. Our findings report that one of the docking alkaloids to CmMLP1, with high binding affinity, is dihydroberberine (DIH). This alkaloid belongs to the protoberberine alkaloid family and presents strong biological activity including anticancer properties. Recently, it has been shown that the components of the natural plant protoberberine fraction (BBR-F) extracted from *C. majus* may represent promising novel photosensitive agents [6]. Increased cytotoxicity of the combination of CmMLP1 with alkaloids for both cancer cell lines—HeLa, C33A, compared to normal MSU-1.1 fibroblasts (Figure 7, Table 3), is of great importance for the potential use in cancer therapy. The cell viability was the lowest for HeLa HPV positive cells—with less than 10% of living cells—two times lower than for C33A HPV negative cells. This may suggest the joint action of alkaloids and CmMLP1 through the unknown mechanism connected to the HPV genome or its protein products [31,32]. This result suggests the synergistic effect of CmMLP1 and alkaloids against HPV genome or its protein products [32]. The mechanism of this effect, however, remains unknown. Low-molecular-weight latex compounds may act synergistically with proteins and influence their conformation. The proteins, in turn, might facilitate transport of small molecules to the cell or act as their transporters [33].

The presence of MLP-like protein in *C. majus* latex was reported for the first time using proteomic analysis against *C. majus* sequence database, prepared after transcriptome sequencing and annotation [22]. Mass spectrometry analysis allowed the identification of MLP in protein bands with nucleolytic activity as their main constituents (MLP-like protein 28, Table 1). Confirmation of the nucleolytic activity of a particular protein of latex is the significant novelty of this study. Previously, nucleolytic bands were not specifically identified as “nucleic acid binding proteins,” “DNA-binding” or “RNA-binding” [12] because of the use of the general NCBI plants database (Viridiplantae), which does not consider the various possibilities. Hence, the nucleolytic activity observed by zymography may be a “marker” of the presence of MLP in other samples from *C. majus*. The remaining question still concerns the possible function of the CmMLP1 in association with alkaloids. Previous studies show that the functional role of MLPs is similar to that of the PR-10 proteins [26]. MLPs and PR-10 families both belong to the Bet v 1 superfamily, but the sequence identity between them is rather low (<25%) [16]. Moreover, CaPR-10 protein isolated from hot pepper acts as an antiviral protein inhibiting viral penetration and/or replication. After tobacco mosaic virus (TMV-P0) inoculation CaPR-10 is phosphorylated and functions as a kind of RNase which cleaves viral RNA [27]. The functional role of MLPs is similar to PR-10 proteins, which could possibly explain *C. majus* latex antiviral activities [7,34]. However, this issue needs further studies.

## 4. Materials and Methods

### 4.1. Collection of Plant Material 

*C. majus* plants were collected in May 2014 from the neighborhood of Poznan, Poland, in compliance with all Adam Mickiewicz University, Polish state and international guidelines and legislation. Plants were identified by Robert Nawrot and the voucher specimen was deposited in the herbal collection of Faculty of Biology, Adam Mickiewicz University, Poznan, Poland with the number CHM_20140514_001 at Molecular Virology Research Unit. The specimen is fully publicly accessible upon request. Both milky sap and aerial parts (stems, leaves, and flowers) were collected from different adult *C. majus* plants of similar developmental stage (height of the plant was ca. 50 cm).

#### 4.1.1. Whole Plant Extracts 

The stems were cut, and the exuding orange milky sap was collected. The samples were directly dissolved in 0.1 M Tris-HCl buffer, pH 8.0, containing 10% glycerol (sap:buffer ratio 1:2). The milky sap (33% *v*/*v*) samples were separated into a supernatant (referred to as a protein fraction) and a pellet fraction by centrifugation at 12,000 rpm for 20 min at 4 °C. Plant tissue samples stored at −80 °C were frozen in liquid nitrogen and powdered in a grinder. Each sample (200 mg) was directly dissolved in 1 mL of 0.1 M Tris-HCl buffer, pH 8.0, containing 10% glycerol.

#### 4.1.2. Milky Sap 

The stems of *C. majus* plants were cut, the surface was dried with a filter paper, and the exuding orange milky sap was collected using plastic micropipettes. Depending on the plant and the sampling site, up to 500 µL sample was collected from one stem by using this method. The samples were dissolved in a buffer in a ratio of 1:2. Both extract and milky sap samples were separated into a supernatant (referred to as a protein extract) and a pellet fraction by centrifugation at 12,000 rpm for 20 min at 4 °C. Supernatants were stored at −20 °C for further analysis.

### 4.2. Protein Separation

Sodium dodecyl sulfate-polyacrylamide gel electrophoresis (SDS-PAGE) was performed in a slab mini-gel apparatus [35] by using 10% polyacrylamide as a separating gel and 5% polyacrylamide as a stacking gel. The proteins were reduced at 100 °C in the presence of 2-mercaptoethanol for 5 min. The gels were then fixed and stained with silver [36]. In-gel DNase assay (zymography) was applied to crude milky sap and whole plant extract samples. The samples were loaded onto SDS-polyacrylamide gels with embedded DNA, and an in-gel DNase assay was performed [36,37]. Extracts and fractions after purification were dissolved in SDS-PAGE sample buffer without a reducing agent (1:1), incubated at 37 °C for 10 min, and subjected to SDS-PAGE in a 10% polyacrylamide gel containing denatured calf thymus DNA (40 μg/mL). Electrophoresis was run at 100 V and 25 mA at 4 °C for approximately 3 h. To remove SDS, the gels were soaked in 25% isopropanol 2× for 20 min at room temperature. The gels were then washed with a renaturation buffer (10 mM Tris-HCl, pH 8.0, containing 10 mM CaCl_2_) 1× for 20 min and 1× for overnight at room temperature. The gels were stained with ethidium bromide (0.5 μg/mL) for 15 min, rinsed with distilled water, and visualized under UV light.

### 4.3. Affinity Chromatography on a Heparin Column

Protein purification of whole plant extracts was conducted using the ÄKTA Explorer System (Amersham Biosciences). Seven samples showing nucleolytic activity were run over a column filled with heparin (HiTrap Heparin column, 0.7·2.5 cm; GE Healthcare, cat. no. 17-0406-01). Heparin shows high affinity to DNA-binding proteins; hence, it is the best choice for affinity chromatography of nucleic acid binding proteins. Proteins with high affinity for heparin were bound to the resin, while the other components were carried through in buffer A and collected in “flow-through” fraction nos. 1–10. The proteins of interest were returned to solution in buffer B in fractions ~18–40. Absorbance was measured at 280 nm for all fractions, and chromatographs were plotted by UNICORN V3.0 software, Amersham Biosciences UK Limited, Little Chalfont, UK. For each run, 40 to 44 fractions were collected.

To isolate and purify proteins from the milky sap, approximately 0.5 μg protein was loaded onto a HiTrap heparin column equilibrated with 0.1 M Tris-HCl, pH 8.0, containing 10% glycerol. The column was eluted with a linear gradient of 0 to 2 M NaCl in the same buffer. The absorbance at 280 nm and DNase activity of all fractions (volume 1 mL) were determined.

### 4.4. Identification of Proteins Using LC-ESI-MS/MS

Stained protein bands were analyzed by liquid chromatography coupled to LTQ Orbitrap XL (Thermo Fisher Scientific, Waltham, MA, USA) in the Laboratory of Mass Spectrometry, Institute of Biochemistry and Biophysics, PAS, Warsaw, Poland. Excised gel fragments were placed in 1.5 mL Eppendorf tubes filled with 10% methanol and 2% acetic acid. The proteins were digested using trypsin. The generated peptides were concentrated, desalted on an RP-C18 precolumn (LC Packings, Coventry, UK), and further separated by UltiMate nano-HPLC (LC Packings, San Francisco, CA, USA). The column outlet was directly coupled to a Nanospray ion source operating in a data-dependent MS to MS/MS switch mode. Identification of proteins matching with the *C. majus* CDS database was performed (Cmajus 20150107_1; 209,790 sequences; 74,516,318 residues) [8,10] by using the MASCOT database search engine (Matrix Science, London, UK; www.matrixscience.com; accessed on 28 October 2021).

### 4.5. Identification of Nonprotein Substances by LC-ESI-MS/MS

Nonprotein substances (small molecules) associated with proteins showing nucleolytic activity were identified by LC-ESI-MS/MS in fractions separated by affinity chromatography on the heparin column.

#### 4.5.1. Sample Preparation 

Three hundred microliters of acetonitrile and 100 µL of 2 M Na_2_CO_3_ were added to 100 µL of samples. The samples were vortexed for 1 min and centrifuged for 5 min at 3500 rpm. Because of the high salt content in the collected fractions, acetonitrile did not mix with the aqueous phase, and the supernatant was directly transferred to a chromatographic vial.

#### 4.5.2. Extracts

LC-ESI-MS/MS consisted of an LC-20 chromatograph (Shimadzu, Duisburg, Germany) and a QTRAP 3200 mass spectrometer (ABSciex, Framingham, MA, USA). Separations were conducted using Luna 5u (C18/2) (4.6 · 250 mm; 5 µm) from Phenomenex (Torrance, CA, USA). Gradient elution was performed using 0.1% aqueous solution of HCOOH (phase A) and 0.1% acetonitrile solution of HCOOH (phase B). The % phase B was increased from 10% up to 90% at 30 min. Flow was set to 1.0 mL/min, the column was maintained at 30 °C, and the samples were kept at 20 °C. Next, 10 µL of the sample was injected into the column. The samples were screened using ESI (+) with the continuous MS scan mode in the first quadrupole (*m*/*z* 100–1000) and scan of product ions in the second quadrupole (*m*/*z* 100–1000). We used ESI (+) due to higher signal intensities than ESI (−). Parent ions for fragmentation were selected automatically by the software on the basis of the initial MS scan. Analyst ver. 1.4.2 (Sciex, Washington, DC, USA) was used as the data processing software. Measurements were performed at the Structural Research Laboratory of the Chemistry Department, University of Warsaw, Poland.

#### 4.5.3. Milky Sap 

LC-ESI-MS/MS consisted of an Alliance 2695 chromatograph coupled with a Quattro Micro mass spectrometer (Waters, Milford, CT, USA). Gradient elution was performed using a C18 Zorbax column (3.0·150 mm; 3.0 µm) (Agilent Technologies, Santa Clara, CA, USA). The mobile phase consisted of 0.1% HCOOH solution in 9:1 (*v*/*v*) H2O/acetonitrile mixture (phase A) and 0.1% HCOOH solution in 1:9 (*v*/*v*) H2O/acetonitrile mixture (phase B). The % phase B was increased from 0% up to 100% at 50 min. Flow was set to 0.3 mL/min, the column was maintained at 30 °C, and the samples were kept at 20 °C. Next, 10 µL of the sample was injected into the column. The samples were screened using ESI (+) with Single Ion Monitoring (SIM) and a continuous single quadrupole mass scan (*m*/*z* 300–400). Twenty-eight values of *m*/*z* were monitored based on signals observed in preliminary experiments and the expected analytes. Each value monitored with SIM was microscanned for ±0.1 unit. Additional measurements were performed for each sample with MS scan (*m*/*z* 200–400). Data acquisition and processing were performed using MassLynx ver. 4.1 software, Waters Corporation, Milford, MA, USA. The measurements were performed at the Pharmacology Department, Pharmaceutical Research Institute, Warsaw, Poland. A total of 21 signals were selected for indirect comparison on the basis of a peak area’s ratio of a given substance to the reference signal. A signal with intensity at *m*/*z* 342.2 was used as a reference (no. 21; 100% relative signal intensity).

### 4.6. Cell Lines

An HPV-positive human epithelial cancer cell line (HeLa) and an HPV-negative human epithelial cancer cell line (C33A) were obtained from American Type Culture Collection (ATCC, Manassas, VA, USA). The normal human fibroblast cell line (MSU-1.1) was provided by Prof. C. Kieda (CBM, CNRS, Orleans, France). All cell lines were grown in Dulbecco’s Modified Eagle’s Medium (DMEM) with high glucose, GlutaMAX™, and sodium pyruvate (Gibco, Life Technologies). The medium was supplemented with 10% (*v*/*v*) fetal bovine serum (Sigma-Aldrich) and 1% (*v*/*v*) antibiotics (penicillin and streptomycin, Sigma-Aldrich). All cells were cultivated as a monolayer on sterile culture plates (Sarstedt) at 37 °C in an atmosphere of 95% air and 5% CO_2_. When the cell culture reached a confluence of almost 80%, the medium was drained from the cell culture, and the adherent cells were washed with phosphate buffered saline (PBS, Sigma-Aldrich) and trypsinized with 0.25% Trypsin-EDTA (Sigma-Aldrich). After detachment of the cells, fresh medium was added to the culture, and the cells were counted by a TC10 Automated Cell Counter (Bio-Rad) and used to seed onto sterile tissue plates (Sarstedt).

### 4.7. Cell Viability and Cytotoxicity Test

To assess cell viability, the WST-1 colorimetric assay (Premixed WST-1 Cell Proliferation Reagent, Clontech) was used. The assay is based on the reduction of tetrazolium WST-1 salt (2-(4-iodophenyl)-3-(4-nitrophenyl)-5-(2,4-disulfophenyl)-2H-tetrazolium) to a soluble formazan by metabolically active cells, the concentration of which is directly proportional to the number of viable cells. HeLa, C33A, and normal fibroblasts (MSU-1.1) were seeded onto sterile 96-well tissue culture microplates (Sarstedt) at the concentration of 0.6 × 104 HeLa cells/well, 1.5 × 104 C33A cells/well, and 1 × 104 MSU-1.1 cells/well and incubated overnight in an incubator under standard conditions (at 37 °C and 5 wt% CO_2_). The stale medium was removed, cells were gently washed with PBS, and fresh medium with 20 μL of each fraction (protein concentration at 1–2 ng/µL) was added to selected wells to achieve a final volume of 100 µL/well. Untreated cells were used as negative control. Cells treated with 10% solution of DMSO (Sigma-Aldrich) were used as positive control. Next, plates were incubated at 37 °C and 5 wt% CO_2_ for 48 h. Then, 10 μL of WST-1 was added to each well and incubated for additional 2 h at 37 °C. The absorbance, which is proportional to cell viability, was measured by a spectrophotometer (Biochrom Anthos Zenyth 340 Microplate Reader) at 450 nm. Each cytotoxicity experiment was repeated at least three times. Cell viability was calculated as follows: Absorbance of treated cells/Absorbance of untreated (control) cells · 100%. Both HeLa and C33A cell lines are derived from cervical cancer samples of women. HeLa cells are HPV-positive (HPV+), while C33A are HPV-negative (HPV−). Statistics. All experiments were performed thrice. Statistical significance was calculated using GraphPad Prism (version 9.0.0), software (GraphPad Software, San Diego, CA, USA) and an unpaired *t* test.

### 4.8. MLP Structure Prediction and 3D Modeling

Tertiary structure prediction and fold-recognition were performed using the GeneSilico MetaServer gateway [38]. The top-scoring fold-recognition alignments to the structures of the selected template were used as a starting point for homology modeling using “Frankenstein’s Monster” approach [39,40], which comprises cycles of model building, evaluation, realignment in poorly scored regions, and merging of the best scoring fragments. For model evaluation, two Model Quality Assessment Programs (MQAPs) were used: MetaMQAP [41] and PROQ [42]. MQAP scores can only predict the deviation of a model from the real structure (the actual deviation can be calculated only by comparison with the real structures, which are not available). Thus, the scores reported must be interpreted as estimates or predictions and not as ultimate validation of the model quality. However, both PROQ and MetaMQAP performed quite well in independent benchmarks and can be regarded as robust predictors. NCBI CDD [43] and GeneSilico MetaServer [38] predicted the conserved hydrophobic cavity for binding of small molecules in the structure of CmMLP1. The predicted quality of the mature protein structure is illustrated according to Cristobal et al. [44].

### 4.9. Molecular Docking

Alkaloid structures (ZINC01575028, dihydroberberine; ZINC03872044, 8-hydroxycheleritrine; ZINC03779067, berberine; ZINC20111233, protopine; ZINC01709414, coptisine; ZINC00000706, sanguinarine; ZINC20470298, stylopine; ZINC30727894, chelidonine) were retrieved from the ZINC database (http://zinc.docking.org/, accessed on 28 October 2021) and analyzed using Autodock Vina14 (The Scripps Research Institute, La Jolla, CA, USA) implemented in Chimera13 package. AutoDock Vina analysis was performed according to the strength of interaction between hydrophobic amino acids forming “pockets” in the MLP with the respective ligands and visualized by PyMOL (DeLano Scientific, San Carlos, CA, USA). For each of the 8 studied alkaloids, we analyzed 20 possible conformations of the ligand molecule at the active site of the protein. For the conformation with the lowest binding energy, interaction analysis with amino acid residues at the active site of the protein was performed by considering hydrogen bonds and ionic, hydrophobic, and π-electron interactions of aromatic rings (PoseView15) (structures retrieved from ZINC database). The conformational analysis was performed using the Autodock Vina program. It analyzes 20 possible conformations of the ligand molecule at the active site of the protein and calculates binding energy for each conformation.

## 5. Conclusions

To conclude, we combined biological chemistry and analytical and theoretical techniques to discover the molecular association between CmMLP1 and three alkaloids, namely dihydroberberine, 8-hydroxycheleritrine, and berberine, from *C. majus* milky sap. It was possible with the help of the developed toolbox and the proposed workflow (Figure 1), which may stimulate advanced research that links small molecular and macromolecular biology, botany, and pharmacognosy. The limitation of the work, which was the research using only one plant species, could be also its strength for future research. We isolated the MLP from *C. majus* latex and identified the accompanying low-molecular-weight compounds. We then analyzed their combined cytotoxic activities against cervical cancer cell lines. The cell viability was the lowest for HeLa HPV positive cells—with less than 10% of living cells—twice lower as for C33A HPV negative cells. A significant decrease in the viability of human cervical cancer cells may suggest the synergistic effect of CmMLP1 and alkaloids. Our results suggest conformation-related mechanism of their interaction; however, it still remains unknown and will be an interesting topic for further studies. Resolving these interactions will definitely help to elucidate the mechanism of anti-HPV activity of the *C. majus* latex. Another important topic concerns the function of the CmMLP1 for the plant’s latex and for overall plant physiology [34]. The proposed toolbox and workflow may therefore advance pharmacognosy, plant disease resistance research, and agricultural practices [45] to strengthen plant defense and to propose novel bioactive combinations of macro- and low-molecular compounds from different plant species with the advantage to modern pharmacology.

## Figures and Tables

**Table 1:** MS/MS results of protein bands cut from an in-gel DNase assay zymography gel (Figure 2, Band 1A) and a silver-stained SDS-PAGE gel (Figure 2, Band 1B) after electrophoretic separation of protein fractions isolated from whole plant extract. The main constituent of the fractions was MLP.

Accession Number	Protein Definition	Mascot Score	Mol. Mass (da)	Matched Peptides	Sequence Coverage (%)
Band 1A					
m.37898	m.37898 MLP-like protein 28	6205	39612	27	65.2
m.37901	m.37901 MLP-like protein 28	5490	41223	30	65.7
m.37897	m.37897 MLP-like protein 28	4549	22910	18	73.9
m.37895	m.37895 MLP-like protein 28	4202	22792	21	74.9
m.37902	m.37902 MLP-like protein 28	3868	22814	17	69.8
m.12632	m.12632 MLP-like protein 28	2459	26927	15	69.5
m.12634	m.12634 MLP-like protein 28	2334	21853	18	61.9
m.33033	m.33033 bifunctional epoxide hydrolase 2-like	1996	35790	17	59.9
m.37899	m.37899 MLP-like protein 28	1959	22825	18	56.6
uniq_06137	uniq_06137 14-3-3 protein	1309	29225	21	71.6
Band 1B					
m.60929	m.60929 ferredoxin--nadp leaf chloroplastic-like isoform 1	1978	40992	37	58.3
m.37898	m.37898 MLP-like protein 28	1951	39612	12	44.6
m.37901	m.37901 MLP-like protein 28	1656	41223	10	37.0
m.12632	m.12632 MLP-like protein 28	1190	26927	7	33.0
m.12634	m.12634 MLP-like protein 28	1137	21853	8	41.8
m.37897	m.37897 MLP-like protein 28	537	22910	7	42.7
m.37895	m.37895 MLP-like protein 28	267	22792	5	36.2
m.61102	m.61102 pectinesterase 3-like	218	63766	4	8.8
m.12630	m.12630 MLP-like protein 28	174	16874	2	17.7
m.60714	m.60714 glyceraldehyde-3-phosphate cytosolic-like	100	36972	1	4.1

**Table 2:** Molecular docking of alkaloids detected in *C. majus* milky sap fractions with nucleolytic activity to the 3D model of MLP. * Binding affinity below −5.00 kcal/mol is considered as high affinity. ** Binding affinity between −5.00 and −4.50 kcal/mol is considered as relatively high affinity.

No.	ZINC Database Number	Structure (Compound)	Binding Affinity (kcal/mol)	Docking Score
1	ZINC01575028	dihydroberberine	−5.98 *	−0.55
2	ZINC03872044	8-hydroxycheleritrine	−5.94 *	−0.50
3	ZINC03779067	berberine	−4.86 **	−0.37
4	ZINC20111233	protopine	−3.55	−1.09
5	ZINC01709414	coptisine	−3.45	−0.18
6	ZINC00000706	sanguinarine	−3.12	−0.80
7	ZINC20470298	stylopine	−2.84	−1.44
8	ZINC30727894	chelidonine	−2.14	−1.34

**Table 3:** Viability of C33A and HeLa cancer cell lines vs. normal MSU1.1 treated with latex fractions containing CmMLP1 accompanied with alkaloids.

Cell Line (CmMLP1 Concentration)	Fraction 24 (2 ng/µL)	Fraction 25 (1.5 ng/µL)	Fraction 26 (1 ng/µL)
MSU1.1	22.8%	32.8%	39.2%
C33A	16.0%	17.4%	14.8%
HeLa	9.1%	5.8%	5.9%

## Data Availability

Data is contained within the article or Supplementary Materials. The data presented in this study are available in “Supplementary Information” and “Supplementary Data Set” Files.

## Supplementary Materials

The following are available online at https://www.mdpi.com/article/10.3390/ijms222111838/s1.
