# Supplementary material for: Combined Protein and Alkaloid Research of Chelidonium majus Latex Reveals CmMLP1 Accompanied by Alkaloids with Cytotoxic Potential to Human Cervical Carcinoma Cells
Source: Int J Mol Sci. 2021 Oct 31;22(21):11838. doi: 10.3390/ijms222111838 (PMC8584587; doi:10.3390/ijms222111838)
Supplement: Supplementary file 1 [file ijms-22-11838-s001.zip › NawrotR_et_al_Supplementary_infromation.pdf]

Supplementary Information for

Combined Protein and Alkaloid Research of *Chelidonium majus* Latex Reveals CmMLP1 Accompanied by Alkaloids with Cytotoxic Potential to Human Cervical Carcinoma Cells

Robert Nawrot*, Alicja Warowicka, Piotr Józef Rudzki, Oskar Musidlak, Katarzyna Magdalena Dolata, Jacek Musijowski, Elżbieta Urszula Stolarczyk, Anna Goździcka-Józefiak

*correspondence to: rnawrot@amu.edu.pl

This PDF file includes:

Figures S1 to S52

Tables S1 to S13

Table S1. MS/MS results of protein band 1A identification cut out from in-gel DN-ase assay zymography gel after electrophoretic separation of protein fractions isolated from *Chelidonium majus* whole plant extract. *The full list of identified peptides is available in Data Set S1.*

No.	Accession number	Protein definition	Mascot score	Mol. mass (Da)	Matched peptides	Sequence coverage (%)
1	m.37898	m.37898 mlp-like protein 28	6205	39612	27	65.2
2	m.37901	m.37901 mlp-like protein 28	5490	41223	30	65.7
3	m.37897	m.37897 mlp-like protein 28	4549	22910	18	73.9
4	m.37895	m.37895 mlp-like protein 28	4202	22792	21	74.9
5	m.37902	m.37902 mlp-like protein 28	3868	22814	17	69.8
6	m.12632	m.12632 mlp-like protein 28	2459	26927	15	69.5
7	m.12634	m.12634 mlp-like protein 28	2334	21853	18	61.9
8	m.33033	m.33033 bifunctional epoxide hydrolase 2-like	1996	35790	17	59.9
9	m.37899	m.37899 mlp-like protein 28	1959	22825	18	56.6
10	uniq_06137	uniq_06137 14-3-3 protein	1309	29225	21	71.6
11	m.15103	m.15103 late embryogenesis abundant protein lea14-	1110	22400	8	59
12	m.60893	m.60893 polyphenol oxidase	1075	66998	14	33.3
13	m.61093	m.61093 2-dehydro-3-deoxyphosphooctonate aldolase 1	998	32440	13	55.4
14	m.2553	m.2553 14-3-3-like protein d-like	993	27462	11	38.5
15	m.14064	m.14064 protein in2-1 homolog b-like	978	33990	15	53.8
16	m.60779	m.60779 elongation factor 1-delta-like	964	24838	12	64.5
17	m.12630	m.12630 mlp-like protein 28	936	16874	8	65.3
18	m.14066	m.14066 protein in2-1 homolog b-like	918	26722	12	57.9
19	uniq_04418	uniq_04418 14-3-3-like protein d-like	915	25457	13	48.6
20	m.60799	m.60799 lactoylglutathione lyase-like	893	38719	14	40.2
21	m.60746	m.60746 annexin d2-like	842	36136	11	40.4
22	m.11279	m.11279 14-3-3 protein	832	13277	7	68.1

Table S2. MS/MS results of protein band 1B identification cut out from silver stained SDS-PAGE gel after electrophoretic separation of protein fractions isolated from *Chelidonium majus* whole plant extract. *The full list of identified peptides is available in Data Set S2.*

No.	Accession number	Protein definition	Mascot score	Mol. mass (Da)	Matched peptides	Sequence coverage (%)
1	m.60929	m.60929 ferredoxin--nadp leaf chloroplastic-like isoform 1	1978	40992	37	58.3
2	m.37898	m.37898 mlp-like protein 28	1951	39612	12	44.6
3	m.37901	m.37901 mlp-like protein 28	1656	41223	10	37
4	m.12632	m.12632 mlp-like protein 28	1190	26927	7	33
5	m.12634	m.12634 mlp-like protein 28	1137	21853	8	41.8
6	m.37897	m.37897 mlp-like protein 28	537	22910	7	42.7
7	m.37895	m.37895 mlp-like protein 28	267	22792	5	36.2
8	m.61102	m.61102 pectinesterase 3-like	218	63766	4	8.8
9	m.12630	m.12630 mlp-like protein 28	174	16874	2	17.7
10	m.60714	m.60714 glyceraldehyde-3-phosphate cytosolic-like	100	36972	1	4.1

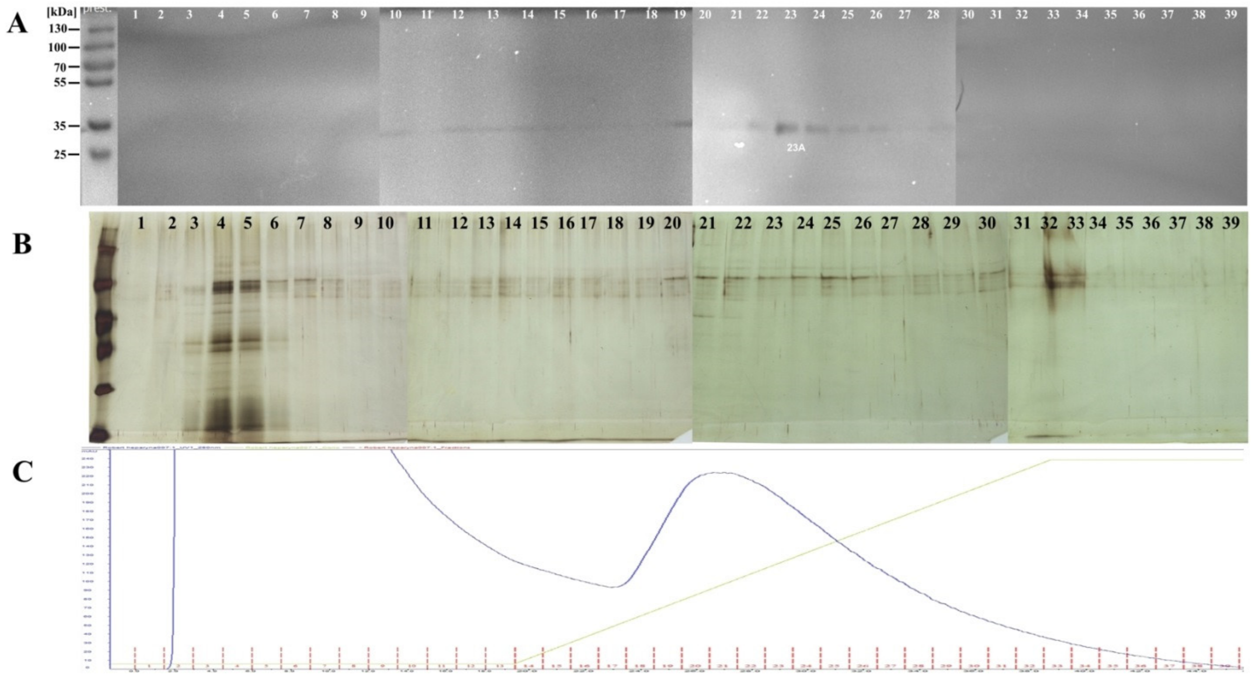

Figure S1. Fractionation of *C. majus* latex on heparin column using HPLC. (A) In-gel DNase zymography gel of protein fractions isolated from *C. majus* latex with high nucleolytic activity. The band of ca. 35 kDa MW with nucleolytic activity was cut and subjected to MS identification. (B) SDS-PAGE stained with silver represents the protein profile of each fraction. The method was not sensitive enough to clearly show the presence of respective band of ca. 35 kDa. (C) Heparin affinity chromatography fractionation of *C. majus* latex. Blue line indicates absorbance at 280 nm correlated with protein content in fractions. Red lines and numbers represent fraction numbers.

Table S3. MS/MS results from protein band 23A cut from In-gel DNase zymography gel of protein fractions isolated from *C.majus* latex (Figure S1A). One of the proteins identified was MLP protein. The fraction contained small amount of the protein, so the MLP confirmation is not as strong as for fractions from *C. majus* whole plant extract (Manuscript, Figure 1). *The full list of identified peptides is available upon request.*

**Accession number**	**Protein definition**	**Mascot score**	**Mol. mass (Da)**	**Matched peptides**	**Sequence coverage (%)**
m.60799	lactoylglutathione lyase-like	380	38719	6	19.5
m.36568	glucan endo- -beta-glucosidase gvi-like	157	39752	3	12.2
m.60713	lipoxygenase homology domain-containing protein 1-like	145	19551	4	14.9
m.12634	**mlp-like protein 28**	136	21853	4	24.9
m.2920	31 kda chloroplastic-like	106	35872	2	9.2
m.37895	**mlp-like protein 28**	87	22792	2	8.5
m.7903	uncharacterized_protein	81	16560	2	6.0

Figures S2-S21: Identification of nonprotein substances from *C. majus* whole plant extract fractions by LC-MS/MS

Nonprotein substances (small molecules) associated with proteins of nucleolytic activity were identified by LC-ESI-MS/MS in fractions separated by affinity chromatography on heparin column. Fractions from whole plant extracts were assayed in 25 samples.

1. Sample preparation

300 µL of acetonitrile and 100 µL of Na_2CO_3 2M were added to 100 µL of sample. Samples were vortexed mixed for 1 min and centrifuged for 5 min at 3,500 rpm. Due to high salt content in the collected fractions, acetonitrile did not mix with aqueous phase and supernatant was directly transferred to the chromatographic vial.

LC-ESI-MS/MS was carried out on LC-20 chromatograph (Shimadzu, Duisburg, Germany) and QTRAP 3200 mass spectrometer (ABSciex, Framingham, USA). Separations were conducted using Luna 5u (C18/2) (4.6x 250 mm; 5 µm) from Phenomenex (Torrance, CA, USA). Gradient elution was performed using: 0.1% aqueous solution of HCOOH (phase A) and 0.1% acetonitrile solution of HCOOH (phase B). The %B was increased from 10% up to 90% at 30 min. Flow was set to 1.0 mlmlml/min, the column was maintained at 30°C, whereas the samples were kept at 20°C. 10 µL of sample was injected onto the column.

Samples were screened using ESI (+) with continuous MS scan mode in the first quadrupole (100-1000 m/z) and scan of product ions in the second quadrupole (100-1000 m/z). Parent ions for fragmentation were selected automatically by the software on basis of the initial MS scan. Data processing software was Analyst ver. 1.4.2. Measurements were executed at the Structural Research Laboratory of the Chemistry Department, University of Warsaw, Poland.

2. Results

Applied generic chromatographic method allowed for a rough separation of extracted sample constituents. Signal observed at retention time of ca. 35 min was observed in a pure acetonitrile, thus it was considered as not sample-specific. However, taking advantage of the signal's occurrence in every chromatogram, its intensity at m/z 342.2 was used as a reference signal to correct for instrumental variability as well as sensitivity drift. For signals of sufficient intensity, fragmentation spectra in the m/z range of 100 up to the m/z value of molecular ion were collected. Due to the fragmentation parameters not optimized for each substance, only the most intense signals yielded useful results. A typical chromatogram of total ion current (TIC) obtained for samples from the first batch on QTrap 3200 instrument is presented in Supplementary **Error! Reference source not found.**2. Signals are marked with m/z values of detected ions.

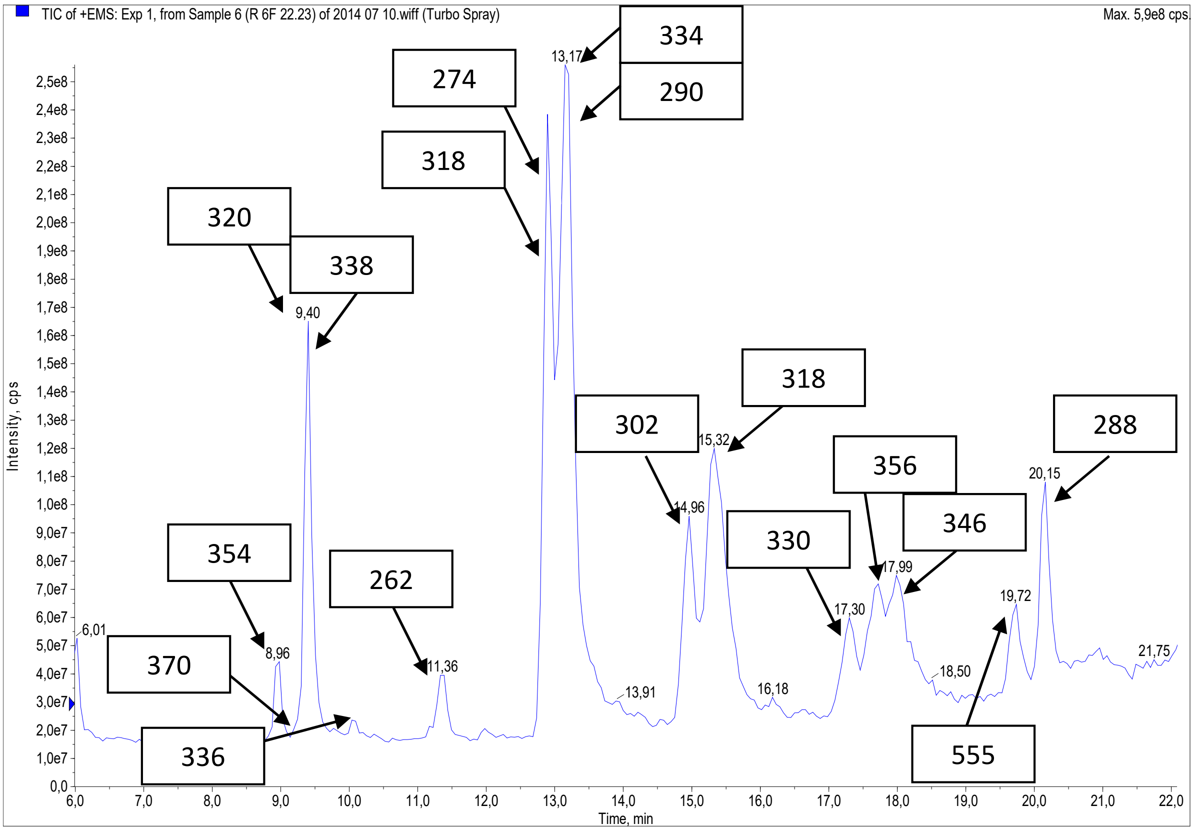

Figure S2. Chromatogram obtained on QTrap instrument for sample coded R6F 22.23 with m/z values of recorded ions

Some of the m/z values matched the values expected for molecular ions of previously identified substances in *C. majus*. The fragmentation spectra for these ions are given in Supplementary Figure S3 to Supplementary **Error! Reference source not found.**7. However 16 of the recorded fragmentation spectra at remaining m/z values could not be assigned to any of the previously reported substances. The fragmentation spectra for these ions are given from Supplementary Figure S8 to Supplementary Figure S21.

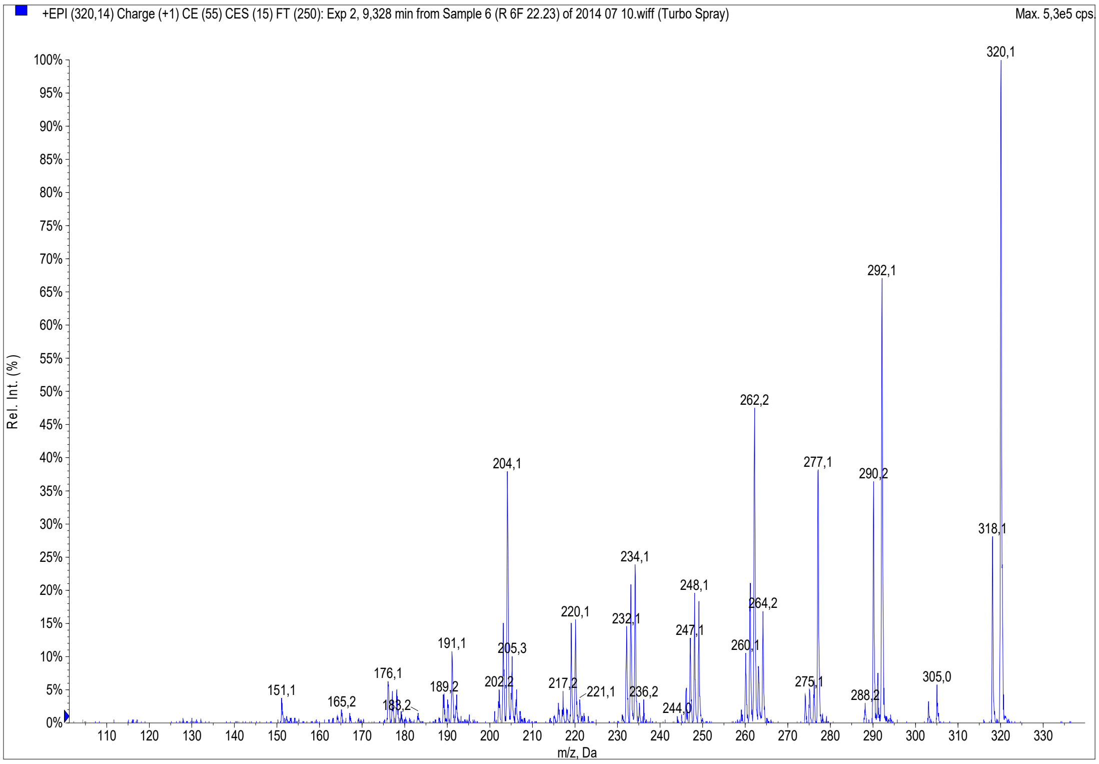

Figure S3. ESI (+) fragmentation spectrum of an ion at m/z = 320. Possible presence of norsanguinarine, dihydroxysanguinarine or coptisine.

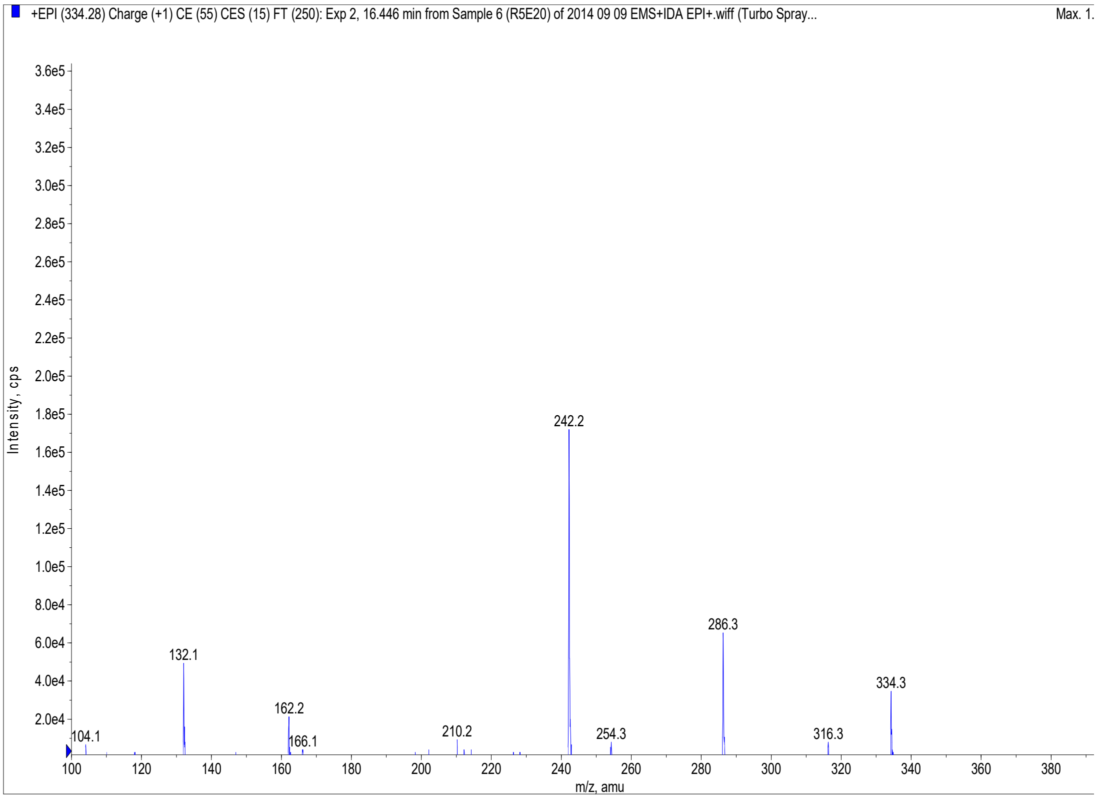

Figure S4. ESI (+) fragmentation spectrum of an ion at m/z = 334. Possible presence of norchelerythrine, dihydrosanguinarine or corysamine.

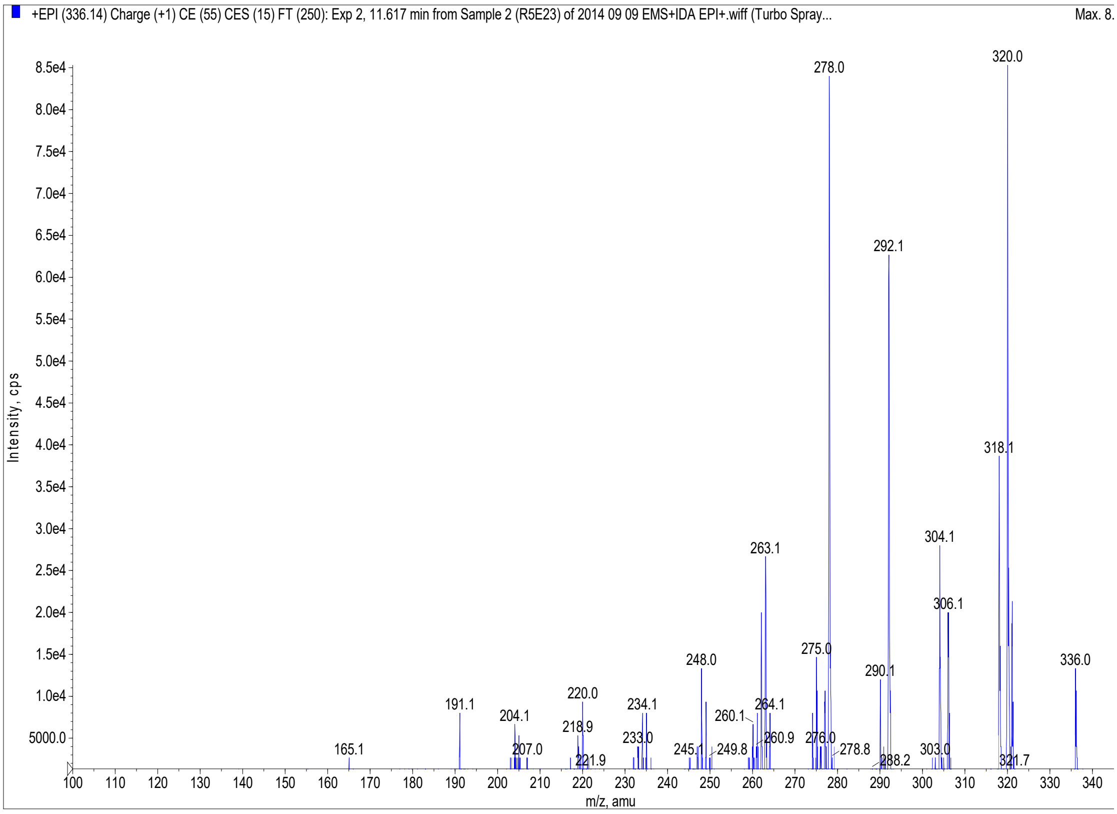

Figure S5. ESI (+) fragmentation spectrum of an ion at m/z = 336. Fragmentation pattern indicates possible presence of berberine or dihydroxychelerythrine.

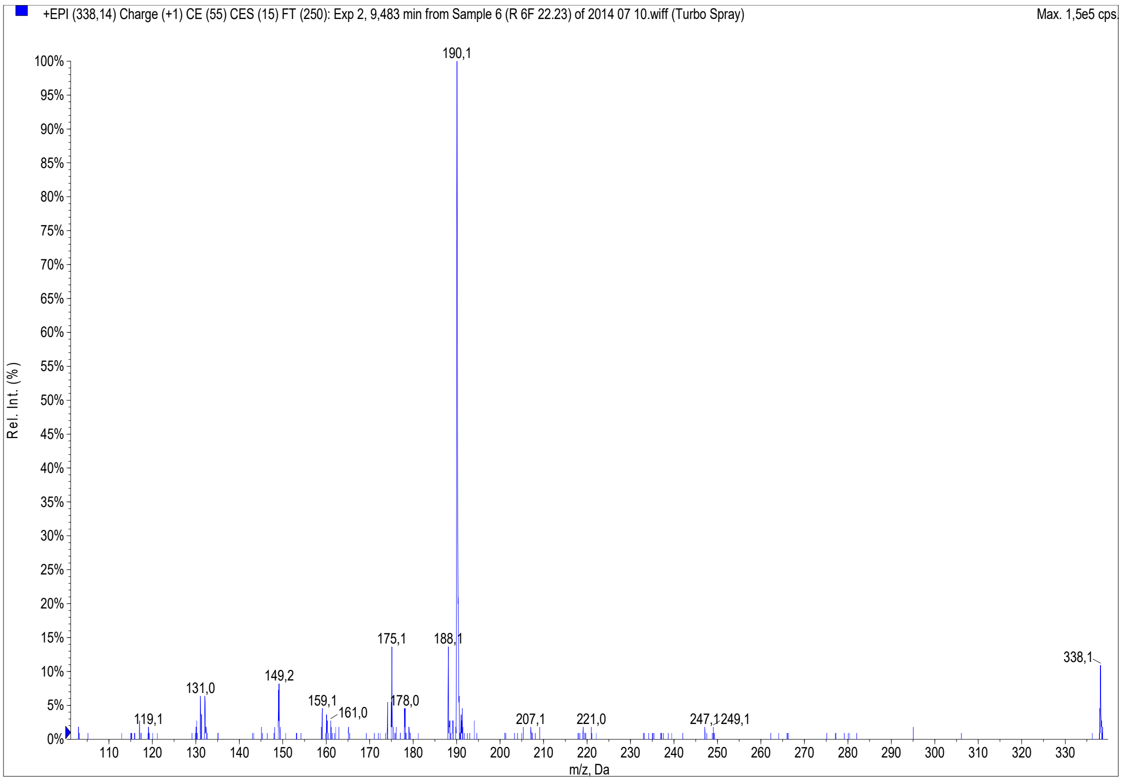

Figure S6. ESI (+) fragmentation spectrum of an ion at m/z = 338. Possible presence of dihydroberberine

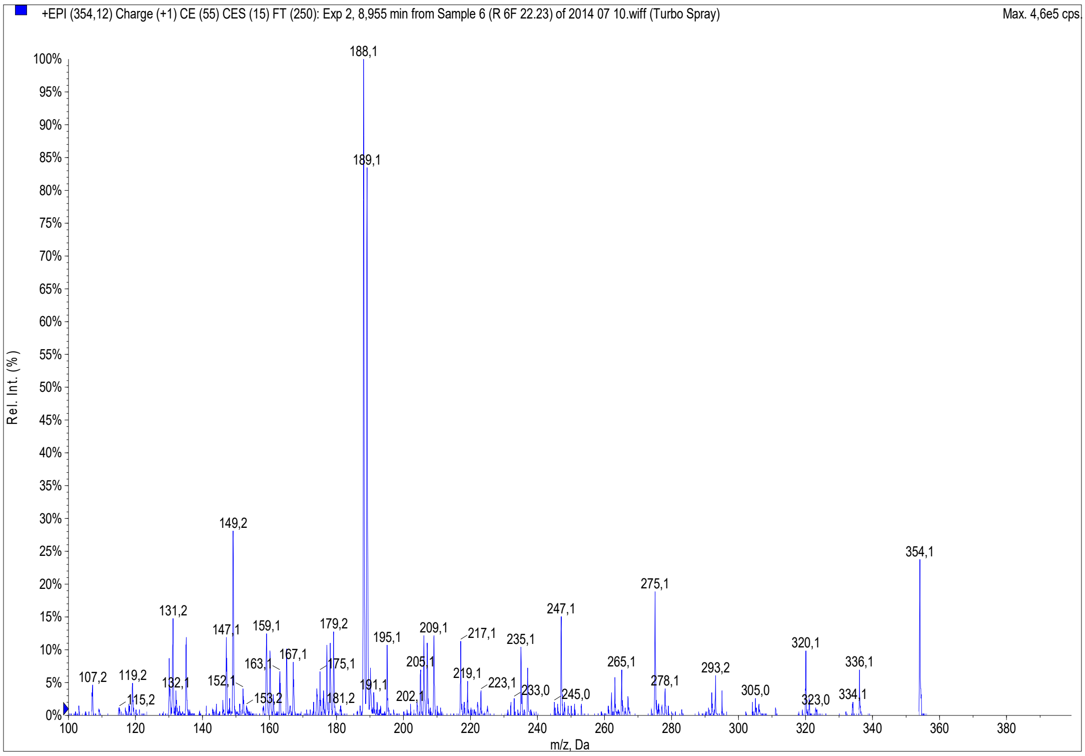

Figure S7. ESI (+) fragmentation spectrum of an ion at m/z = 354. Fragmentation pattern indicates possible presence of chelidonine or protopine

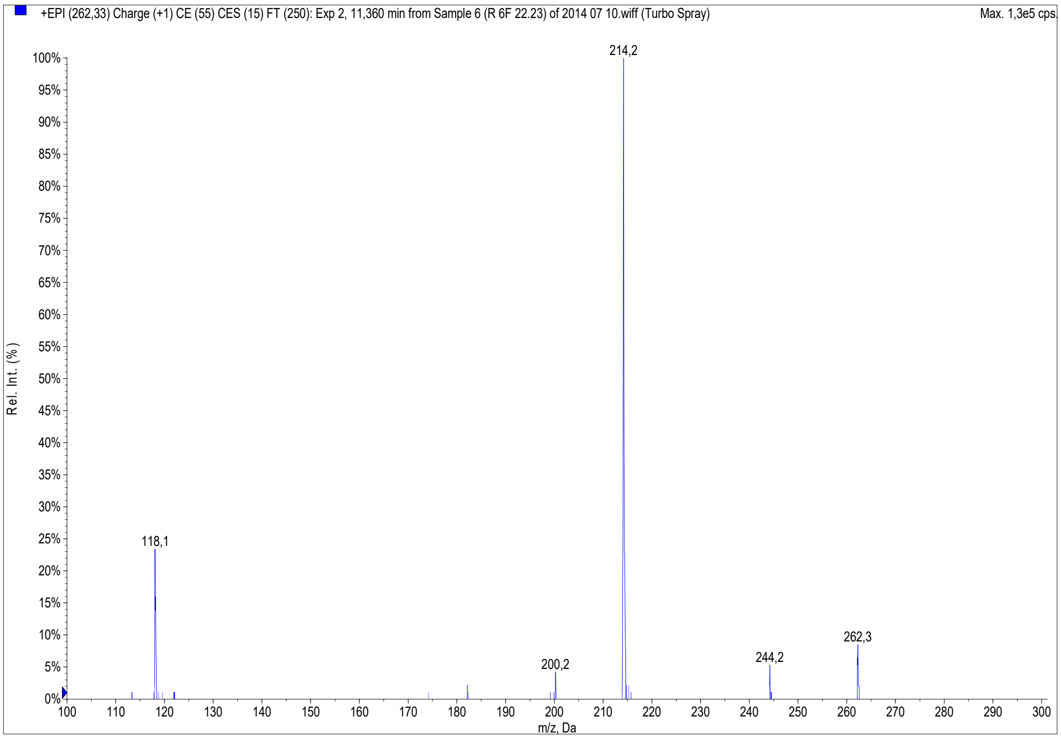

Figure S8. ESI (+) fragmentation spectrum of an ion at m/z = 262. Unknown constituent.

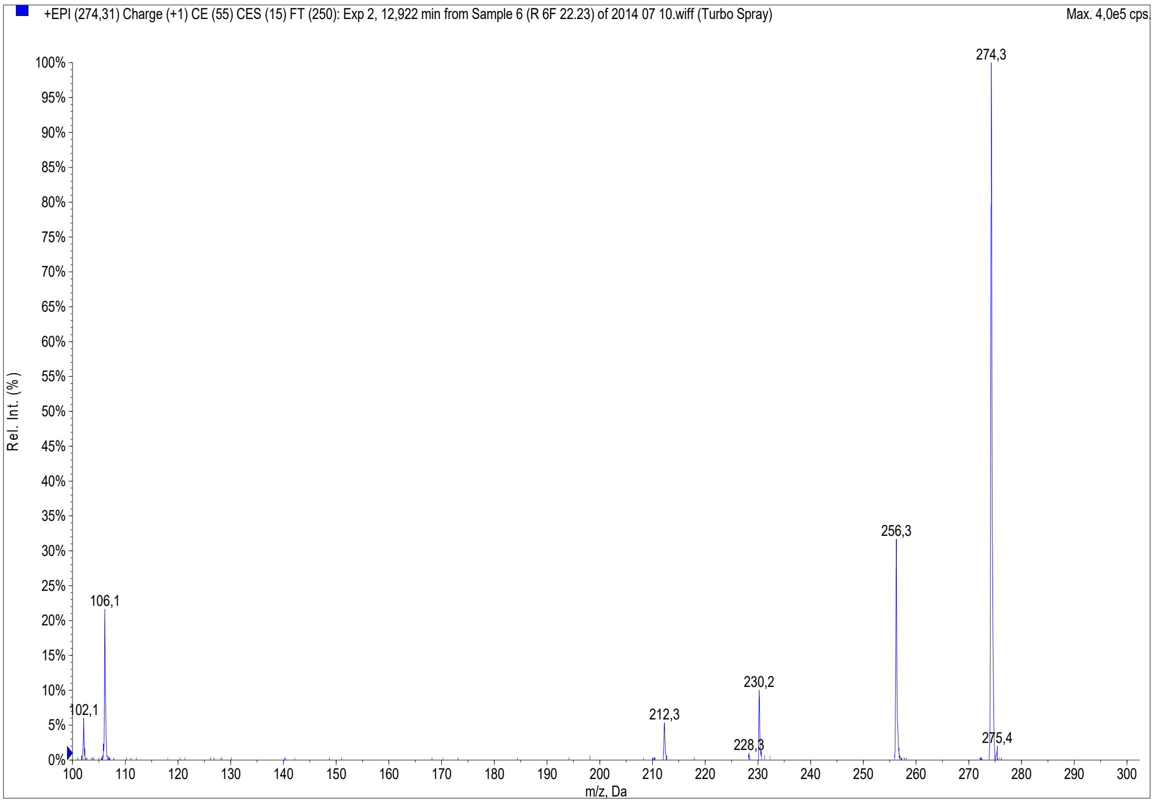

Figure S9. ESI (+) fragmentation spectrum of an ion at m/z = 274. Unknown constituent.

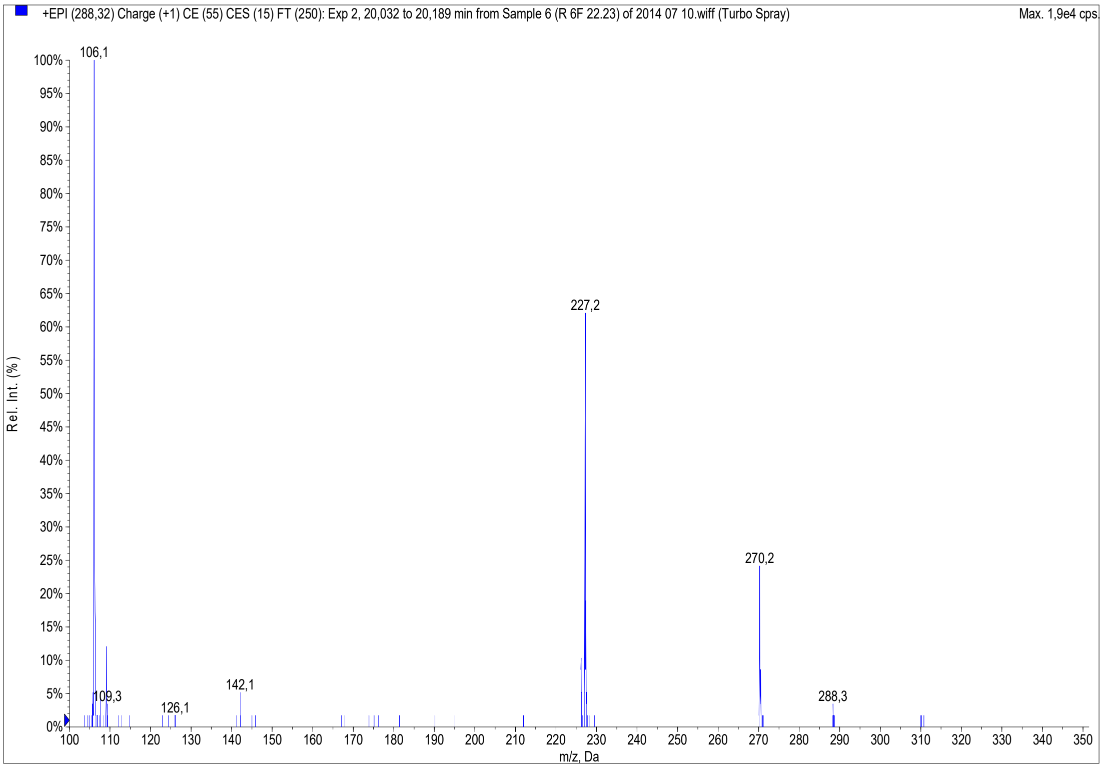

Figure S10. ESI (+) fragmentation spectrum of an ion at m/z = 288. Unknown constituent.

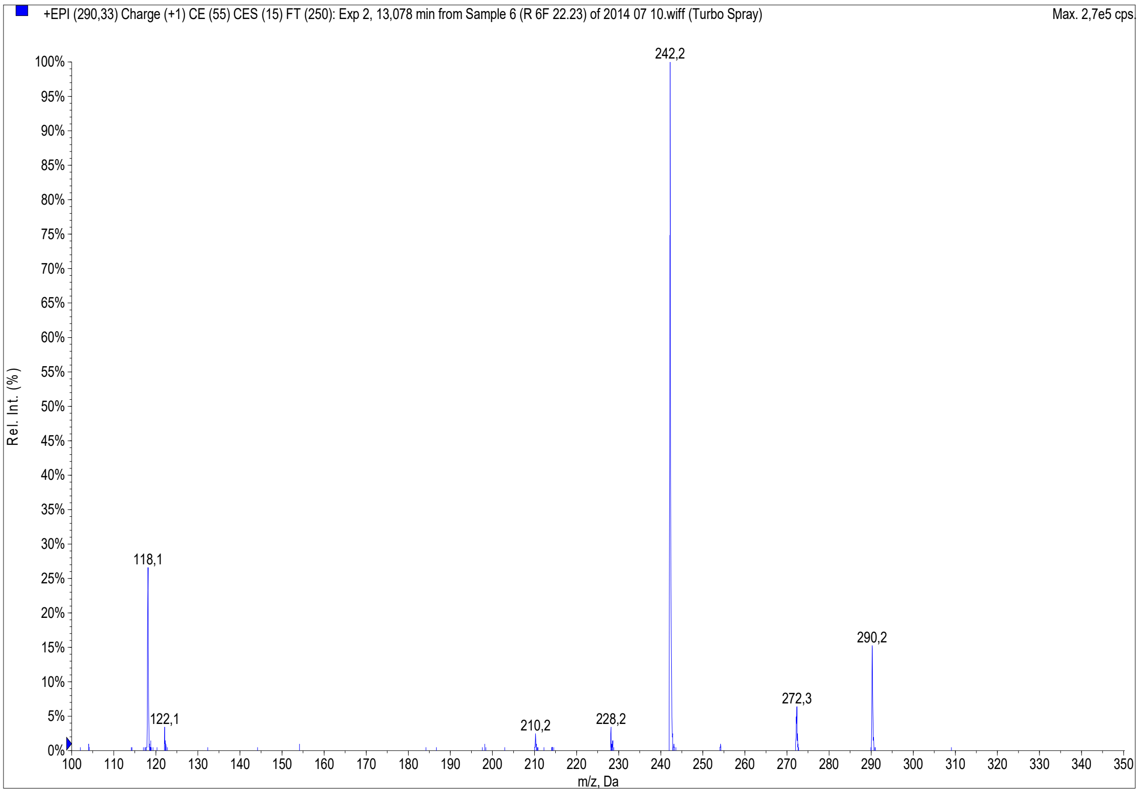

Figure S11. ESI (+) fragmentation spectrum of an ion at m/z = 290. Unknown constituent.

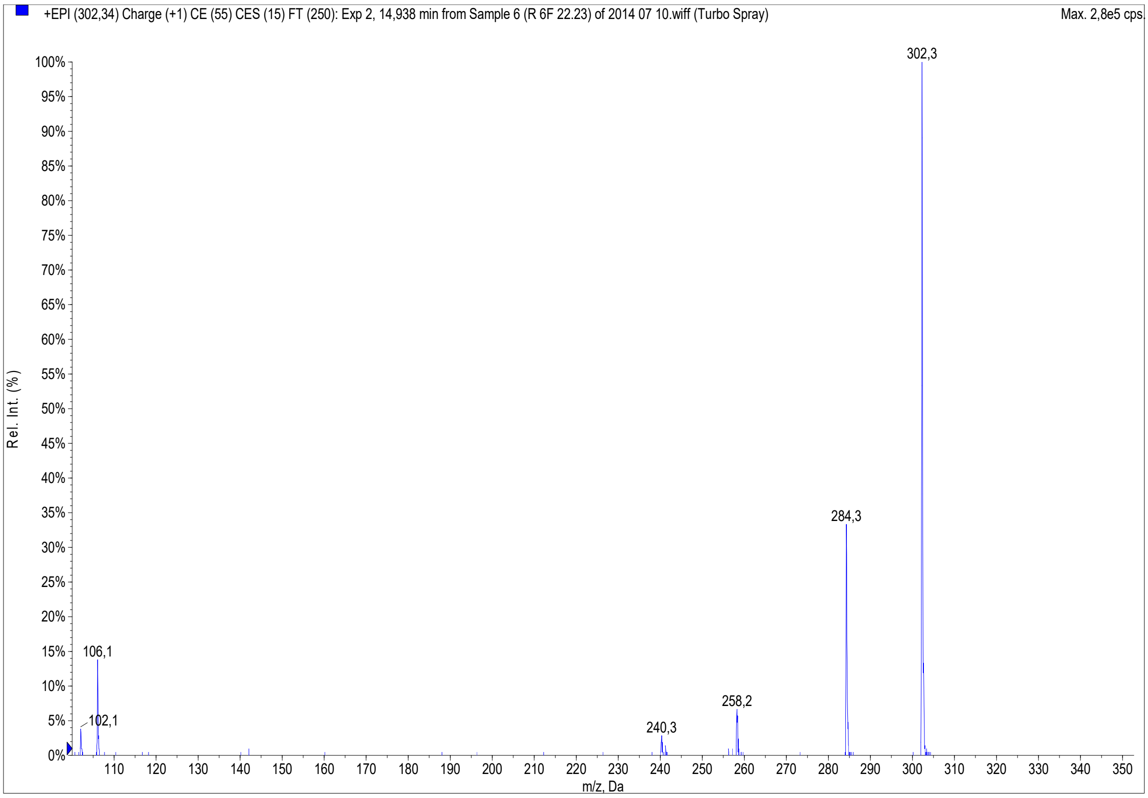

Figure S12. ESI (+) fragmentation spectrum of an ion at m/z = 302. Unknown constituent.

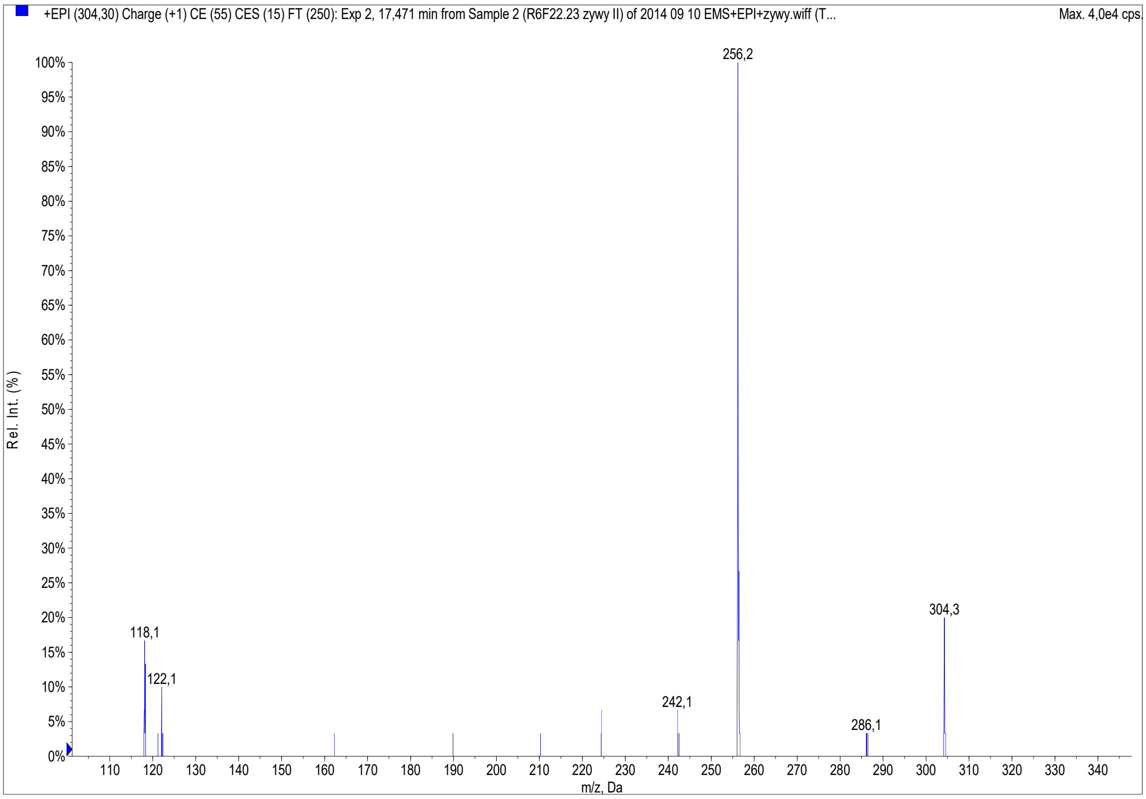

Figure S13. ESI (+) fragmentation spectrum of an ion at m/z = 304. Unknown constituent.

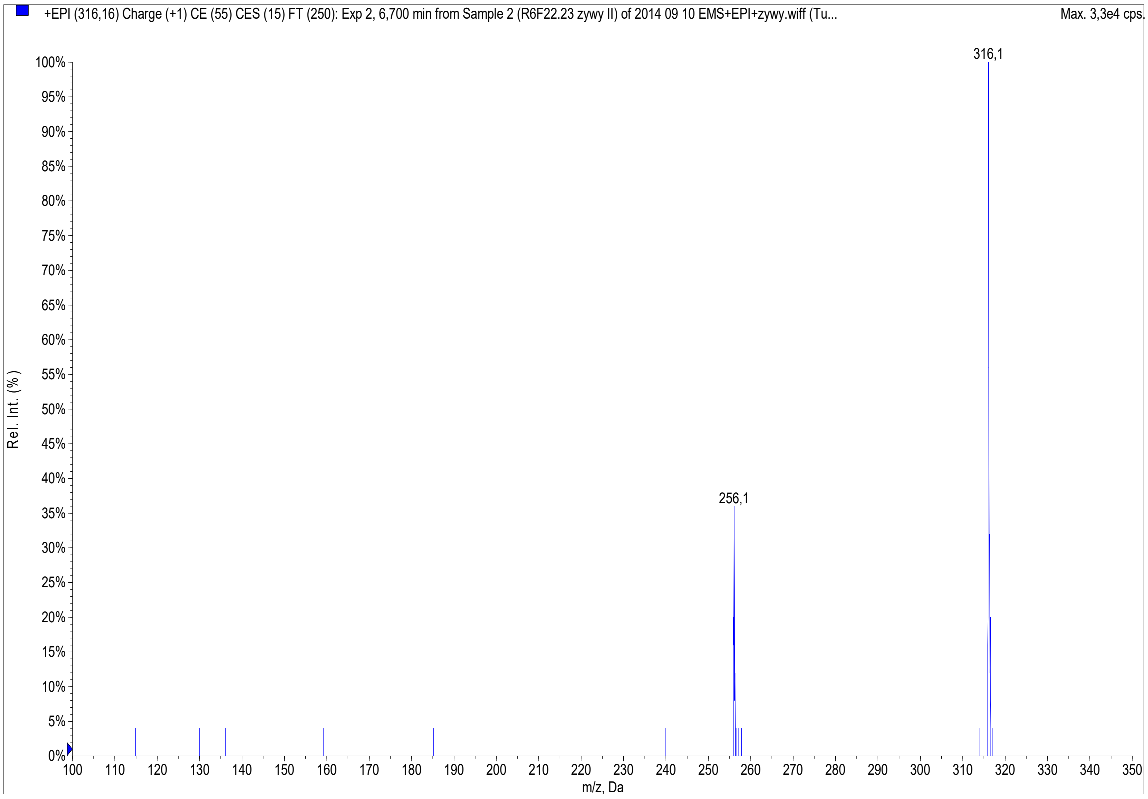

Figure S14. ESI (+) fragmentation spectrum of an ion at m/z = 316. Unknown constituent.

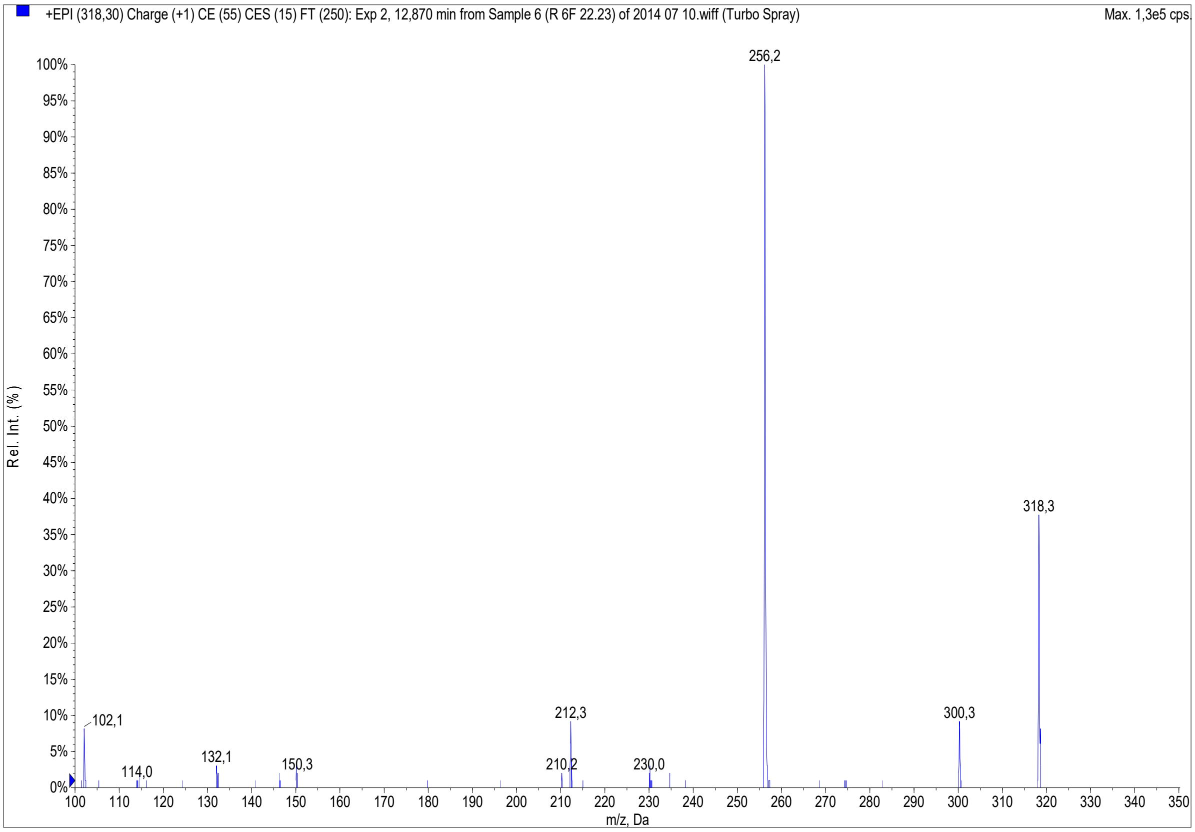

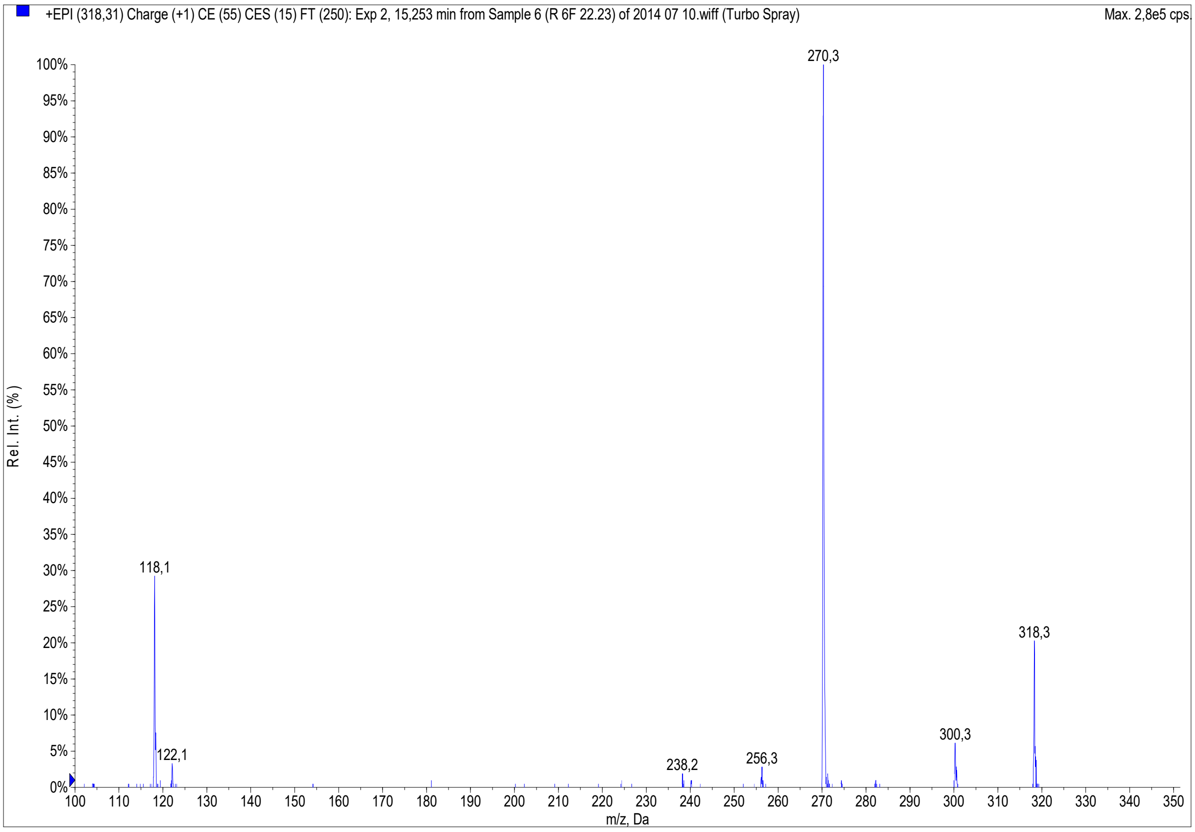

Figure S15. ESI (+) fragmentation spectra of an ion at m/z = 318. Unknown constituents.

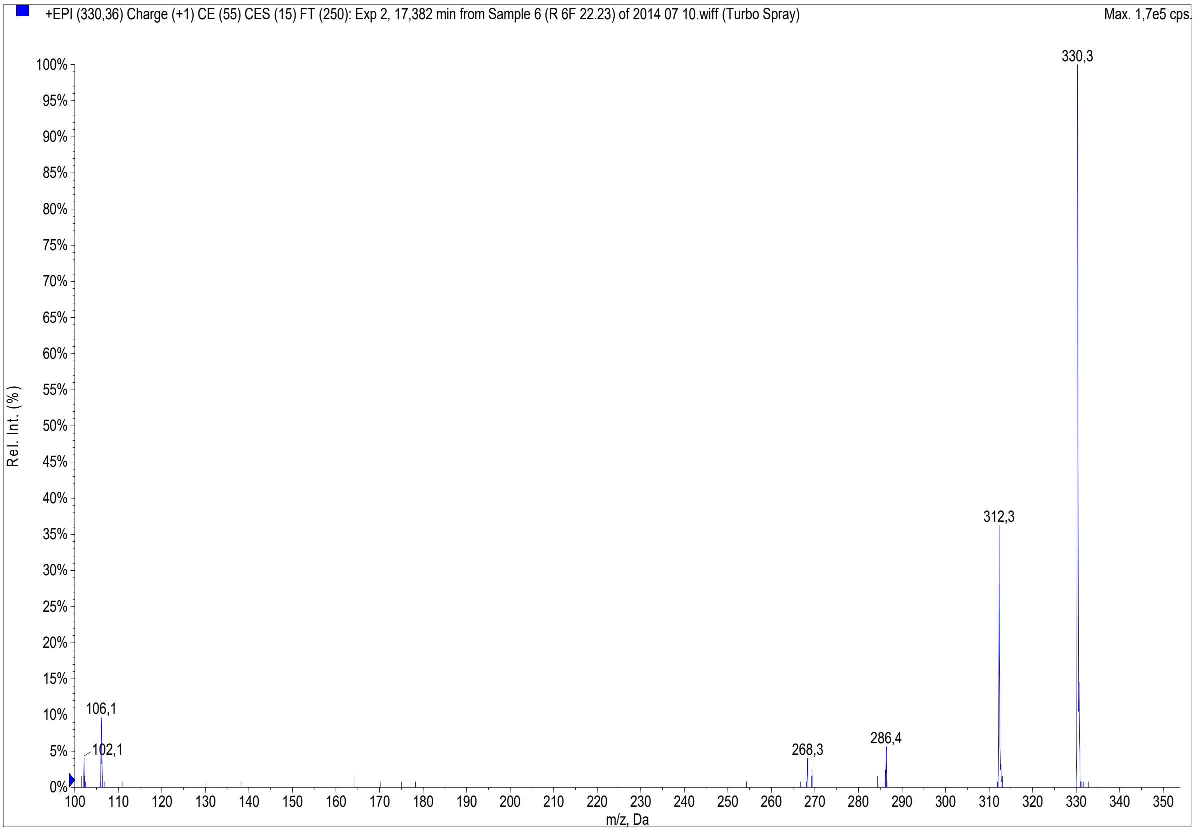

Figure S16. ESI (+) fragmentation spectrum of an ion at m/z = 330. Unknown constituent.

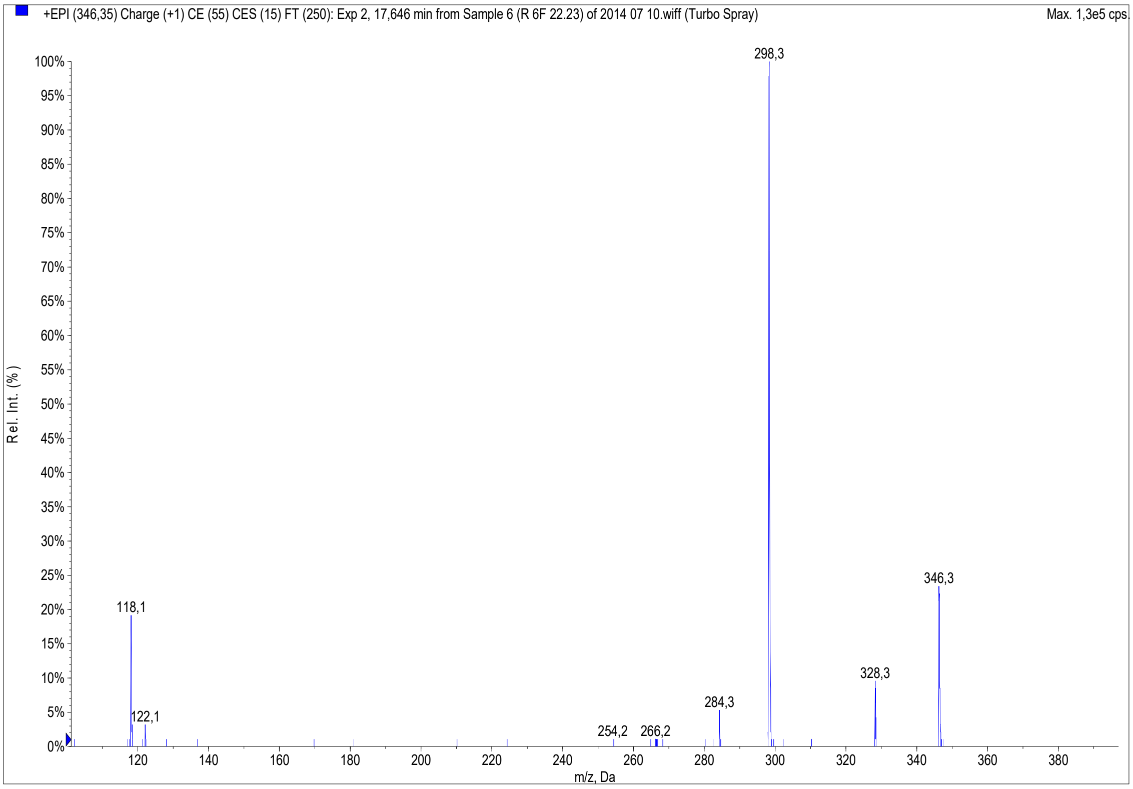

Figure S17. ESI (+) fragmentation spectrum of an ion at m/z = 346. Unknown constituent.

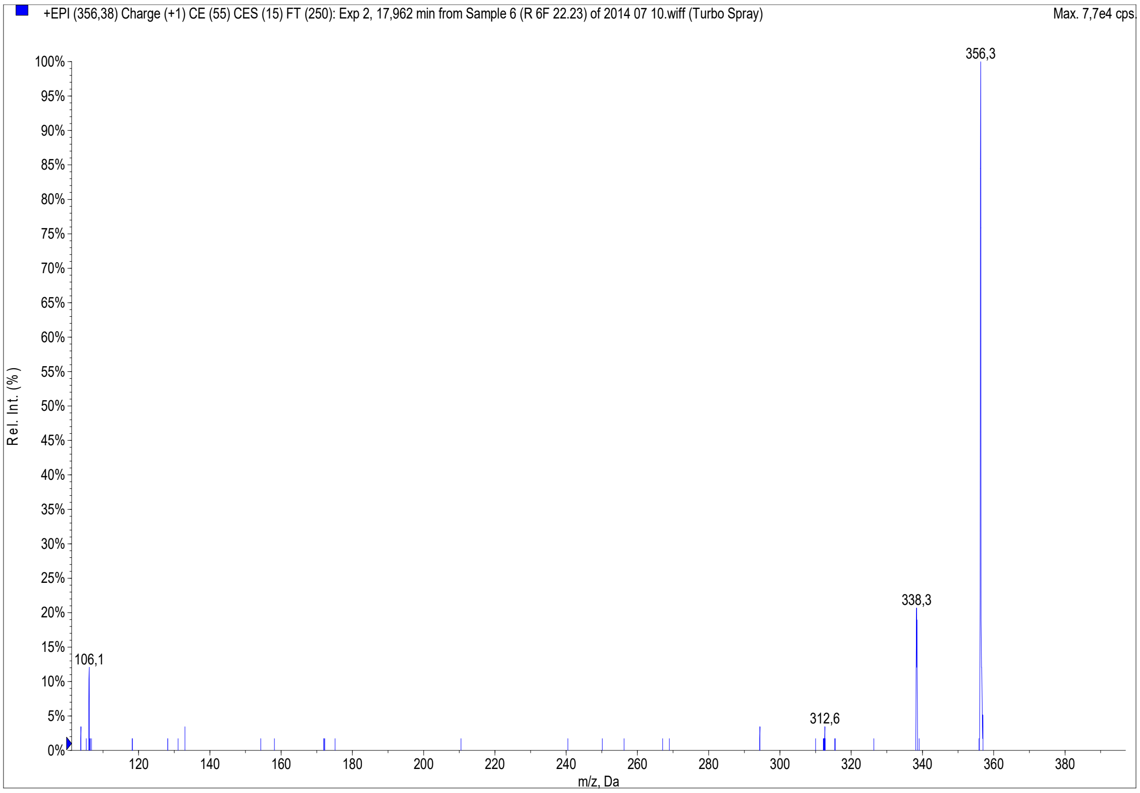

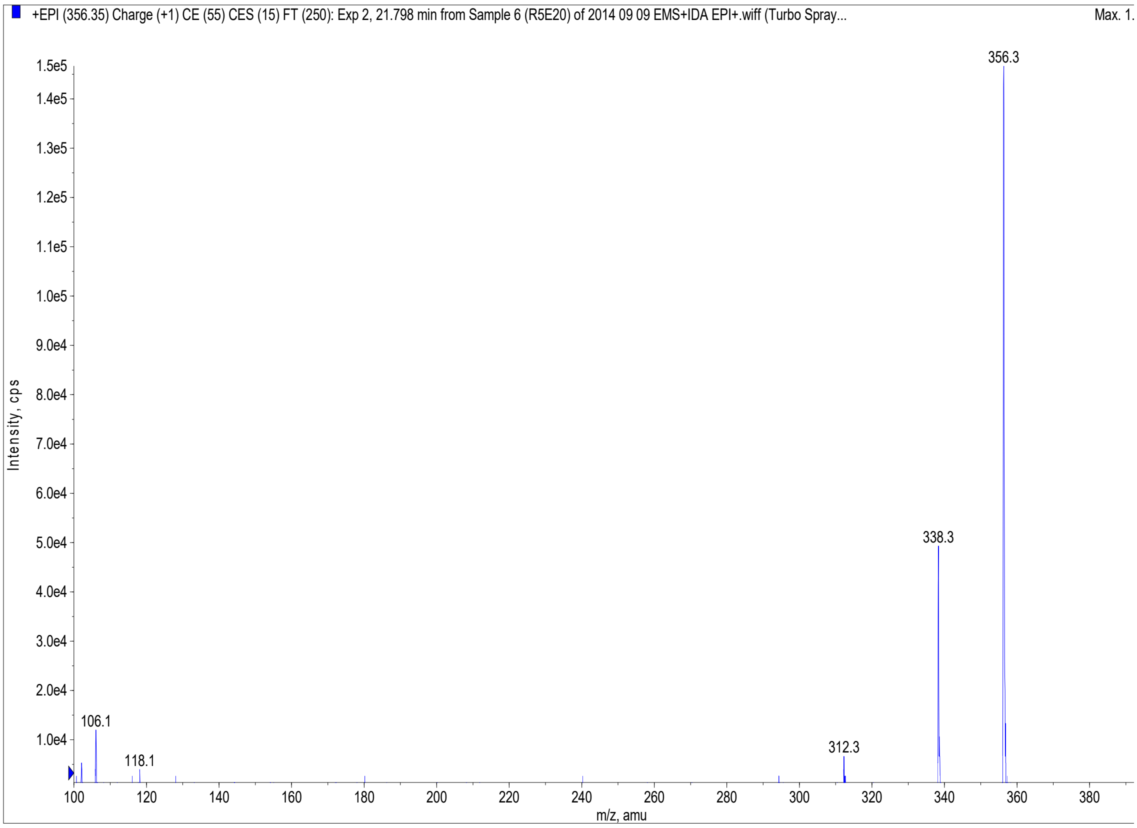

Figure S18. ESI (+) fragmentation spectra of an ion at m/z = 356. Unknown constituents.

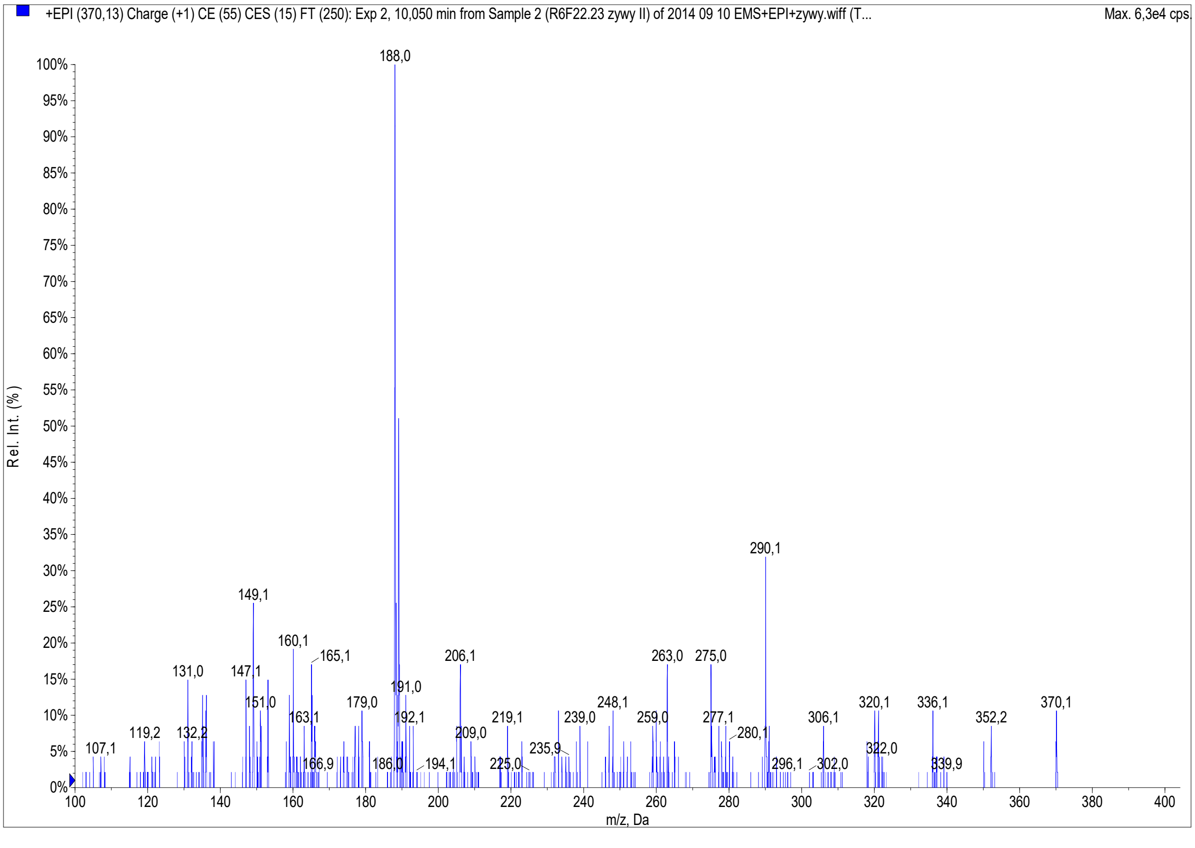

Figure S19. ESI (+) fragmentation spectrum of an ion at m/z = 370. Unknown constituent.

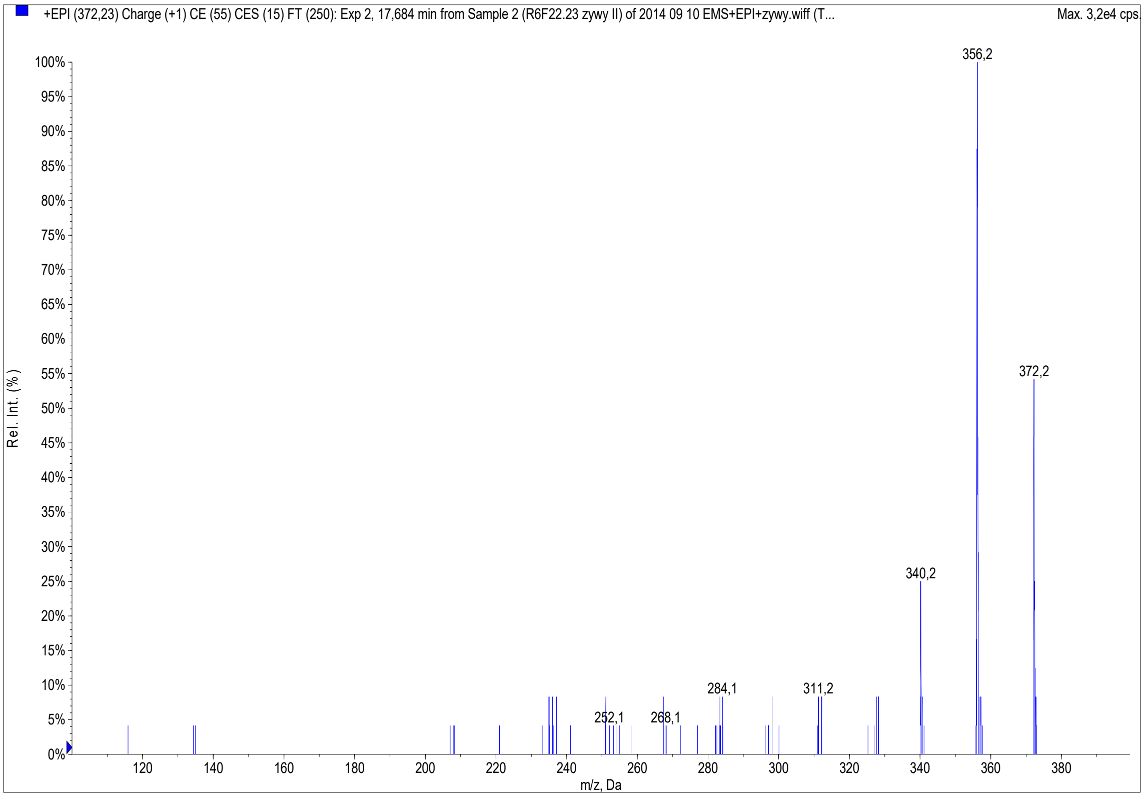

Figure S20. ESI (+) fragmentation spectrum of an ion at m/z = 372. Unknown constituent.

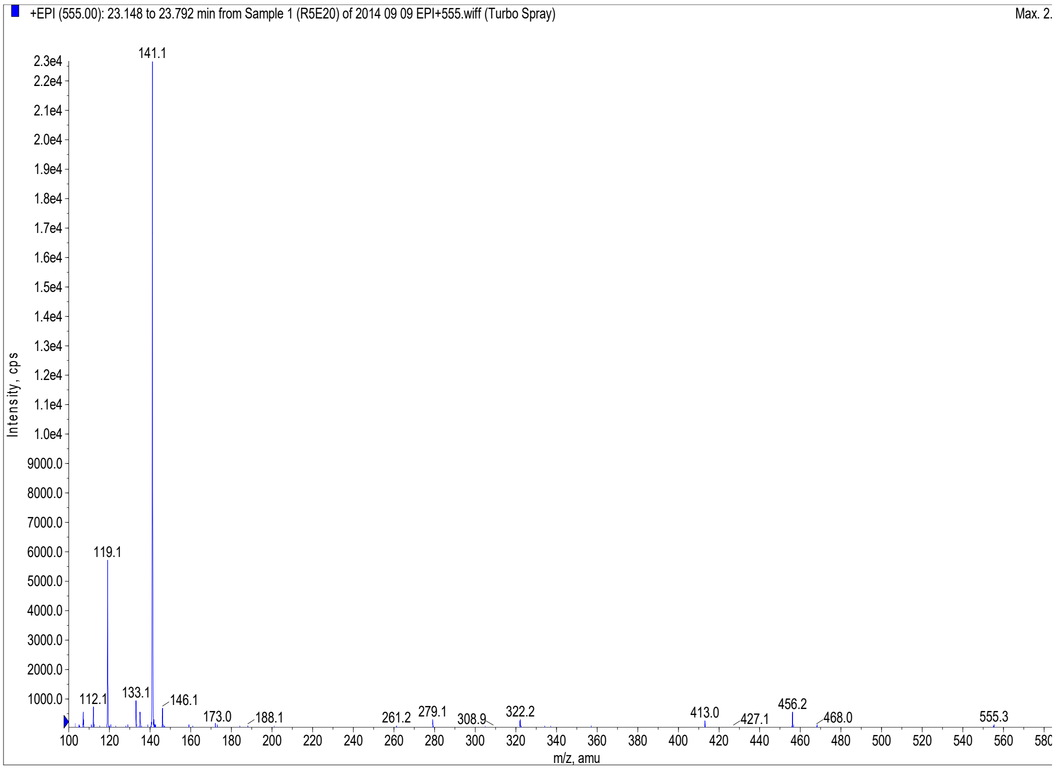

Figure S21. ESI (+) fragmentation spectrum of an ion at m/z = 555. Unknown constituent.

Figures S22 – S33: Identification of nonprotein substances from *C. majus* latex fractions by LC-MS/MS

Nonprotein substances (small molecules) associated with proteins of nucleolytic activity were identified by LC-ESI-MS/MS in fractions separated by affinity chromatography on heparin column. Fractions from whole plant extracts were assayed in 82 samples.

1. Sample preparation

300 μL of acetonitrile and 100 μL of Na_2CO_3 2M were added to 100 μL of sample. Samples were vortexed mixed for 1 min and centrifuged for 5 min at 3,500 rpm. Due to high salt content in the collected fractions, acetonitrile did not mix with aqueous phase and supernatant was directly transferred to the chromatographic vial.

LC-ESI-MS/MS was carried out on Alliance 2695 chromatograph coupled with Quattro Micro mass spectrometer (Waters, Milford, USA). Gradient elution was performed using: a C18 Zorbax column (3.0x 150 mm; 3.0 μm) (Agilent Technologies, Santa Clara, USA). A mobile phase consisted of 0.1% HCOOH solution in 9:1 (v/v) H_2O/acetonitrile mixture (phase A) and 0.1% HCOOH solution in 1:9 (v/v) H_2O/acetonitrile mixture (phase B). The %B was increased from 0% up to 100% at 50 min. Flow was set to 0.3 mlmlml/min, the column was maintained at 30°C, whereas the samples were kept at 20°C. 10 μL of sample was injected onto the column.
Samples were screened using ESI (+) with Single Ion Monitoring (SIM) and a continuous single quadrupole mass scan (300-400 m/z). 28 values of m/z were monitored, basing on signals observed in preliminary experiments and expected analytes (Table S1). Each value monitored with SIM was microscanned for ± 0.1 unit. Additional measurement was performed for each sample with MS scan (200-400 m/z).

Data acquisition and processing was performed using MassLynx ver. 4.1 software. The measurements were executed at the Pharmacology Department, Pharmaceutical Research Institute, Warsaw, Poland. 21 signals were selected for indirect comparison on basis of a peak area's ratio of given substance to the reference signal. Signal with intensity at m/z 342.2 was used as a reference (100% relative signal intensity).

2. Results

LC-MS/MS analyses of *C. majus* latex fractions - fragmentation spectra with proposed identification and fragments

Applied generic chromatographic method allowed for a rough separation of sample constituents. A typical chromatogram of total ion current (TIC) obtained on Quattro Micro instrument is presented in Figure S1.

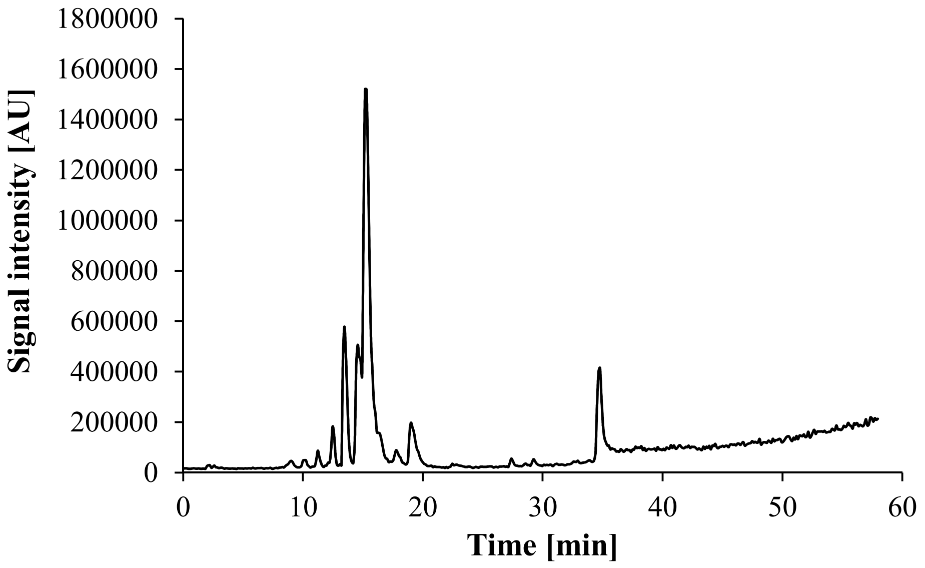

Figure S22. Chromatogram obtained on Quattro Micro instrument for sample coded FT 6.4

Signal observed at retention time of ~35 min was observed in a pure acetonitrile, therefore it was considered as not sample-specific. However, taking advantage of the signal's occurrence in every chromatogram, its intensity at m/z 342.2 was used as a reference signal to correct for instrumental variability as well as sensitivity drift.

For signals of sufficient intensity, fragmentation spectra in the m/z range of 100 up to the m/z value of molecular ion were collected. Due to the fragmentation parameters not optimized for each substance, only the most intense signals yielded useful results. Obtained fragmentation spectra with proposed identification and fragments are presented from Figure S23 to Figure S33.

It should be noted that both: proposed fragmentation and identification were not verified on basis of reference standards, therefore they cannot be considered as conclusive.

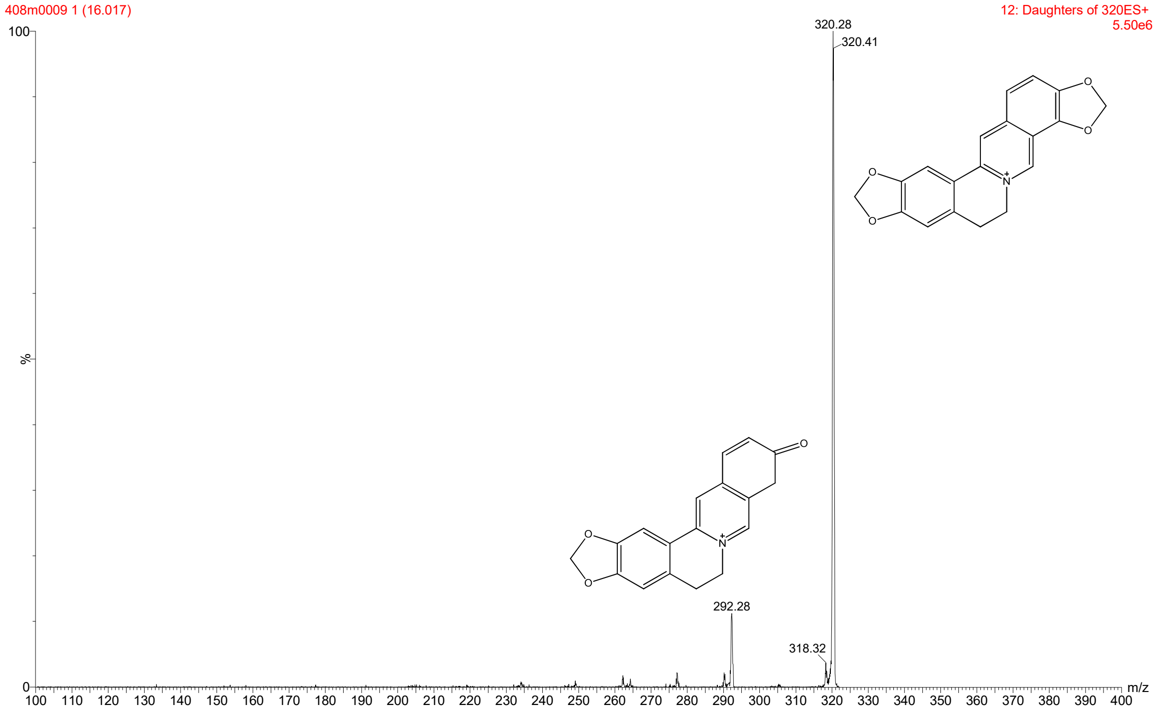

Figure S23. ESI (+) fragmentation spectrum of an ion at m/z = 320. Fragmentation pattern indicates possible presence of coptisine

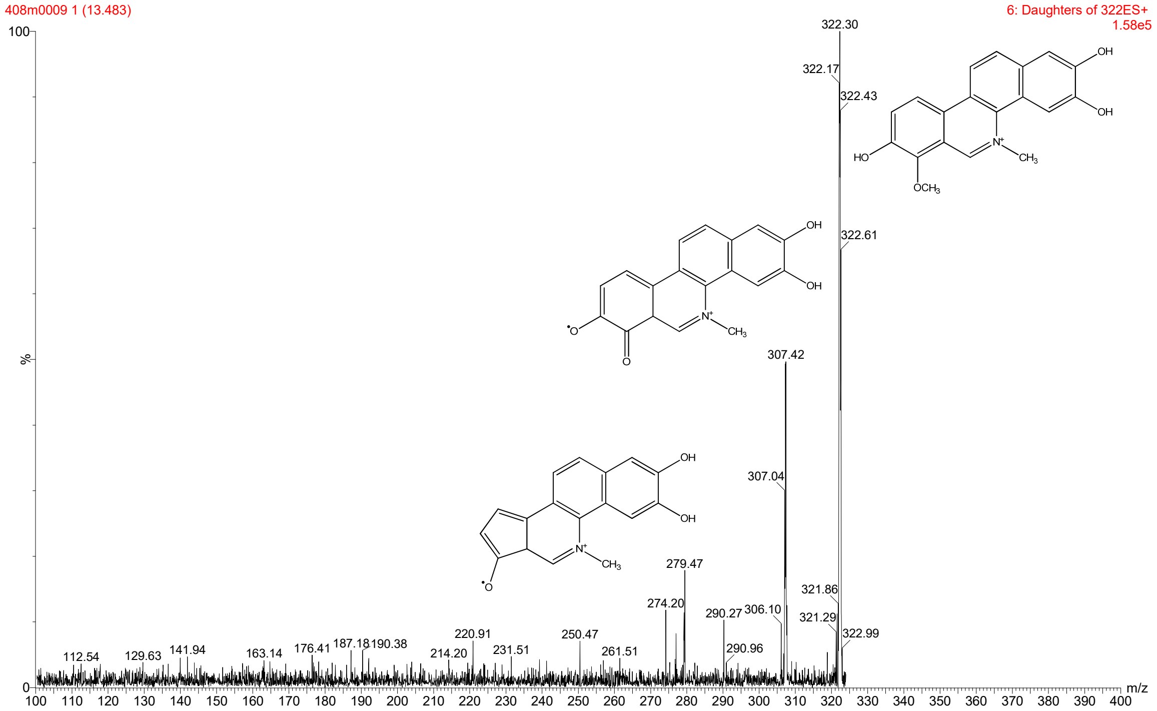

Figure S24. ESI (+) fragmentation spectrum of an ion at m/z = 322. Fragmentation pattern indicates possible presence of 8-hydroxychelerythrine

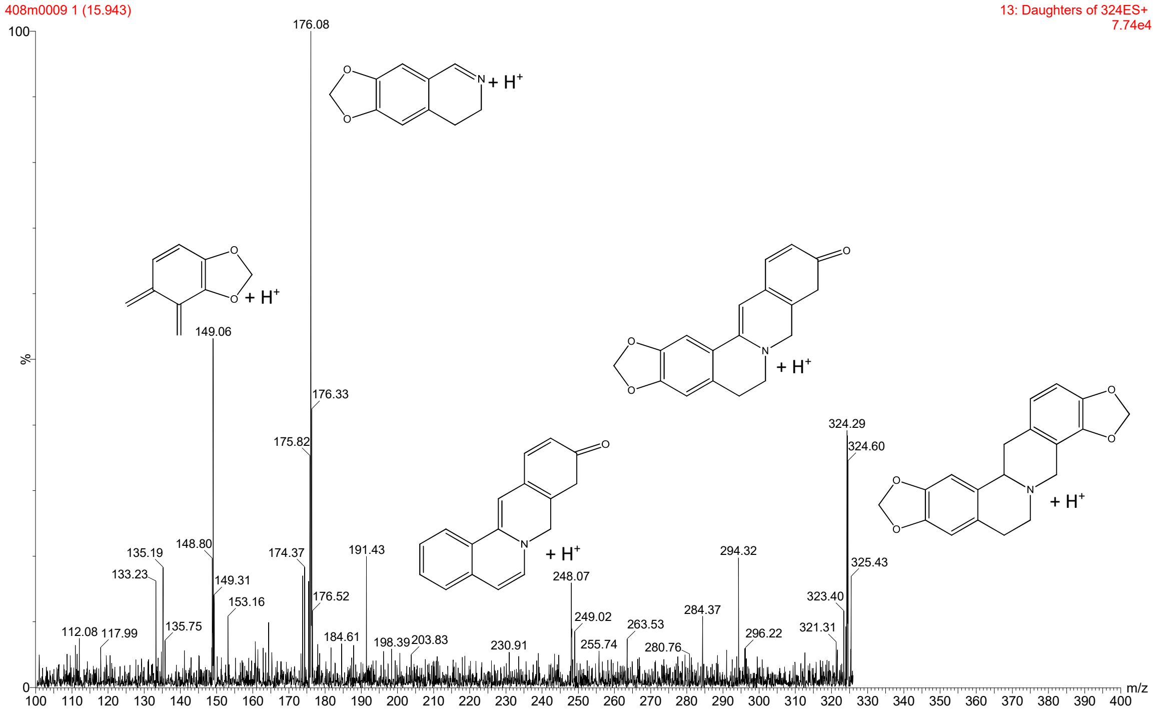

Figure S25. ESI (+) fragmentation spectrum of an ion at m/z = 324. Fragmentation pattern indicates possible presence of stylopine

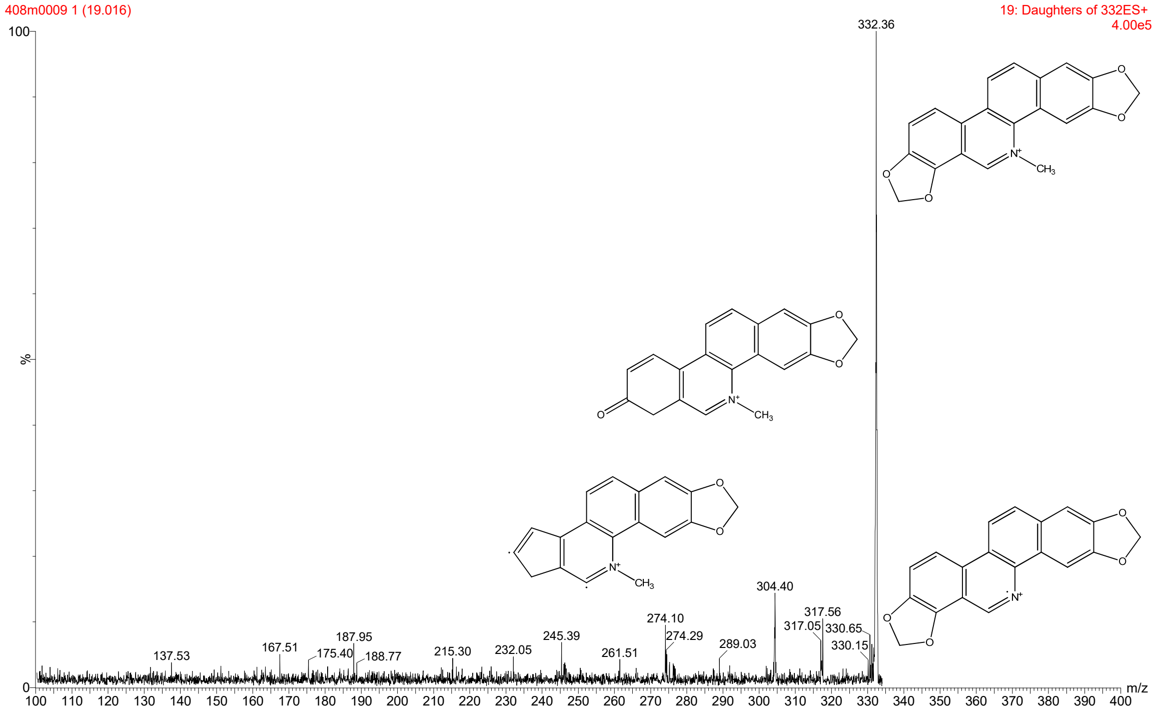

Figure S26. ESI (+) fragmentation spectrum of an ion at m/z = 332. Fragmentation pattern indicates possible presence of sanguinarine

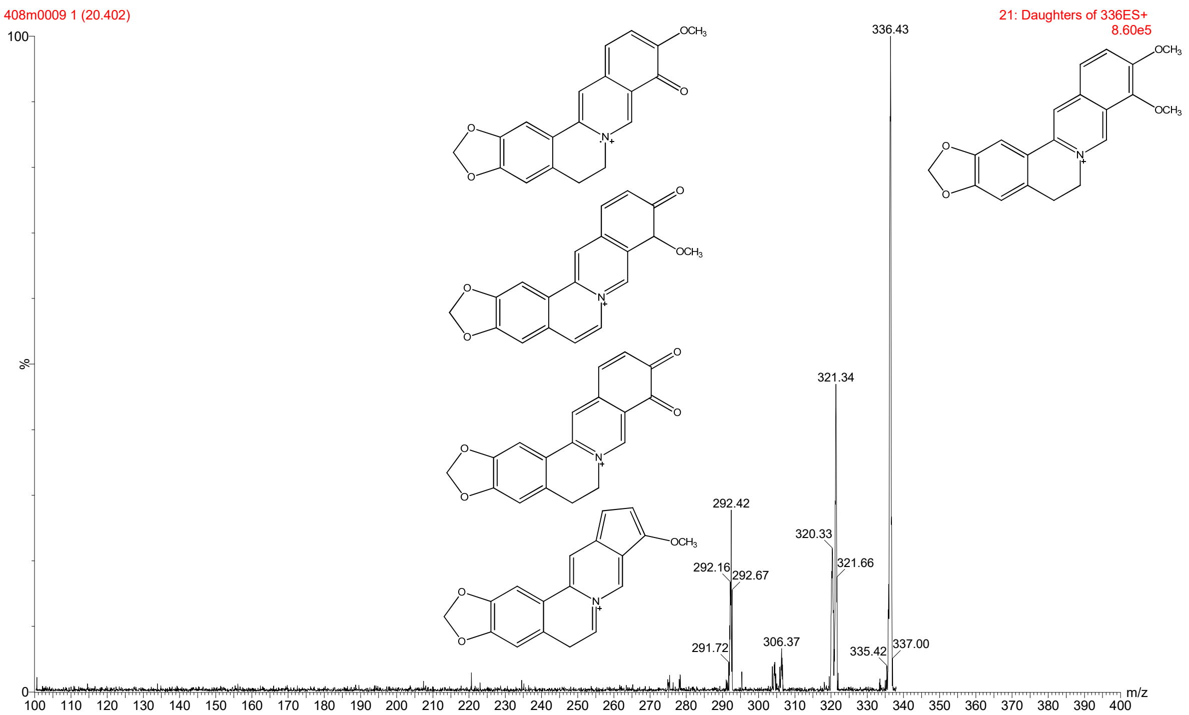

Figure S27. ESI (+) fragmentation spectrum of an ion at m/z = 336. Fragmentation pattern indicates possible presence of berberine

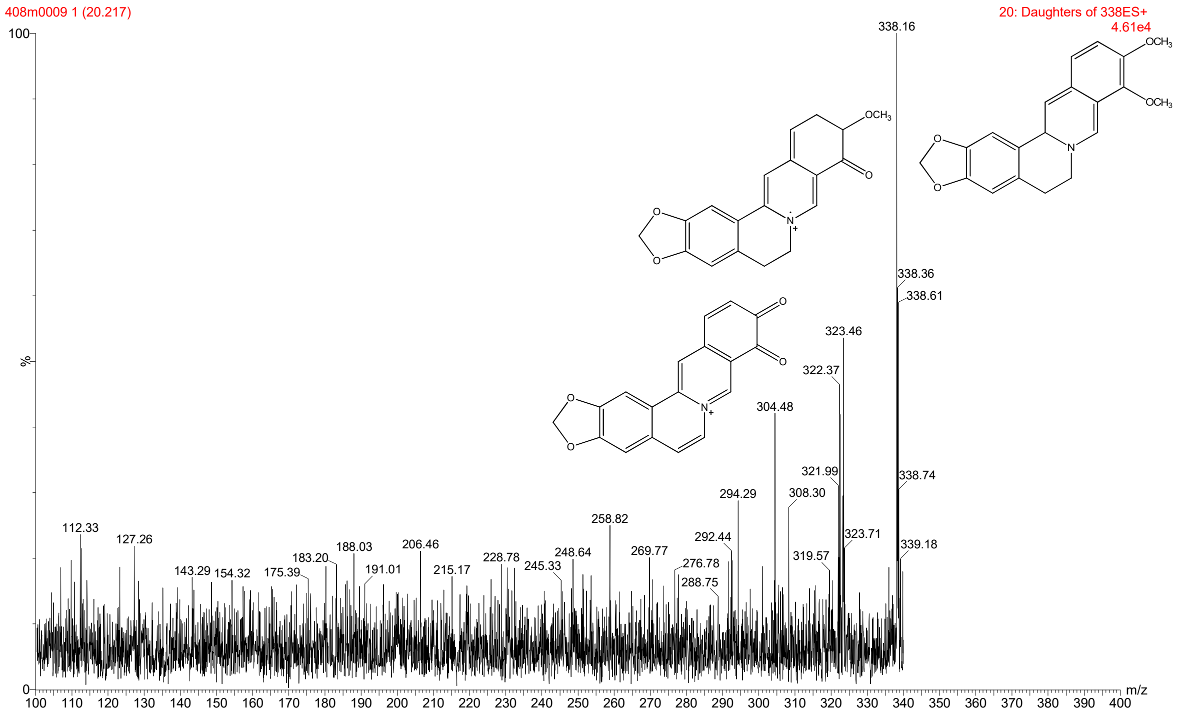

Figure S28. ESI (+) fragmentation spectrum of an ion at m/z = 338. Fragmentation pattern indicates possible presence of dihydroberberine

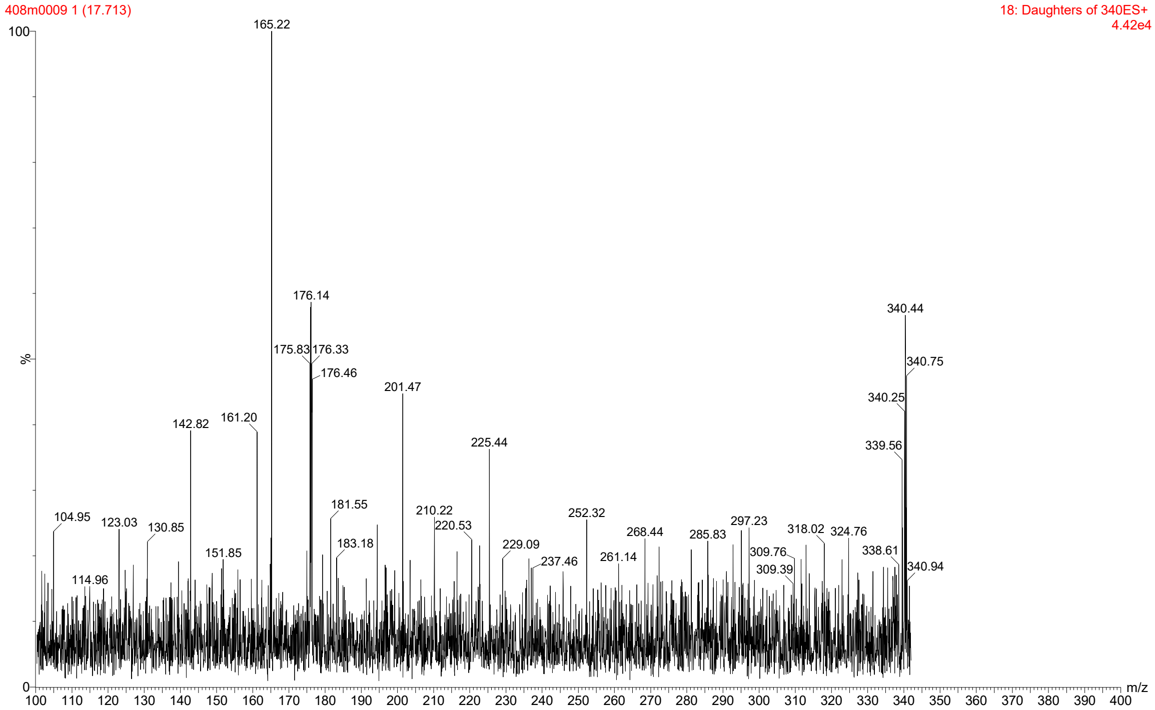

Figure S29. ESI (+) fragmentation spectrum of an ion at m/z = 340. Possible presence of norchelidonine or canadine.

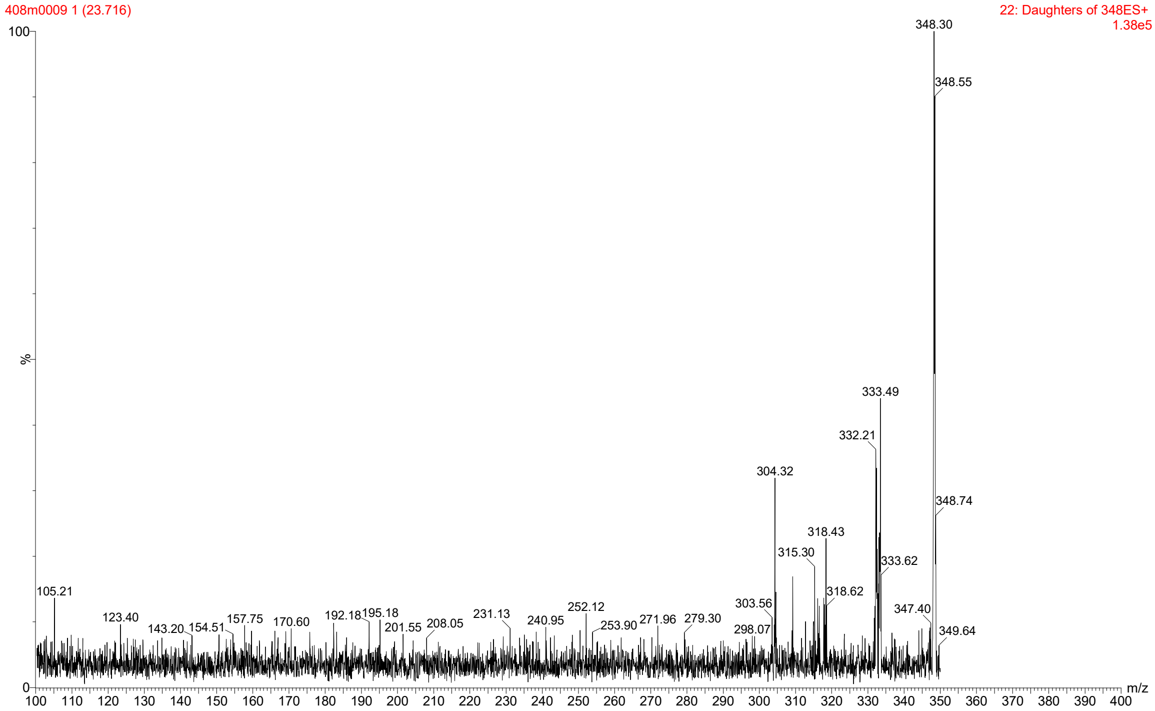

Figure S30. ESI (+) fragmentation spectrum of an ion at m/z = 348. Possible presence of oxysanguiarine, chelerythrine or nitidine

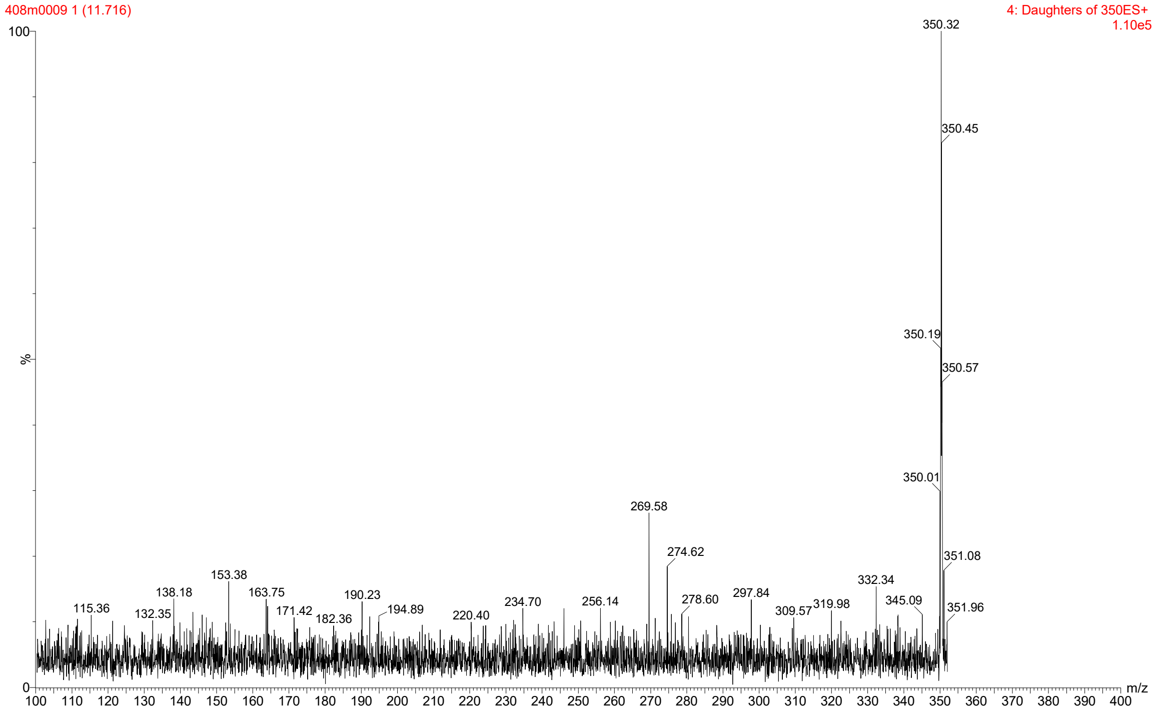

Figure S31. ESI (+) fragmentation spectrum of an ion at m/z = 350. Possible presence of isochelidonine, dihydrochelerythrine or dihydronitidine

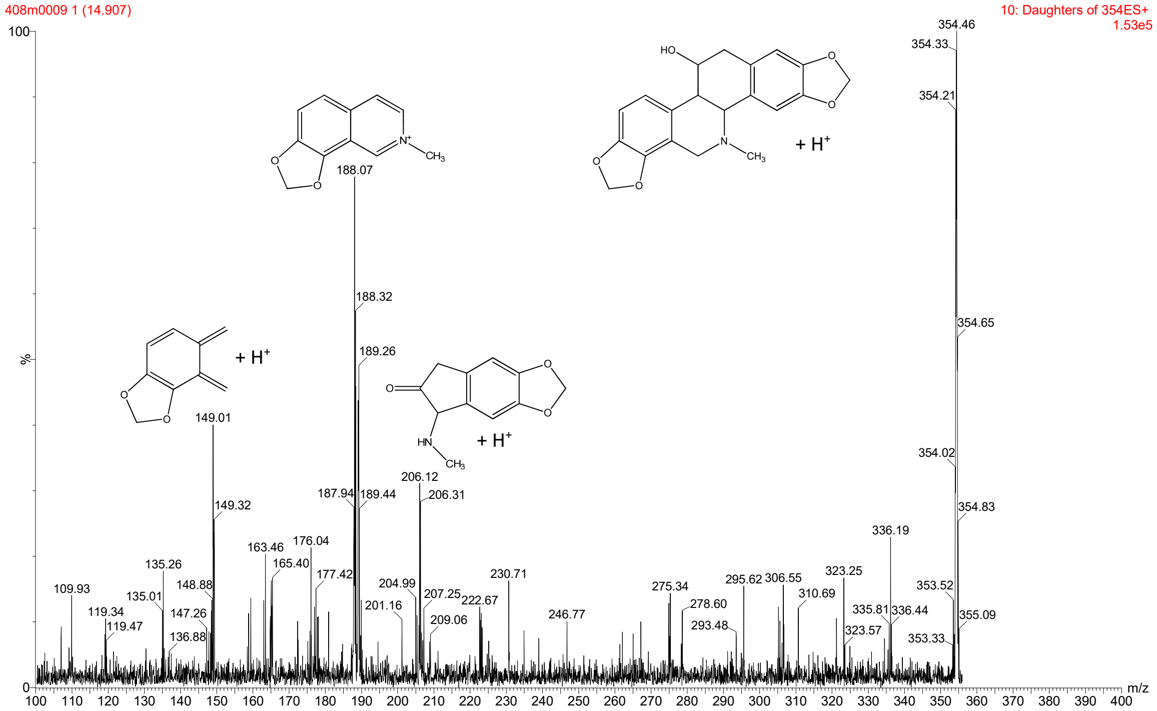

Figure S32. ESI (+) fragmentation spectrum of an ion at m/z = 354. Fragmentation pattern indicates possible presence of chelidonine

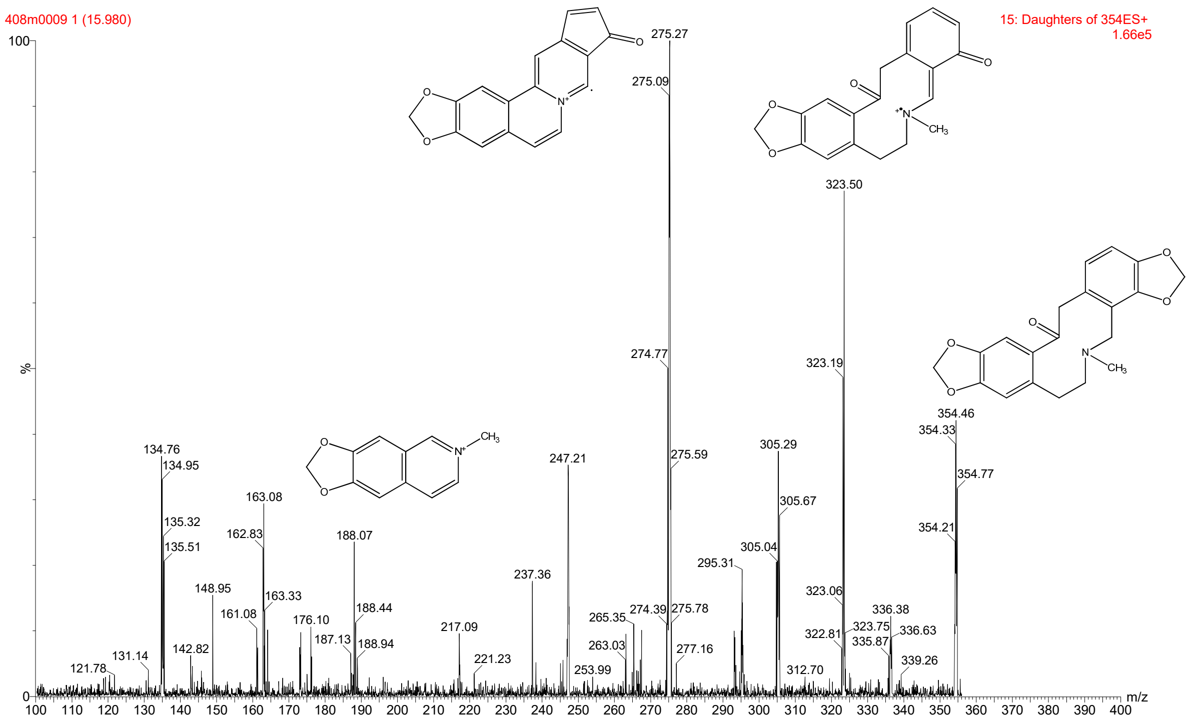

Figure S33. ESI (+) fragmentation spectrum of an ion at m/z = 354. Fragmentation pattern indicates possible presence of protopine

Figures S34 – S47, Tables S4 – S5: Semi-quantitative relative comparison of alkaloids content in *C. majus* latex fractions

On the basis of chromatograms obtained for samples containing highest number of constituents (early chromatographic fractions, data not shown), 21 signals were selected for indirect comparison on the basis of a peak areas ratio of given substance to the reference signal (**Error! Reference source not found.**). 20 signals were monitored from selected-ion monitoring (SIM) chromatograms and one (m/z = 326.2) was extracted from total ion current (TIC) chromatogram of scan spectra in m/z range of 300-400.

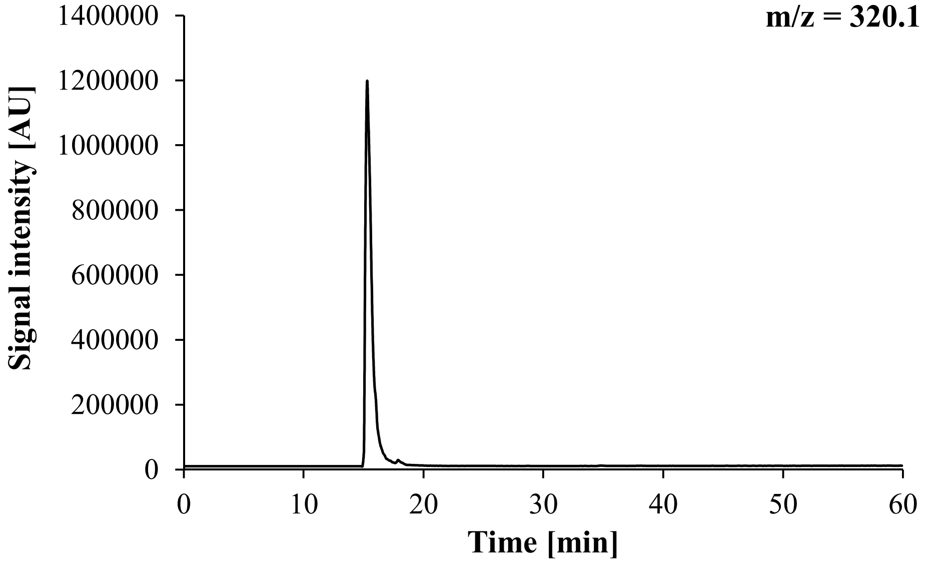

Figure S34. SIM chromatogram obtained for the sample coded FT 6.4 at m/z value of 320.1.

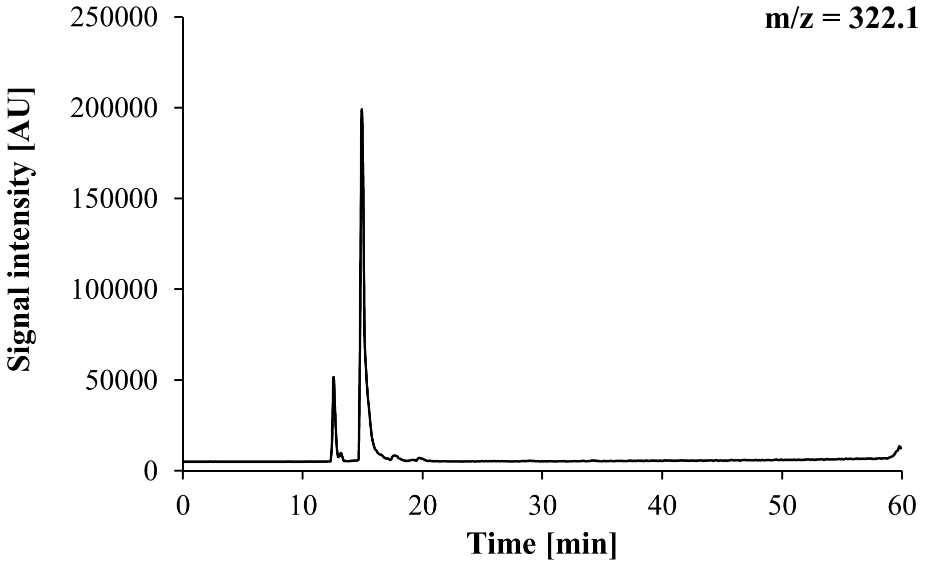

Figure S35. SIM chromatogram obtained for the sample coded FT 6.4 at m/z value of 322.1

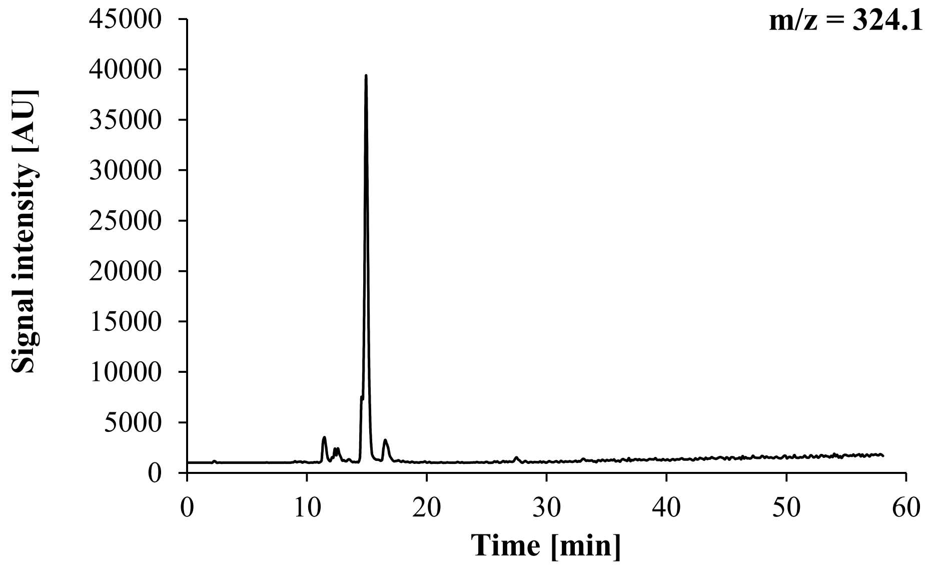

Figure S36. SIM chromatogram obtained for the sample coded FT 6.4 at m/z value of 324.1

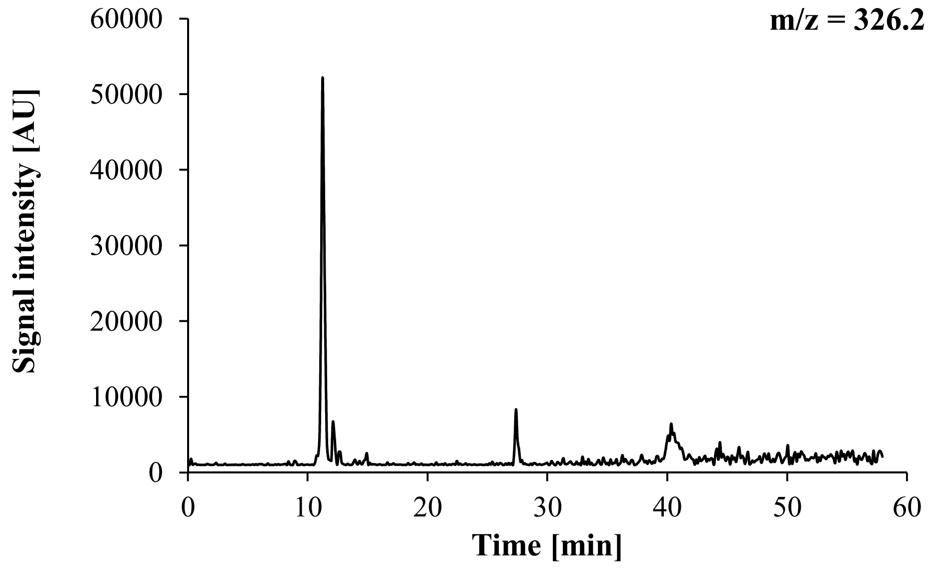

Figure S37. Extracted chromatogram at m/z value of 326.2 from TIC obtained for the sample coded FT 6.4

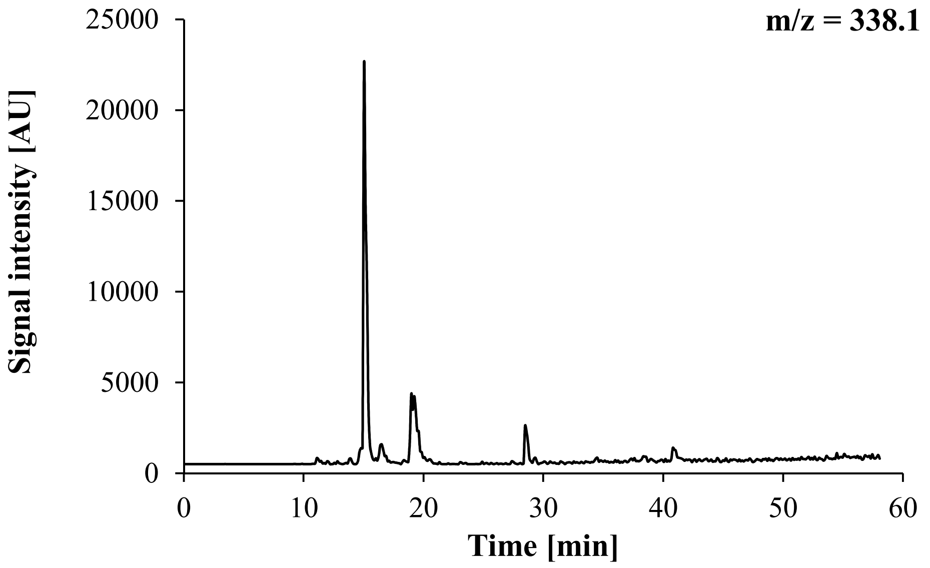

Figure S38. SIM chromatogram obtained for the sample coded FT 6.4 at m/z value of 338.1

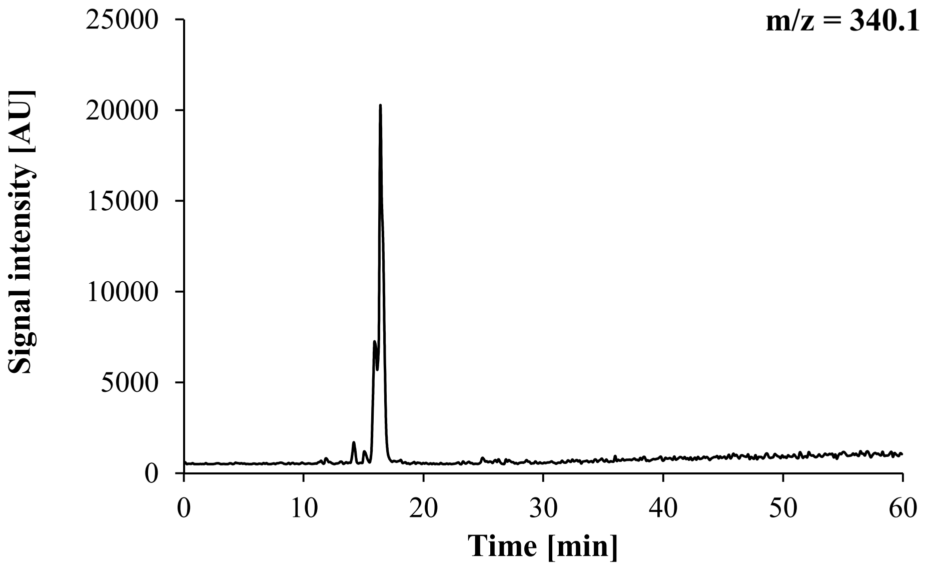

Figure S39. SIM chromatogram obtained for the sample coded FT 6.4 at m/z value of 340.1

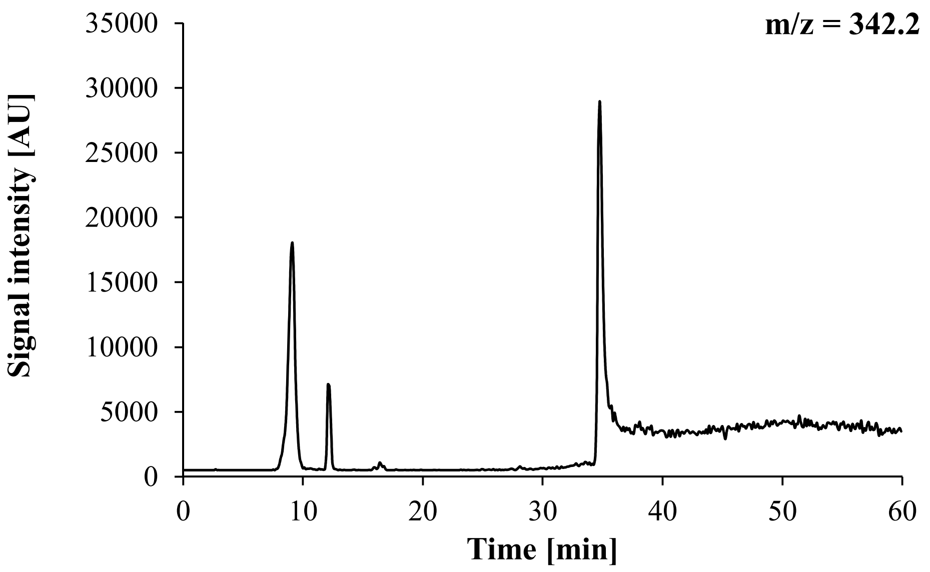

Figure S40. SIM chromatogram obtained for the sample coded FT 6.4 at m/z value of 342.2

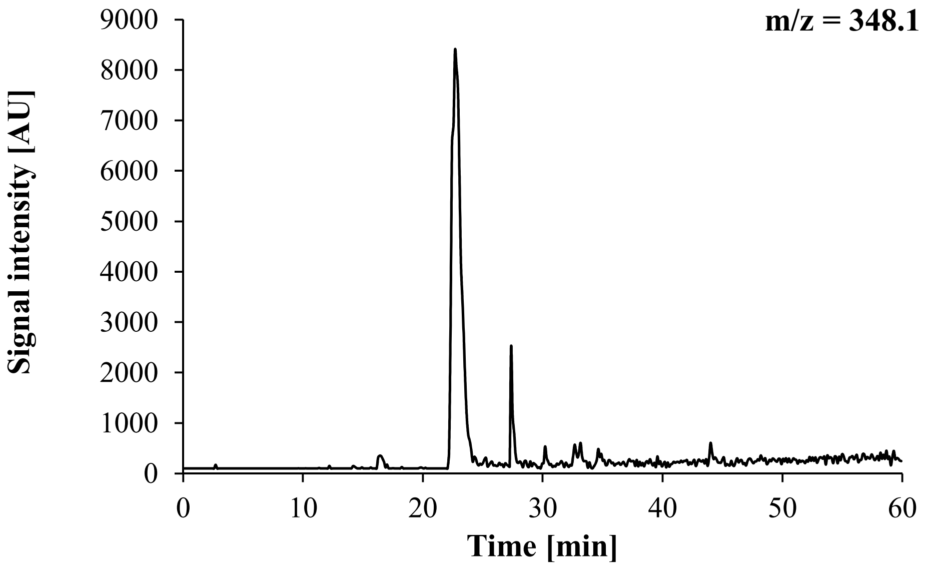

Figure S41. SIM chromatogram obtained for the sample coded FT 6.4 at m/z value of 348.1

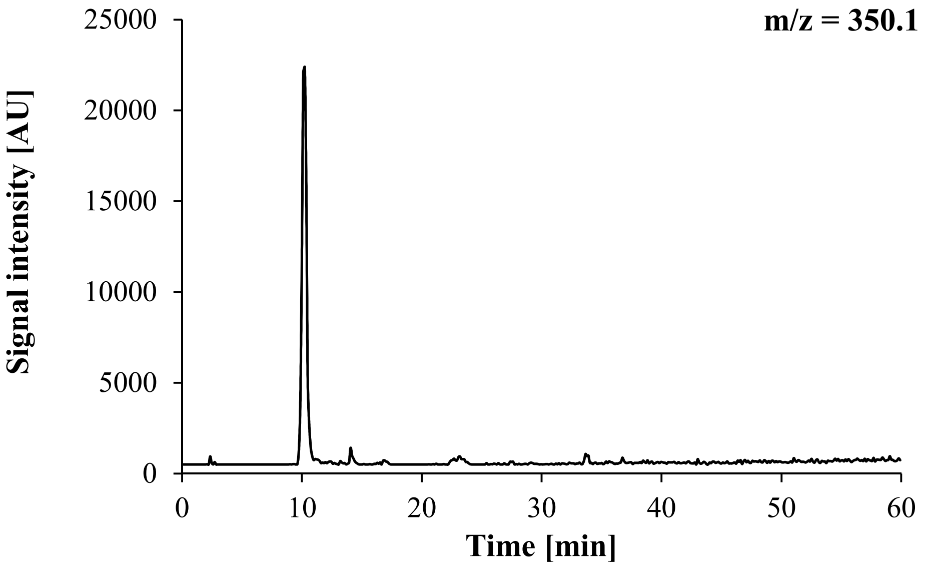

Figure S42. SIM chromatogram obtained for the sample coded FT 6.4 at m/z value of 350.1

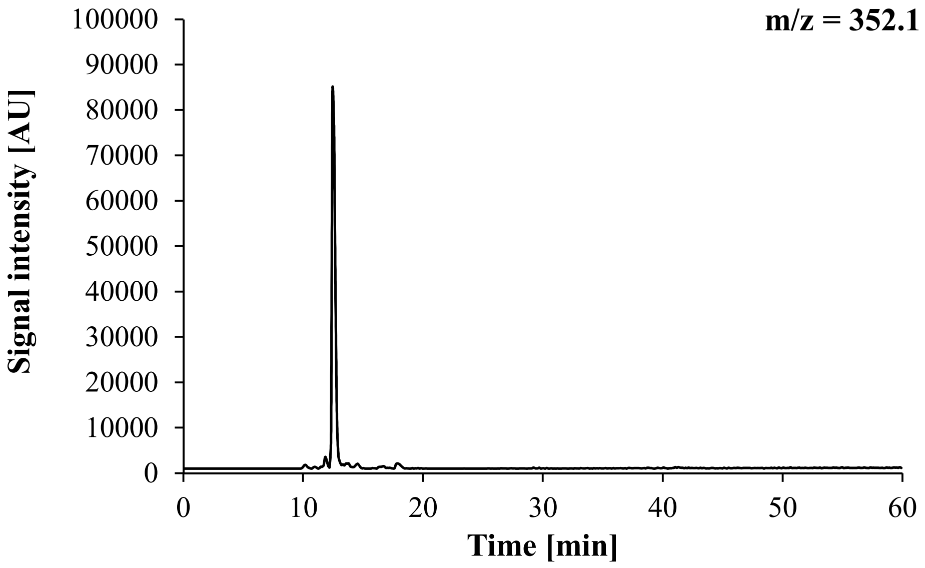

Figure S43. SIM chromatogram obtained for the sample coded FT 6.4 at m/z value of 352.1

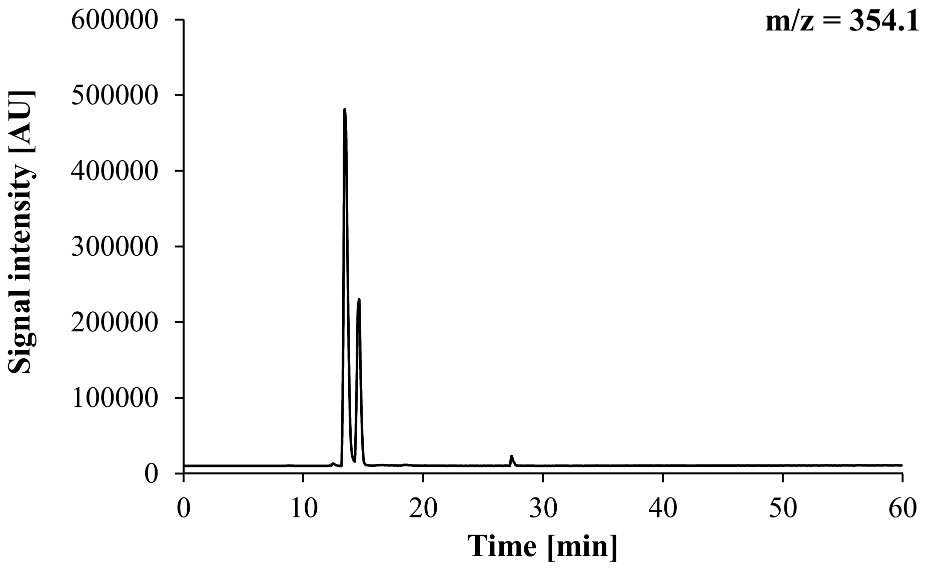

Figure S44. SIM chromatogram obtained for the sample coded FT 6.4 at m/z value of 354.1

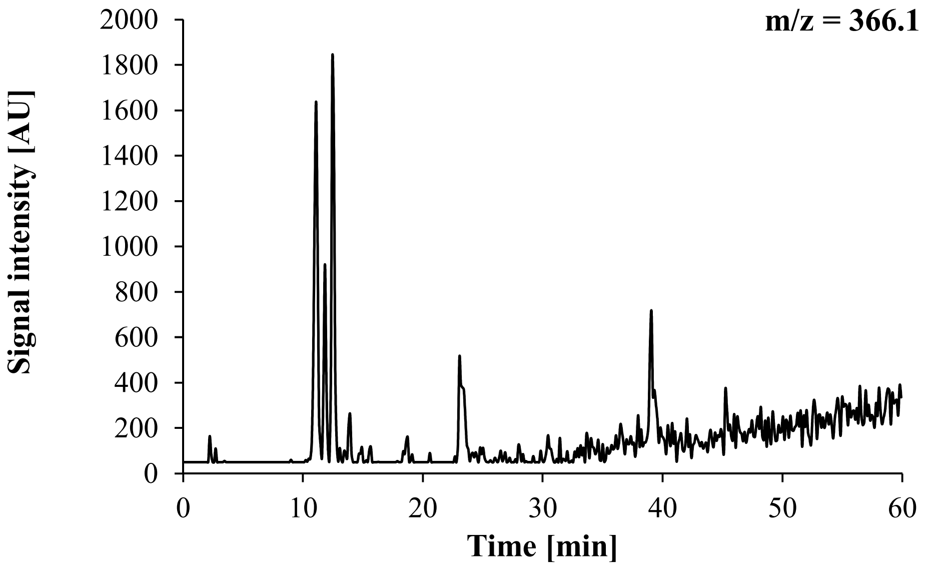

Figure S45. SIM chromatogram obtained for the sample coded FT 6.4 at m/z value of 366.1

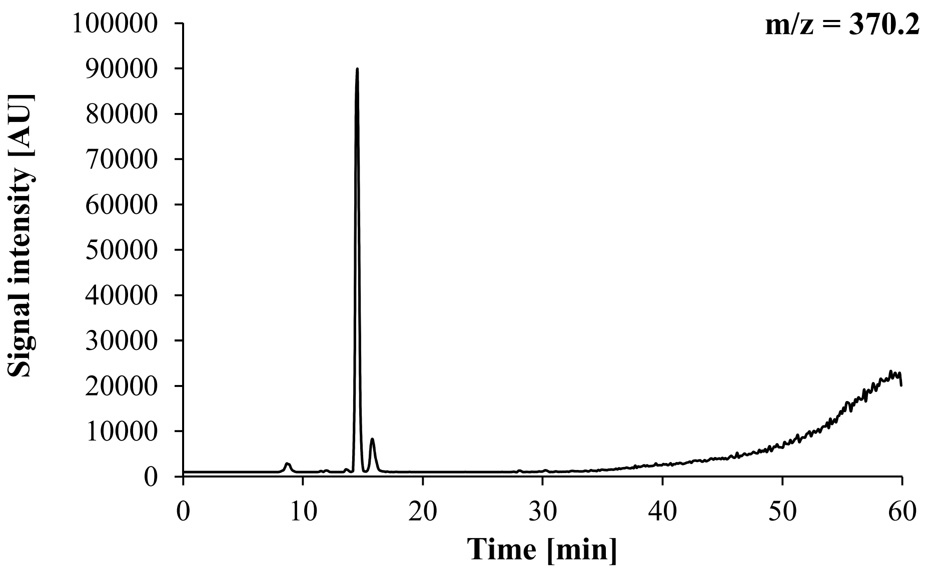

Figure S46. SIM chromatogram obtained for the sample coded FT 6.4 at m/z value of 370.2

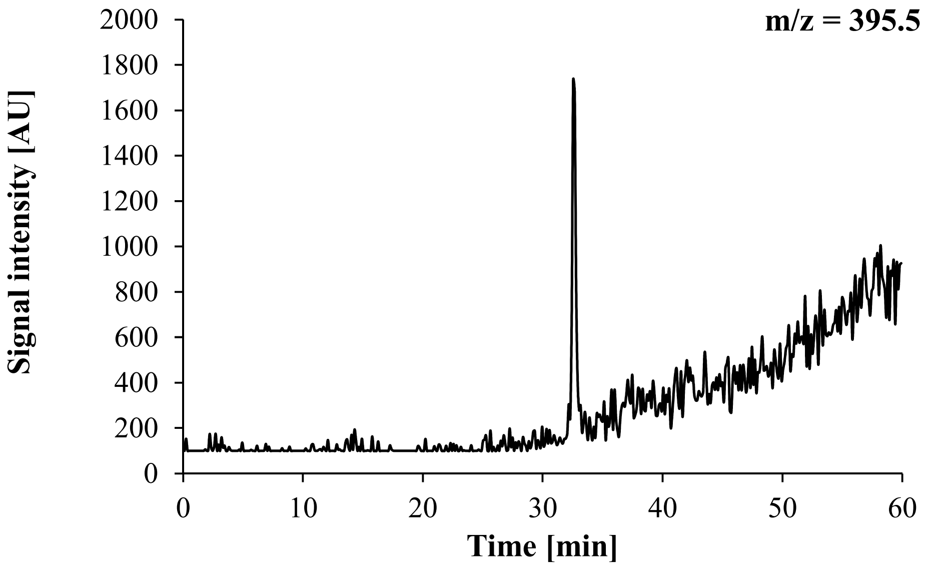

Figure S47. SIM chromatogram obtained for the sample coded FT 6.4 at m/z value of 395.5

Table S4. Signals selected for indirect comparison of quantities

No.	m/z (proposed substance)	t_r [min]
1	320.1 (coptisine)	15.29
2	320.1 (unknown constituent)	17.87
3	322.1 (8-hydroxychelerythrine)	12.57
4	324.1 (stylopine)	16.52
5	326.2 (unknown constituent)	11.24
6	332.2 (sanguinarine)	17.87
7	336.1 (berberine)	19.11
8	338.1 (dihydroberberine)	15.04
9	340.1 (unknown constituent)	16.39
10	342.2 (unknown constituent)	9.12
11	342.2 (unknown constituent)	12.08
12	348.1 (unknown constituent)	22.68
13	350.1 (unknown constituent)	10.23
14	352.1 (unknown constituent)	12.45
15	354.1 (chelidonine)	14.67
16	354.1 (protopine)	13.44
17	366.1 (unknown constituent)	12.45
18	370.2 (unknown constituent)	14.55
19	370.2 (unknown constituent)	15.78
20	395.5 (unknown constituent)	32.54
21	342.2 (signal used as reference)	34.76

Relative signal intensities (peak areas) are presented in Table S5. Peak area for each substance was divided by peak area of a reference signal (no. 21) and given as percent value, therefore the reference has always 100% relative intensity. The resulted ratios were then compared between fractions in given sample group for given substance. Color coding ranges from red (lowest relative intensity in a group) up to green signifying the highest relative intensity in a group. Samples coded FT were not included in the comparison, as they are an early fractions of IEC, therefore they contain relatively large amount of constituents at higher concentrations comparing to the fractions tested for cytotoxic activity.

Table S5. Relative signal intensities for each sample constituent in *C. majus* latex fractions after heparin chromatography. Fractions 22-26 contained MLP protein.

No.	m/z (proposed substance)	S6 19-20	S6 21	S6 22-23	S6 24	S6 25-26	S6 27-28
1	320.1 (coptisine)	608.38%	1088.52%	1563.94%	1360.98%	1327.16%	1143.77%
3	322.1 (8-hydroxychelerythrine)	4.98%	7.56%	13.51%	13.59%	15.04%	11.91%
4	324.1 (stylopine)	0.00%	0.00%	0.00%	0.00%	0.08%	0.00%
6	332.2 (sanguinarine)	1.19%	2.79%	2.91%	6.05%	7.84%	4.35%
7	336.1 (berberine)	0.00%	62.91%	92.84%	84.60%	74.73%	63.24%
8	338.1 (dihydroberberine)	0.00%	2.35%	2.88%	1.53%	1.27%	0.39%
15	354.1 (chelidonine)	106.41%	145.26%	157.33%	124.89%	126.30%	91.66%
16	354.1 (protopine)	0.00%	18.88%	19.03%	11.02%	8.11%	3.97%
21	342.2 (signal used as reference)	100.00%	100.00%	100.00%	100.00%	100.00%	100.00%

```
m.1784|low              ------------------------------------------------------------    0
m.62644|mlp-like        --------------------------------------------------MAQIHKLELQ    10
m.29772|mlp-like        ----------------------------------------------------------VE    2
m.29773|mlp-like        --------------TAYIEFVKRVTKDLNSKLHHLGHSHIGHSHKGHPHKGSNVEKLEAE    46
m.29774|mlp-like        IVEYEKAKEDAPEPTAYIEFVKRVTKDLNSKLHHLGH-----SHKVHPHKGSNVEKLEAE    55
m.12630|mlp-like        ------------------------------------------------------------    0
m.15473|mlp-like        ------------------------------------------------------------    0
m.12632|mlp-like        -------YEDAPAPNAYLDWLERVTKDLNNHLH-------------HSHSGSNVEKLVAE    40
m.12634|mlp-like        -------YEDAPAPNAYLDWLERVTKDLNNHLH-------------HSHSGSNVEKLVAE    40
m.60858|mlp-like        ---------------------------------------------MAA-IANVHYKLQAE    14
m.18123|mlp-like        ---------------------------------------------MVVAANNDIEKLEVE    15
m.37903|mlp-like        ---CITTTTTCSVSSSK------------VQVLIYT-------IIMASIANNNIEKLEVE    38
m.37899|mlp-like        -AVYVKAYEDAPIPAKYIEFLVGFTKDLDAHLQKNH-------GHTHAVGDNNIEKIEVE    52
m.37898|mlp-like        ---CITTTTTCSVSSSK------------VQVLIYT-------IIMASIANNNIEKLEVE    38
m.37895|mlp-like        -AVYVKAYEDAPIPAKYIEFLVGFTKDLDAHLQKNH-------GHTHAVGDNNIEKIEVE    52
m.37902|mlp-like        -AVYVKAYEDAPIPAKYIEFLVGFTKDLDAHLQKNH-------GHTHAVGDNNIEKIEVE    52
m.37897|mlp-like        -AVYVKAYEDAPIPAKYIEFLVGFTKDLDAHLQKNH-------GHTHAVGDNNIEKIEVE    52
m.37900|mlp-like        -AVYVKAYEDAPIPAKYIEFLVGFTKDLDAHLQKNH-------GHTHAVGDNNIEKIEVE    52
m.37901|mlp-like        -AVYVKAYEDAPIPAKYIEFLVGFTKDLDAHLQKNH-------GHTHAVGDNNIEKIEVE    52

m.1784|low              ----------------------------------LEGDGKSVNSVRLWKYVIPGSSNFST    26
m.62644|mlp-like        TEVKCSADQLYGLFKNNITHLSKYYPECYKTIQVIQGDGPSVGSVRLWKYILD--GTSVV    68
m.29772|mlp-like        IEVQCSGDKFYRMFKHDVDNISKLIPHHFEQVETLEGDGLSVGHVKHWYYVLEEGGRCLN    62
m.29773|mlp-like        IEVQCSADKFYRMFKHDVDNISKLIPHHFEQVETLEGDGLSVGHVKHWYYVLEEGGRCLN    106
m.29774|mlp-like        IEVQCSADKFYRMFKHDVDNISKLIPHHFEQVETLEGDGLSVGHVKHWYYVLEEGGRCLN    115
m.12630|mlp-like        -------------------------------------------TVKLWKYKLE--GKSLY    15
m.15473|mlp-like        --------------------------------------------VKRWHYVLEEGGRCLT    16
m.12632|mlp-like        IEVQCSADKFYRMYKNEVKELSKHIPHHYEDVEILEGDGISVGSVKRWHYVLEEGGRCLT    100
m.12634|mlp-like        IEVQCSADKFYRMYKNEVKELSKHIPHHYEDVEILEGDGISVGSVKRWHYVLEEGGRCLT    100
m.60858|mlp-like        IEVKCCADKFYRMFKHEAEELPTHLPHHFQTVEVLEGDGVSVGTVKLWKYTLD--GKSLY    72
m.18123|mlp-like        VEVQYPAHNFYRIFKHDVKEVPKLVPHLYPSVQVLEGDGISPGTVKLWTYQLE--GKQFR    73
m.37903|mlp-like        VDVQCSPHKFYRMFKHDVKELPKHLPHLIESVEVLEGDGVSVGTVKLWKYILE--GKHLY    96
m.37899|mlp-like        VEVPYDADKFYHMFKYDAKEIPKHLPHLIEHVEVLEGDGVSVGTVKLWKYILE--GKHLY    110
m.37898|mlp-like        VDVQCSPHKFYRMFKHDVKELPKHLPHLIESVEVLEGDGVHAGTVKLWKYKLE--GKTLY    96
m.37895|mlp-like        VEVPYDADKFYHMFKYDAKEIPKHLPHLIEHVEVLEGDGITADSVKLWKYLLA-------    105
m.37902|mlp-like        VEVPYDADKFYHMFKYDAKEIPKHLPHLIEHVEVLEGDGITADSVKLWKYLLA-------    105
m.37897|mlp-like        VEVPYDADKFYHMFKYDAKEIPKHLPHLIEHVEVLEGDGITADSVKLWKYLLA-------    105
m.37900|mlp-like        VEVPYDADKFYHMFKYDAKEIPKHLPHLIEHVEVLEGDGVHAGTVKLWKYKLE--GKTLY    110
m.37901|mlp-like        VEVPYDADKFYHMFKYDAKEIPKHLPHLIEHVEVLEGDGVHAGTVKLWKYKLE--GKTLY    110

m.1784|low              VKEKIKVVDNENRSITFKIMEGDLMNIYKSYESKLSV-TPTTG---N---GCKVKWDLEF    79
m.62644|mlp-like        CKERVTMVDDERKSITWSIFEGEVMNFYNTLELKLVSVTPKNDG------SSLVKWCVEF    122
m.29772|mlp-like        VKERTTVLDDEKRMVTHSFFEGEVMKDYKFFELTLVV-SPKEGGDSH-------------    108
m.29773|mlp-like        VKERTTVLDDEKRMVTHSFFEGEVMNDYKFFEITLVV-TPKEG-----------------    148
m.29774|mlp-like        VKERTTVLDDEKRMVTHSFFEGEVMNDYKFFEITLVV-TPKEG-----------------    157
m.12630|mlp-like        SKERMTVVDDEKKIITHSVIEGDLLKDYKCFVVTLII-TPKG-GHGDG---SVVKWIVEY    70
m.15473|mlp-like        AKERITFVDDEKRMVTHSIFEGDVMNDYKLLDITLVV-TPKEAGDSNG---CVVKWIVEF    72
m.12632|mlp-like        AKERTTVLDDEKRIITHSVFEGEPMNDYKFLDLTLVV-TPKEGCDSNG---SVVKWIVEY    156
m.12634|mlp-like        AKERTTVLDDEKRIITHSVFEGEPMNDYKFLDLTLVV-TPKEGCDSNG---SVVKWIVEY    156
m.60858|mlp-like        LKERMTVVDDEKRMITHSNFEGDIMKDYKRLDMGFAV-NPKGG-DSAN--GSLVTLTVDF    128
m.18123|mlp-like        VKERTTIVDDGKRMITHCALEGEVMKDYKCFNRTFTV-DPKDG-HVEGREVSVVKWQIEY    131
m.37903|mlp-like        VKERMTVADDEKKMITHNIFEGEVMKDYKCLSKTFTV-NPKGG-HGEG---SVVKWHLEY    151
m.37899|mlp-like        VKERMTVADDEKKMITHNIFEGEVMKDYKCLSKTFTV-NPKGG-HGEG---SVVKWHLEY    165
m.37898|mlp-like        LKERMTVVDDEKKIITYSILEGDLLKDYKCFGATFTA-TPKGG-HGDG---SVVKWILEY    151
m.37895|mlp-like        ------------------------------------------------------------    105
m.37902|mlp-like        ------------------------------------------------------------    105
m.37897|mlp-like        ------------------------------------------------------------    105
m.37900|mlp-like        LKERMTVVDDEKKIITYSILEGDLLKDYKCFGATFTA-TPKGG-HGDG---SVVKWILEY    165
m.37901|mlp-like        LKERMTVVDDEKKIITYSILEGDLLKDYKCFGATFTA-TPKGG-HGDG---SVVKWILEY    165

m.1784|low              EKVSEDVPNPDDILDLLAFMNKEFGRYLLNN*----------------------------    110
m.62644|mlp-like        EKANDDAPTPTGYITLLEKISKEMHLHM*-------------------------------    150
m.29772|mlp-like        ------------------------------------------------------------    108
```

```
m.29773|mlp-like      ------------------------------------------------------------      148
m.29774|mlp-like      ------------------------------------------------------------      157
m.12630|mlp-like      EKATEDVHVPANYIEFAVWLTKDLDGSLPNHAHT--HAVGANNIEKIEGEVEVQCDADKF      128
m.15473|mlp-like      EKAKEDAPTPTVYIELKKRITRDFDNHLHKHP*---------------------------      104
m.12632|mlp-like      EKATEDVHVPANYIEFAVWLTKDLDGSLPNHAHT--HAVGANNIEKIEGEVEVQCDADKF      214
m.12634|mlp-like      EKAREDAPIPTAYIELKKRVTRDLNSHLHKHA*---------------------------      188
m.60858|mlp-like      EKANEDAPTPTAYIDFIIRVTKDVESHILNQK*---------------------------      160
m.18123|mlp-like      VKAHEHAPVPSKYIEFFVCLTKDLSAKLHNNQDGHSLIVGDNNIERIEVEVEV-------      184
m.37903|mlp-like      AKAREDVHIPTDYIHYCVWLTKDLTAHLHKDA*---------------------------      183
m.37899|mlp-like      AKAREDVHIPTDYIHYCVWLTKDLTAHLHKDA*---------------------------      197
m.37898|mlp-like      EKATKDAPIPANYIEFLVWVTKDLNAHLHNRSHS--HSVGADNIEKIEVEVEVQSDADKF      209
m.37895|mlp-like      ------------------------------------------------------------      105
m.37902|mlp-like      ------------------------------------------------------------      105
m.37897|mlp-like      ------------------------------------------------------------      105
m.37900|mlp-like      EKATKDAPIPANYIEFLVWVTKDLNAHLHNRSHS--HSVGADNIEKIEVEVEVQSDADKF      223
m.37901|mlp-like      EKATKDAPIPANYIEFLVWVTKDLNAHLHNRSHS--HSVGADNIEKIEVEVEVQSDADKF      223

m.1784|low            ------------------------------------------------------------      110
m.62644|mlp-like      ------------------------------------------------------------      150
m.29772|mlp-like      ------------------------------------------------------------      108
m.29773|mlp-like      ------------------------------------------------------------      148
m.29774|mlp-like      ------------------------------------------------------------      157
m.12630|mlp-like      YSMFKHDVKEIPKNIPHLY-----------------------------------------      147
m.15473|mlp-like      ------------------------------------------------------------      104
m.12632|mlp-like      YSMFKHDVKEIPKNIPHLY-----------------------------------------      233
m.12634|mlp-like      ------------------------------------------------------------      188
m.60858|mlp-like      ------------------------------------------------------------      160
m.18123|mlp-like      ------------------------------------------------------------      184
m.37903|mlp-like      ------------------------------------------------------------      183
m.37899|mlp-like      ------------------------------------------------------------      197
m.37898|mlp-like      YRLIKHDVKEIPKGVPHLFEHVEVLEGDGINAGSVKLWKYLLEGKHEYCKERMKVVDDEK      269
m.37895|mlp-like      ------------------------------------------GGKPLYCKERMKVVDDEK      123
m.37902|mlp-like      ------------------------------------------GGKPLYCKERMKVVDDEK      123
m.37897|mlp-like      ------------------------------------------GGKPLYCKERMKVVDDEK      123
m.37900|mlp-like      YRLIKHDVKEIPKGVPHLFEHVEVLEGDGINAGSVKLWKYLLEGKHEYCKERMKVVDDEK      283
m.37901|mlp-like      YRLIKHDVKEIPKGVPHLFEHVEVLEGDGINAGSVKLWKYLLEGKHEYCKERMKVVDDEK      283

m.1784|low            ------------------------------------------------------------      110
m.62644|mlp-like      ------------------------------------------------------------      150
m.29772|mlp-like      ------------------------------------------------------------      108
m.29773|mlp-like      ------------------------------------------------------------      148
m.29774|mlp-like      ------------------------------------------------------------      157
m.12630|mlp-like      ------------------------------------------------------------      147
m.15473|mlp-like      ------------------------------------------------------------      104
m.12632|mlp-like      ------------------------------------------------------------      233
m.12634|mlp-like      ------------------------------------------------------------      188
m.60858|mlp-like      ------------------------------------------------------------      160
m.18123|mlp-like      ------------------------------------------------------------      184
m.37903|mlp-like      ------------------------------------------------------------      183
m.37899|mlp-like      ------------------------------------------------------------      197
m.37898|mlp-like      RMITHSFFEGDVMKDYTYFDVIFTVTPKGGHGEGSVVKWDVEYVRAREDVPIPTNYLDHL      329
m.37895|mlp-like      RMITHSFFEGEVLKDYSFFDLTFTVTPKGGPGEGSVVKWHIEYVKAHEDVHIPSDYIDQL      183
m.37902|mlp-like      RMITHSFFEGEVLKDYSFFDLTFTVTPKGGPGEGSVVKWHIEYVKAHEDVHIPTDYIDQL      183
m.37897|mlp-like      RMITHSFFEGDVMKDYTYFDVIFTVTPKGGHGEGSVVKWDVEYVRAREDVPIPTNYLDHL      183
m.37900|mlp-like      RMITHSFFEGDVMKDYTYFDVIFTVTPKGGHGEGSVVKW---------------------      322
m.37901|mlp-like      RMITHSFFEGDVMKDYTYFDVIFTVTPKGGHGEGSVVKWDVEYVRAREDVPIPTNYLDHL      343

m.1784|low            ----------------  110
m.62644|mlp-like      ----------------  150
m.29772|mlp-like      ----------------  108
m.29773|mlp-like      ----------------  148
m.29774|mlp-like      ----------------  157
m.12630|mlp-like      ----------------  147
m.15473|mlp-like      ----------------  104
m.12632|mlp-like      ----------------  233
m.12634|mlp-like      ----------------  188
```

```
m.60858|mlp-like      ----------------  160
m.18123|mlp-like      ----------------  184
m.37903|mlp-like      ----------------  183
m.37899|mlp-like      ----------------  197
m.37898|mlp-like      IWTTKDLDAHMQKDV*  344
m.37895|mlp-like      VWITKDLTAHLHKDA*  198
m.37902|mlp-like      VWFTKDSTAHLHKDA*  198
m.37897|mlp-like      IWTTKDLDAHMQKDV*  198
m.37900|mlp-like      ----------------  322
m.37901|mlp-like      IWTTKDLDAHMQKDV*  358
```

Figure S48. Primary sequence alignment of 19 *C. majus* MLP-coding transcript sequences of different length (between 105 - 359 nucleotides) using CLUSTAL O(1.2.4) multiple sequence alignment (https://www.ebi.ac.uk/). Accesion numbers from *C. majus* CDS database derived from transcriptomic data (http://webblast.ipk-gatersleben.de/chelidonium/).

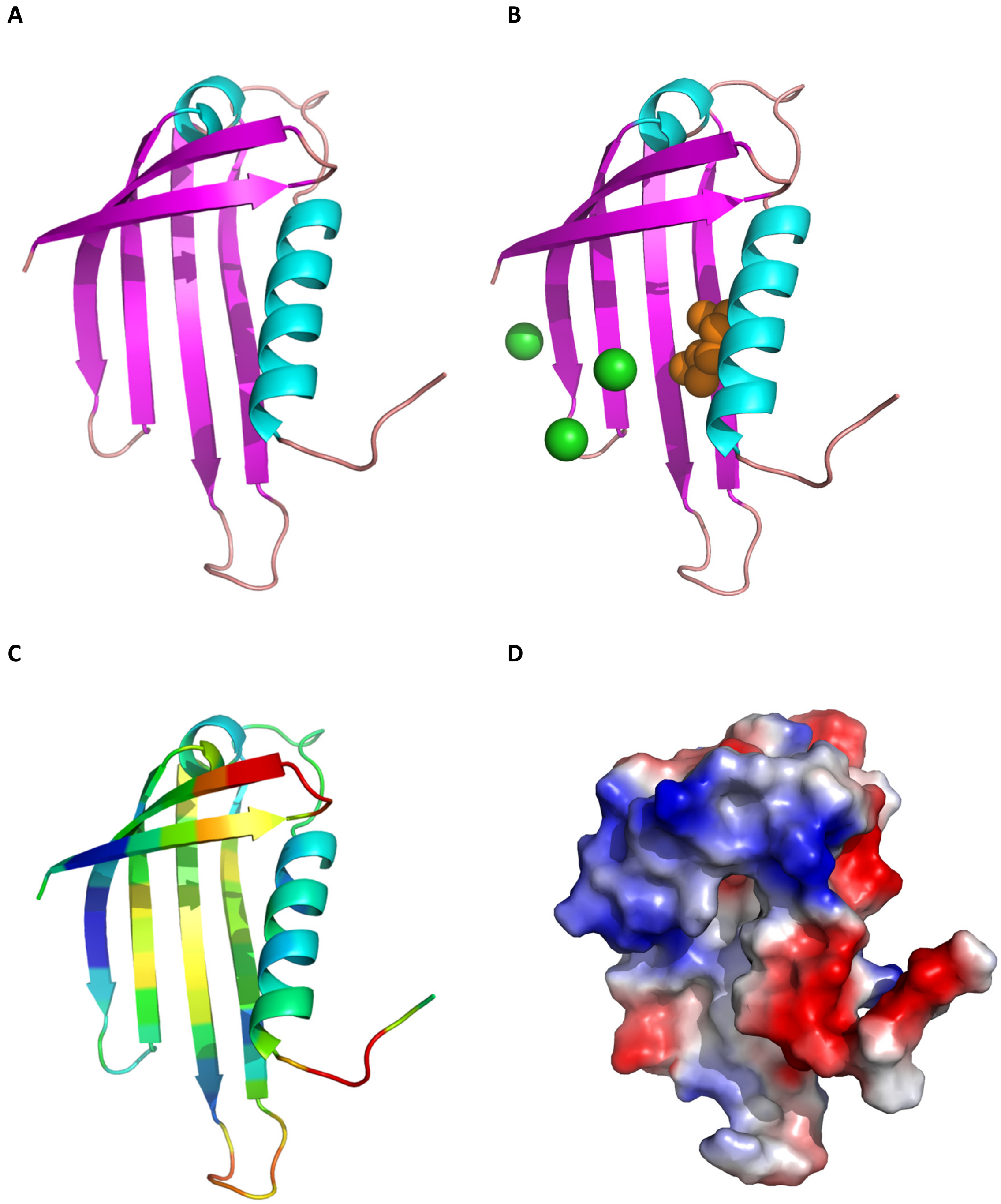

Figure S49. CmMLP1 protein modeling. (A) 3D model of *C. majus* MLP in the ribbon representation, colored by the elements of the secondary structure. (B) Additionally the location of Cl ions (green) and unknown ligand (orange) ions is shown, that were present in the crystal structure of the 4IGV homologous protein. (C) Coloring after assessing the quality of the model. Blue = protein-like. (D) Colored according to the distribution of the electrostatic surface potential calculated with ABPS (PyMol). Positively charged regions in blue, negatively – in red.

Tables 6 – 13: Molecular docking of alkaloids to CmMLP1.

Eight alkaloid structures retrieved from ZINC database were docked in the hydrophobic pocket of the CmMLP1. Docking of ligand molecules to CmMLP1 was performed using the Autodock Vina program. It analyzes 20 possible conformations of the ligand molecule at the active site of the protein. Binding energy in the active site was calculated for each conformation. For the ligand molecule in the conformation with the lowest binding energy value, interaction analysis with amino acid residues at the active site of the protein was performed taking into account hydrogen bonds, ionic, hydrophobic and π-electron interactions of aromatic rings (PoseView15).

Table S6. Molecular docking of sanguinarine (ZINC00000706) to CmMLP1. The conformation with the highest binding affinity was marked in red. 20 conformations of sangunarine were presented according to the order of their binding affinity – from the highest to the lowest.

Sanguinarine ZINC00000706: 39 atoms, 0 rot: [Time 3: srch 0 aln 0 m+c 1 pol 2] [Ncn 23: 3 5 793 823 54]	
No.	ZINC database no._conformation_no.: binding affinity, docking score
1	[ZINC00000706_000: 3.12 crash -0.80 polar 0.00]
2	[ZINC00000706_001: 3.03 crash -1.00 polar 0.03]
3	[ZINC00000706_002: 2.69 crash -1.00 polar 0.00]
4	[ZINC00000706_003: 2.60 crash -0.48 polar 0.00]
5	[ZINC00000706_004: 2.51 crash -0.58 polar 0.00]
6	[ZINC00000706_005: 2.39 crash -0.17 polar 0.01]
7	[ZINC00000706_006: 1.83 crash -0.64 polar 0.00]
8	[ZINC00000706_007: 1.54 crash -0.36 polar 0.00]
9	[ZINC00000706_008: 1.29 crash -0.15 polar 0.00]
10	[ZINC00000706_009: 1.21 crash -0.15 polar 0.00]
11	[ZINC00000706_010: 1.16 crash -0.29 polar 0.00]
12	[ZINC00000706_011: 1.13 crash -0.30 polar 0.00]
13	[ZINC00000706_012: 1.10 crash -0.14 polar 0.00]
14	[ZINC00000706_013: 0.97 crash -0.32 polar 0.00]

15	[ZINC00000706_014: 0.92 crash -0.36 polar 0.00]
16	[ZINC00000706_015: 0.84 crash -0.14 polar 0.00]
17	[ZINC00000706_016: 0.78 crash -0.09 polar 0.00]
18	[ZINC00000706_017: 0.72 crash -0.31 polar 0.00]
19	[ZINC00000706_018: 0.71 crash -0.19 polar 0.00]
20	[ZINC00000706_019: 0.58 crash -0.28 polar 0.00]

Table S7. Molecular docking of berberine (ZINC03779067) to CmMLP1. The conformation with the highest binding affinity was marked in red. 20 conformations of berberine were presented according to the order of their binding affinity – from the highest to the lowest.

Berberine ZINC03779067: 43 atoms, 2 rot: [Time 5: srch 0 aln 1 m+c 1 pol 3] [Ncn 23: 3 25 2445 2075 74]	
No.	ZINC database no._conformation_no.: binding affinity, docking score
1	[ZINC03779067_000: 4.86 crash -0.37 polar 0.00]
2	[ZINC03779067_001: 4.58 crash -0.41 polar 0.00]
3	[ZINC03779067_002: 4.20 crash -0.54 polar 0.00]
4	[ZINC03779067_003: 4.18 crash -0.55 polar 0.00]
5	[ZINC03779067_004: 4.17 crash -0.63 polar 0.00]
6	[ZINC03779067_005: 4.14 crash -0.53 polar 0.00]
7	[ZINC03779067_006: 4.12 crash -0.48 polar 0.00]
8	[ZINC03779067_007: 4.11 crash -0.59 polar 0.00]
9	[ZINC03779067_008: 4.11 crash -0.57 polar 0.00]
10	[ZINC03779067_009: 4.07 crash -0.72 polar 0.00]
11	[ZINC03779067_010: 4.05 crash -1.23 polar 0.00]
12	[ZINC03779067_011: 3.91 crash -0.62 polar 0.00]
13	[ZINC03779067_012: 3.66 crash -0.98 polar 0.00]
14	[ZINC03779067_013: 3.61 crash -1.85 polar 0.00]
15	[ZINC03779067_014: 3.45 crash -0.28 polar 0.00]
16	[ZINC03779067_015: 3.41 crash -1.40 polar 0.00]
17	[ZINC03779067_016: 3.39 crash -0.28 polar 0.00]
18	[ZINC03779067_017: 3.38 crash -1.36 polar 0.00]
19	[ZINC03779067_018: 3.31 crash -0.77 polar 0.00]
20	[ZINC03779067_019: 3.30 crash -1.34 polar 0.00]

Table S8. Molecular docking of chelidonine (ZINC30727894) to CmMLP1. The conformation with the highest binding affinity was marked in red. 20 conformations of chelidonine were presented according to the order of their binding affinity – from the highest to the lowest.

Chelidonine ZINC30727894: 45 atoms, 1 rot: [Time 2: srch 0 aln 0 m+c 1 pol 1] [Ncn 23: 3 7 2067 2057 33]	
No.	ZINC database no._conformation_no.: binding affinity, docking score
1	[ZINC30727894_000: 2.14 crash -1.34 polar 0.00]
2	[ZINC30727894_001: 2.08 crash -0.42 polar 0.65]
3	[ZINC30727894_002: 2.03 crash -0.56 polar 0.07]
4	[ZINC30727894_003: 1.68 crash -0.53 polar 0.00]
5	[ZINC30727894_004: 1.68 crash -0.60 polar 0.12]
6	[ZINC30727894_005: 1.52 crash -0.40 polar 0.00]
7	[ZINC30727894_006: 1.47 crash -0.66 polar 0.34]
8	[ZINC30727894_007: 1.44 crash -1.42 polar 1.10]
9	[ZINC30727894_008: 1.27 crash -0.67 polar 0.00]
10	[ZINC30727894_009: 0.97 crash -0.31 polar 0.00]
11	[ZINC30727894_010: 0.93 crash -0.19 polar 0.00]
12	[ZINC30727894_011: 0.92 crash -0.36 polar 0.00]
13	[ZINC30727894_012: 0.89 crash -0.23 polar 0.00]
14	[ZINC30727894_013: 0.87 crash -0.24 polar 0.00]
15	[ZINC30727894_014: 0.85 crash -0.85 polar 0.00]
16	[ZINC30727894_015: 0.57 crash -0.98 polar 0.00]
17	[ZINC30727894_016: 0.54 crash -0.43 polar 0.00]
18	[ZINC30727894_017: 0.54 crash -0.81 polar 0.00]
19	[ZINC30727894_018: 0.53 crash -2.07 polar 0.00]
20	[ZINC30727894_019: 0.15 crash -3.55 polar 0.00]

Table S9. Molecular docking of protopine (ZINC20111233) to CmMLP1. The conformation with the highest binding affinity was marked in red. 20 conformations of protopine were presented according to the order of their binding affinity – from the highest to the lowest.

Protopine ZINC20111233: 45 atoms, 0 rot: [Time 2: srch 0 aln 0 m+c 0 pol 2] [Ncn 23: 3 5 889 919 54]	
No.	ZINC database no._conformation_no.: binding affinity, docking score
1	[ZINC20111233_000: 3.55 crash -1.09 polar 0.02]
2	[ZINC20111233_001: 3.27 crash -0.53 polar 0.93]
3	[ZINC20111233_002: 2.22 crash -0.36 polar 0.00]
4	[ZINC20111233_003: 2.17 crash -0.27 polar 0.00]
5	[ZINC20111233_004: 2.01 crash -1.45 polar 0.00]
6	[ZINC20111233_005: 1.96 crash -0.36 polar 0.00]
7	[ZINC20111233_006: 1.64 crash -0.30 polar 0.00]
8	[ZINC20111233_007: 1.63 crash -0.22 polar 0.00]
9	[ZINC20111233_008: 1.51 crash -0.38 polar 0.00]
10	[ZINC20111233_009: 1.50 crash -0.16 polar 0.00]
11	[ZINC20111233_010: 1.48 crash -0.34 polar 0.00]
12	[ZINC20111233_011: 1.47 crash -0.52 polar 0.00]
13	[ZINC20111233_012: 1.40 crash -0.48 polar 0.00]
14	[ZINC20111233_013: 1.31 crash -0.34 polar 0.00]
15	[ZINC20111233_014: 1.19 crash -0.47 polar 0.00]
16	[ZINC20111233_015: 1.16 crash -0.41 polar 0.00]
17	[ZINC20111233_016: 1.15 crash -0.30 polar 0.00]
18	[ZINC20111233_017: 0.84 crash -0.12 polar 0.00]
19	[ZINC20111233_018: 0.75 crash -0.14 polar 0.00]
20	[ZINC20111233_019: 0.72 crash -0.37 polar 0.00]

Table S10. Molecular docking of coptisine (ZINC01709414) to CmMLP1. The conformation with the highest binding affinity was marked in red. 20 conformations of coptisine were presented according to the order of their binding affinity – from the highest to the lowest.

Coptisine ZINC01709414: 38 atoms, 0 rot: [Time 3: srch 0 aln 0 m+c 1 pol 2] [Ncn 23: 3 5 301 331 53]	
No.	ZINC database no._conformation_no.: binding affinity, docking score
1	[ZINC01709414_000: 3.45 crash -0.18 polar 0.00]
2	[ZINC01709414_001: 3.43 crash -0.34 polar 1.03]
3	[ZINC01709414_002: 3.31 crash -0.25 polar 0.00]
4	[ZINC01709414_003: 3.20 crash -1.14 polar 0.00]
5	[ZINC01709414_004: 2.95 crash -0.32 polar 0.00]
6	[ZINC01709414_005: 2.93 crash -0.61 polar 0.00]
7	[ZINC01709414_006: 2.87 crash -1.04 polar 0.00]
8	[ZINC01709414_007: 2.80 crash -0.42 polar 0.00]
9	[ZINC01709414_008: 2.71 crash -1.00 polar 0.00]
10	[ZINC01709414_009: 2.63 crash -1.07 polar 0.00]
11	[ZINC01709414_010: 2.33 crash -0.41 polar 0.93]
12	[ZINC01709414_011: 2.26 crash -0.34 polar 0.00]
13	[ZINC01709414_012: 2.18 crash -0.37 polar 0.00]
14	[ZINC01709414_013: 2.07 crash -0.62 polar 0.00]
15	[ZINC01709414_014: 2.05 crash -0.62 polar 0.00]
16	[ZINC01709414_015: 2.04 crash -0.42 polar 0.00]
17	[ZINC01709414_016: 2.03 crash -0.79 polar 0.00]
18	[ZINC01709414_017: 1.97 crash -1.15 polar 0.00]
19	[ZINC01709414_018: 1.94 crash -0.64 polar 0.00]
20	[ZINC01709414_019: 1.91 crash -0.77 polar 0.00]

Table S11. Molecular docking of dihydroberberine (ZINC01575028) to CmMLP1. The conformation with the highest binding affinity was marked in red. 20 conformations of dihydroberberine were presented according to the order of their binding affinity – from the highest to the lowest.

Dihydroberberine ZINC01575028: 44 atoms, 2 rot: [Time 4: srch 0 aln 0 m+c 1 pol 3] [Ncn 23: 3 25 2445 2075 73]	
No.	ZINC database no._conformation_no.: binding affinity, docking score
1	[ZINC01575028_000: 5.98 crash -0.55 polar 1.14]
2	[ZINC01575028_001: 5.72 crash -0.64 polar 1.18]
3	[ZINC01575028_002: 5.64 crash -0.56 polar 1.18]
4	[ZINC01575028_003: 5.64 crash -0.54 polar 1.18]
5	[ZINC01575028_004: 5.38 crash -0.70 polar 1.18]
6	[ZINC01575028_005: 4.68 crash -0.79 polar 0.00]
7	[ZINC01575028_006: 4.23 crash -0.63 polar 0.00]
8	[ZINC01575028_007: 4.15 crash -0.65 polar 0.00]
9	[ZINC01575028_008: 4.08 crash -0.49 polar 0.00]
10	[ZINC01575028_009: 3.90 crash -1.19 polar 0.00]
11	[ZINC01575028_010: 3.73 crash -0.76 polar 0.00]
12	[ZINC01575028_011: 3.71 crash -0.87 polar 0.00]
13	[ZINC01575028_012: 3.66 crash -0.80 polar 0.00]
14	[ZINC01575028_013: 3.60 crash -0.87 polar 0.00]
15	[ZINC01575028_014: 3.59 crash -1.01 polar 0.00]
16	[ZINC01575028_015: 3.52 crash -0.41 polar 0.00]
17	[ZINC01575028_016: 3.42 crash -2.19 polar 0.00]
18	[ZINC01575028_017: 3.40 crash -3.17 polar 0.00]
19	[ZINC01575028_018: 3.33 crash -3.02 polar 0.00]
20	[ZINC01575028_019: 3.16 crash -0.34 polar 0.00]

Table S12. Molecular docking of 8-hydroxycheleritrine (ZINC03872044) to CmMLP1. The conformation with the highest binding affinity was marked in red. 20 conformations of 8-hydroxycheleritrine were presented according to the order of their binding affinity – from the highest to the lowest.

8-hydroxycheleritrine ZINC03872044: 44 atoms, 2 rot: [Time 4: srch 0 aln 0 m+c 1 pol 3] [Ncn 23: 3 25 2445 2075 71]	
No.	ZINC database no._conformation_no.: binding affinity, docking score
1	[ZINC03872044_000: 5.94 crash -0.50 polar 0.00]
2	[ZINC03872044_001: 5.93 crash -0.52 polar 0.00]
3	[ZINC03872044_002: 5.93 crash -0.50 polar 0.01]
4	[ZINC03872044_003: 5.86 crash -0.52 polar 0.00]
5	[ZINC03872044_004: 5.84 crash -0.50 polar 0.00]
6	[ZINC03872044_005: 5.77 crash -0.82 polar 0.01]
7	[ZINC03872044_006: 5.76 crash -0.59 polar 0.01]
8	[ZINC03872044_007: 5.76 crash -0.80 polar 0.01]
9	[ZINC03872044_008: 5.74 crash -0.65 polar 0.01]
10	[ZINC03872044_009: 5.72 crash -0.52 polar 0.00]
11	[ZINC03872044_010: 5.65 crash -0.49 polar 0.01]
12	[ZINC03872044_011: 5.49 crash -0.45 polar 0.01]
13	[ZINC03872044_012: 5.42 crash -1.22 polar 0.13]
14	[ZINC03872044_013: 5.17 crash -0.69 polar 0.01]
15	[ZINC03872044_014: 5.10 crash -0.69 polar 0.01]
16	[ZINC03872044_015: 5.09 crash -0.71 polar 0.01]
17	[ZINC03872044_016: 5.05 crash -0.51 polar 0.01]
18	[ZINC03872044_017: 5.04 crash -0.57 polar 0.01]
19	[ZINC03872044_018: 4.97 crash -0.63 polar 0.01]
20	[ZINC03872044_019: 4.93 crash -0.60 polar 0.01]

Table S13. Molecular docking of stylopine (ZINC20470298) to CmMLP1. The conformation with the highest binding affinity was marked in red. 20 conformations of stylopine were presented according to the order of their binding affinity – from the highest to the lowest.

Stylopine ZINC20470298: 41 atoms, 0 rot: [Time 2: srch 0 aln 0 m+c 1 pol 1] [Ncn 23: 3 5 637 667 54]	
No.	ZINC database no._conformation_no.: binding affinity, docking score
1	[ZINC20470298_000: 2.84 crash -1.44 polar 0.00]
2	[ZINC20470298_001: 2.55 crash -0.77 polar 0.00]
3	[ZINC20470298_002: 2.48 crash -0.41 polar 0.00]
4	[ZINC20470298_003: 2.17 crash -0.38 polar 0.00]
5	[ZINC20470298_004: 2.05 crash -0.80 polar 0.00]
6	[ZINC20470298_005: 1.91 crash -0.45 polar 0.00]
7	[ZINC20470298_006: 1.86 crash -0.44 polar 0.00]
8	[ZINC20470298_007: 1.81 crash -1.79 polar 0.00]
9	[ZINC20470298_008: 1.66 crash -1.01 polar 0.00]
10	[ZINC20470298_009: 1.63 crash -0.20 polar 0.00]
11	[ZINC20470298_010: 1.45 crash -1.19 polar 0.00]
12	[ZINC20470298_011: 1.38 crash -0.67 polar 0.00]
13	[ZINC20470298_012: 1.36 crash -0.29 polar 0.00]
14	[ZINC20470298_013: 1.34 crash -0.31 polar 0.00]
15	[ZINC20470298_014: 1.34 crash -0.40 polar 0.00]
16	[ZINC20470298_015: 1.28 crash -0.82 polar 0.00]
17	[ZINC20470298_016: 1.21 crash -0.70 polar 0.00]
18	[ZINC20470298_017: 1.17 crash -0.53 polar 0.00]
19	[ZINC20470298_018: 1.16 crash -0.24 polar 0.00]
20	[ZINC20470298_019: 1.08 crash -0.50 polar 0.00]

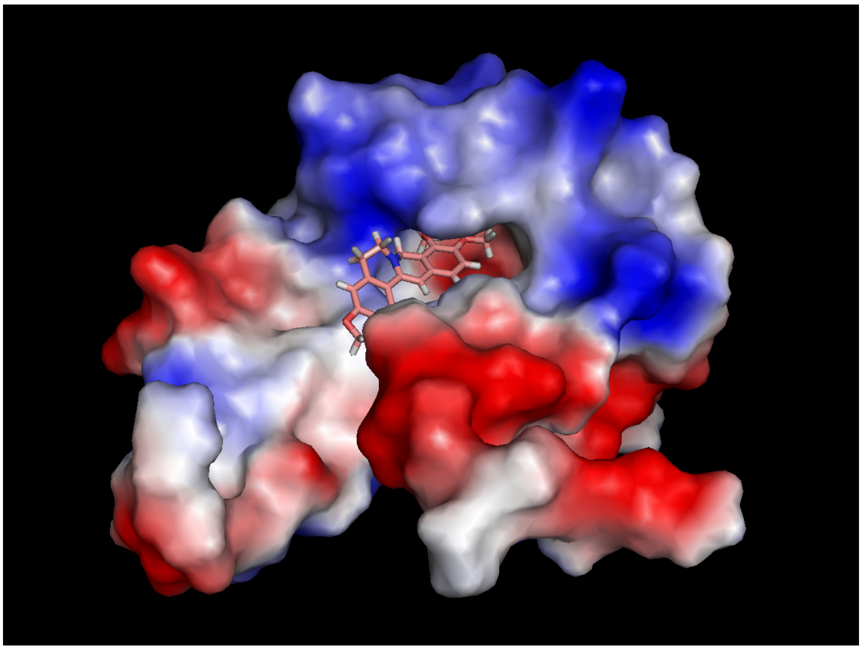

Figure S50. 3D model of *C. majus* MLP in the surface representation, colored according to the distribution of the electrostatic surface potential calculated with ABPS (PyMol). Positively charged regions in blue, negatively – in red. Hydrophobic cavity filled by dihydroberberine [ZINC01575028_000: 5.98 crash -0.55 polar 1.14] (source: PyMol).

Supplementary Figures 51-52: Viability of HeLa and C33A cancer cells after incubation with fractions from *C. majus* whole plant extract.

HeLa, C33A and MSU-1.1 cells were exposed to 6 flow-through (FT6-11) fractions obtained from crude *C. majus* extracts. C33A, HeLa and MSU-1.1 were treated with 8 heparin selected protein fractions (18-26) (Supplementary Figures 51, 52). To investigate the cytotoxicity effect of flow-through fractions and heparin separated samples in HeLa, C33A, MsU-1.1 cell lines, the WST-1 assay was carried out. No significant decrease in cell viability was observed in fibroblast cells exposed to fractions (FT6-11) and heparin selected protein fractions (18-23). By contrast, our results showed significant decrease in the cell viability of C33A cell line after incubation cells with 23, 24, 25, 26 heparin protein fractions. As shown in Supplementary Figures 51 the viability of cervical cancer HPV negative C33A cells was significantly reduced. We noticed the C33A cell viability at the level of 50% (IC50) for fraction number 23. We noticed a rapid decrease of C33A cell viability in the range of 24 to 26 heparin protein fractions.

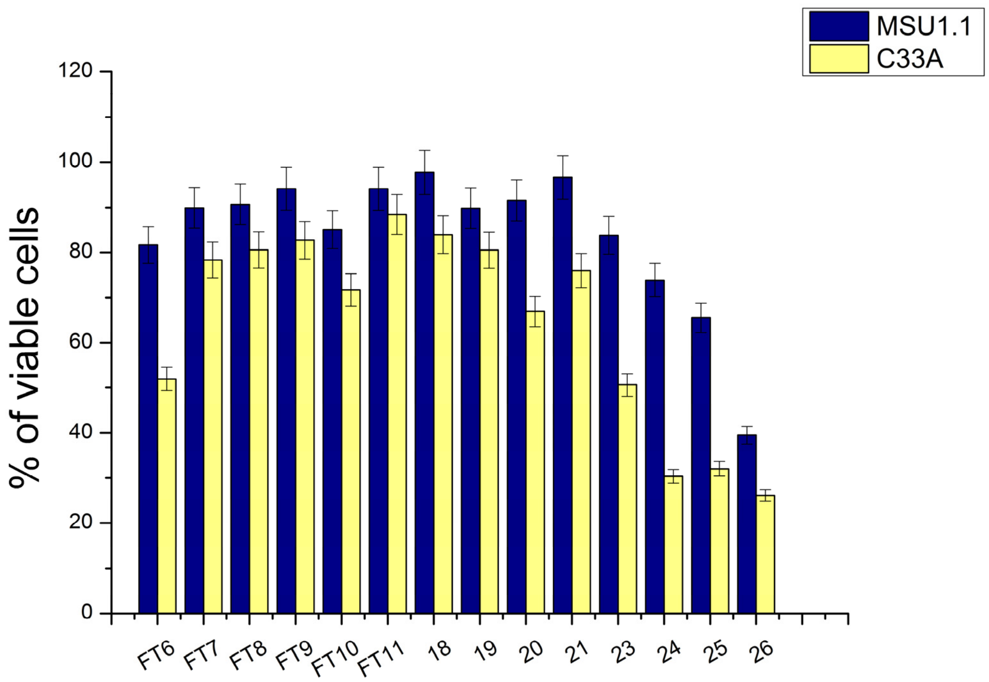

Figure S51. Effect of flow-through (FT6-11) and heparin separated protein fractions from *C. majus* whole plant extract on cell viability in cervical cancer cells (C33A) and fibroblasts (MSU-1.1).

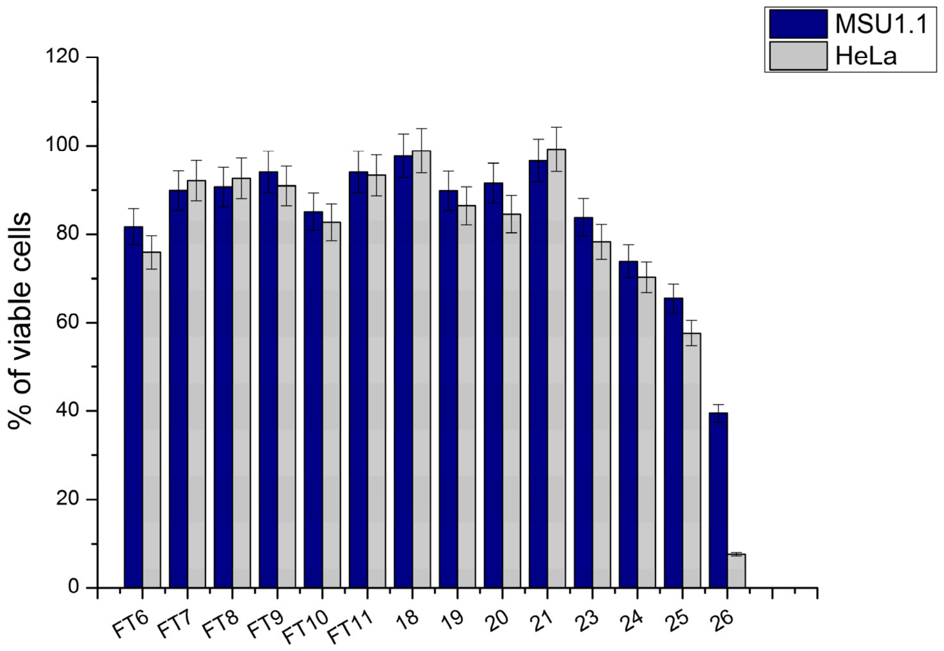

Figure S52. Effect of flow-through (FT6-11) and heparin separated protein fractions from *C. majus* whole plant extract on cell viability in cervical cancer cells (HeLa) and fibroblasts (MSU-1.1).
